# Experimental verification about treatment of Bu-Shen-Yi-Jing-Fang in Alzheimer’s disease by the analysis of the feasible signaling pathway of network pharmacology

**DOI:** 10.1186/s12906-024-04527-w

**Published:** 2024-06-08

**Authors:** Yingchao Hu, Renjuan Hao, Deyu Li, Yunwei Lu, Guran Yu

**Affiliations:** Department of Neurology, Jiangsu Province Hospital of Chinese Medicine, The Affiliated Hospital of Nanjing University of Chinese Medicine, Nanjing, Jiangsu Province 210001 China

**Keywords:** Apoptosis, Ferroptosis, PI3K/AKT/Nrf2

## Abstract

**Context:**

Bu-shen-yi-jing-fang (BSYJF) has been reported to reduce amyloid-β (Aβ)_1–42_ deposition in the brain of APP/PS1 mice and ameliorate cognitive function. However, its neuroprotective mechanism remains unclear.

**Objective:**

This study aims to investigate whether BSYJF exerts a protective effect on Aβ_1–42_-induced oxidative stress injury and explore its possible mechanism.

**Materials and methods:**

The platform databases TCMSP, Swiss, TTD, DrugBank, and GeneCards were used to mine the targets of Alzheimer’s disease (AD) and BSYJF. The platform databases STRING and Metascape were used to build the interaction network of the target protein, and Cytoscape software was used to analyze this network and screen out the key pathways. Aβ_1–42_-treated SKNMC cells were established to verify the mechanism of BSYJF and the key proteins. The downstream proteins and antioxidants as well as apoptosis and ferroptosis of the PI3K/AKT/Nrf2 signaling pathway were validated using an in vitro SKNMC cell model experiment. The expression levels of related proteins were detected using Western blotting. Flow cytometry and immunofluorescence staining were used to analyze apoptosis and ferroptosis.

**Results:**

Kyoto encyclopedia of genes and genomes (KEGG) pathway enrichment analysis considered the key signal pathways, mainly involving the PI3K/AKT signaling pathway. Experimental validation demonstrated that BSYJF treatment markedly increased the activity of the PI3K/AKT pathway, which could exert anti-AD effects.

**Conclusions:**

Our data provided compelling evidence that the protective effects of BSYJF might be associated with their regulation of the PI3K/AKT/Nrf2 signaling pathway. These studies offered a potential therapy for natural herbal medicine treatment of AD.

**Supplementary Information:**

The online version contains supplementary material available at 10.1186/s12906-024-04527-w.

## Introduction

Alzheimer’s disease (AD) is a neurodegenerative disease caused by a variety of genetic factors, environmental factors, and advanced aging factors. With more than 8 million AD patients, China reportedly has the largest number of AD patients in the world [1]. The amyloid cascade hypothesis and tau phosphorylation are recognized as the prime pathological mechanisms of AD. Reportedly, most international clinical trial drugs for amyloid-β (Aβ) protein and tau protein are ineffective [2]. Mounting evidence indicated that Aβ was expressed in the brain of AD patients and that elevated levels of Aβ in the brain had pathogenic consequences [3]. Currently, clinical treatments primarily focus on symptoms, while drugs that improve patients’ quality of life and outcomes have yet to be studied and evaluated. The exploration of traditional Chinese medicine (TCM) has obvious advantages in improving the condition of AD, and TCM is gradually gaining prominence for the treatment of this disease [4].

According to the theory of TCM, the pathogenesis of AD is based on “kidney deficiency as the base and phlegm turbidity and blood stasis as the standard”. A preliminary clinical study reported that Bu-shen-yi-jing-fang (BSYJF) had delayed AD progression and mild cognitive impairment and exerted a neuroprotective effect. BSYJF is an empirical formula for AD treatment, which has been clinically used for more than a decade. It contains *Rehmannia glutinosa* (shudihuang/shengdi, SDH/SD), *Cornus officinalis* (Shanzhuyu, SZY), *Cuscuta chinensis* Lam (Tusizi, TSZ), *Panax ginseng* (Renshen, RS), *Atractylodes macrocephala* (Baishu, BS), *Corethrodendron multijugum* (Huangqi, HQ), and *Piper kadsura* (Haifengteng, HFT). Except for *C. officinalis*, which is the ripe pulp, *C. chinensis*, which refers to mature seeds, and *P. kadsura*, which is the stem of the plant, all other herbs involved in BSYJF are the roots of plants. *R. glutinosa*, *C. officinalis* (Shanzhuyu, SZY), and *C. chinensis* Lam exerted marked effects, such as protecting the blood–brain barrier (BBB), reducing age spots, and improving cognition in AD mice. *P. ginseng*, *A. macrocephala*, and *C. multijugum* displayed obvious effects, including phlegm reduction. *P. kadsura* played a role in promoting blood circulation, removing blood stasis, and clearing collaterals. Catalpol (the active ingredient in *R. glutinosa*), cycloastragenol (the active component in *C. multijugum*), and hyperoside (one of the active ingredients of *C. chinensis*) could reduce BBB permeability induced by Aβ_1–42_ in vitro [5–7]. Morroniside, one of the components of *C. officinalis*, could inhibit H_2_O_2_/Aβ_1–42_-induced cell apoptosis by suppressing JNK and p38 MAPK phosphorylation [8]. Multiple studies on the effect of various Chinese medicine components on AD have been published in Chinese journals. Currently, a clinical cohort study, namely, BSYJF Intervention in early Alzheimer’s Disease Prospective Cohort Study, for AD treatment is being conducted in the Jiangsu Province Hospital of Chinese Medicine (Registration number of Chinese Clinical Trials Registry: ChiCTR2000040502). However, the neuroprotective mechanism of BSYJF remains unclear. That is to say, the BSYJF is a compound of traditional Chinese medicine summarized through the classics and experience of traditional Chinese medicine. Although it has been studied in clinical experiments, the ingredient still needs to be further applied in basic experimental research to prove the curative effect of the compound. So that the Chinese herbal medicine is worthy of research and promotion.

**Fig. 1:**
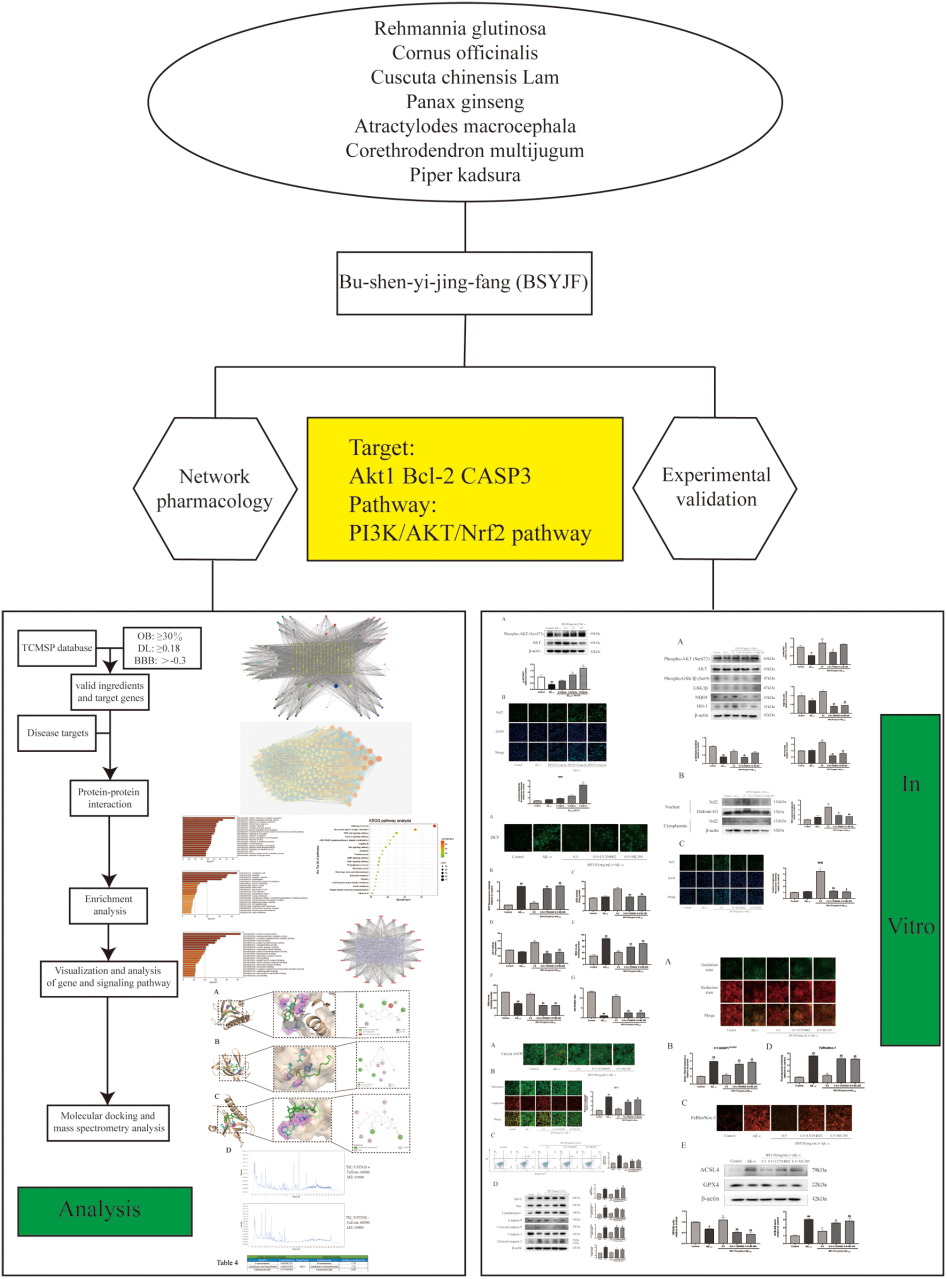
Graphical abstract

Based on the massive information regarding the active ingredients of BSYJF and disease gene targets, the network pharmacology on the analysis of KEGG enrichment function systematically elaborates the mechanism of AD, which puts forward a scientific theoretical basis and reference for AD. As mentioned above, BSYJF has been applied clinically in Traditional Chinese Medicine Hospital of Jiangsu Province, and its pharmacology involves neuroprotective, anti-aging, and antioxidant effects, among other aspects. In this study, we assessed the feasibility of the treatment of AD through experimental verification. Our study provides certain theoretical support and a scientific basis for exploring the mechanism and developing drugs related to AD. In the current field, on the one hand, the composition of Chinese herbal medicine is complex, but the main chemical components were studied and recognized. On the other hand, human gene-target protein prediction on pathogenic mechanism is a relatively mature system. Namely, we can establishing the relationship between drug target and disease-gene products to analyze the validation of traditional Chinese medicine to the disease. We analyzed the pharmacological effects of BSYJF through network pharmacology and carried out corresponding experimental verification (Fig. 1). We have known that Aβ production and accumulation in the brain have various harmful effects. Therefore, we used Aβ_1–42_ to treat cells to explore and verify the protective effect of BSYJF on AD and its potential mechanism to provide a theoretical basis and support for the clinical application of TCM to AD.

## Materials and methods

### Data extraction

The active ingredients and targets of the drug such as shudihuang/shengdi, shanzhuyu, tusizi, renshen, huangqi, baishu, and haifengteng were fetched through the TCMSP database [9] by prerequisites including oral bioavailability (OB) > 30%, drug-like (DL) > 0.18, BBB > − 0.3. The ingredients of shengdi were found in the literature and imported into the database of Swiss ADME [10] and filtered by the good properties containing “GI Absorption: High”, “BBB permeant: Yes” as well as “Druglikeness: Yes” in pharmacokinetics. The valid ingredients and targets corresponding to proteins in the UniProt database were effectively screened out and labeled to arrive at the corresponding target genes. “Alzheimer’s disease” was used as a keyword in the platform databases TTD [11], DrugBank [12], and geneCards [13] to obtain disease targets. Then, the intersection of the ingredient and disease targets as a therapeutic target was collected for AD treatment via the Venny online tool.

### Protein–protein interaction (PPI)

The STRING [14] platform data were applied for protein–protein interaction concerning the targets of treatment of the traditional Chinese medicine for AD. The species were set to “*Homo sapiens*” when using the platform, and the results of the analysis obtained from its platform database were preserved. The PPI network analysis was carried out using Cytoscape [15].

### Enrichment analysis

The Metascape [16] database was used to analyze the role of BSYJF in AD treatment regarding efficacious targets of GO and KEEG. The genes corresponding to the target protein of the active ingredient and the genes of AD were entered into its database. The GO function enrichment analysis and KEEG signaling pathway enrichment analysis were carried out respectively to investigate the functional distribution and the signal regulation pathways associated with AD that could be applied. A network of target-signaling pathways for BSYJF to treat AD was determined using the Cytoscape software, and the collected “drug composition–disease target–signaling pathway” information was imported into the analysis.

### Visualization and analysis of drug–target gene

Cytoscape [15] was used to visualize the results of our analysis. The gene mapping protein of the drug acquired by UniProt and DrugBank was imported into the software. Each node represents a signal target gene, and the edges represent the interaction between different nodes. The more edges indicate that the nodes were more critical in the network. The size of the node represents the degree value of the node, and the thickness of the edge represents the action strength between different nodes.

### Molecular docking and mass spectrometry analysis

To discover the compounds with potential effects on AD, molecular docking was performed to predict the interaction. The crystal structure preparation of the protein was found in the Research Collaboratory for Structural Bioinformatics (RCSB) database and the PDB library. The natural compounds were downloaded from the PubChem database. Subsequently, they were processed to construct the structure. The conformations were performed to study the interactions between ligands and the target receptor by using AutoDock 4.2 (MGL tools1.5.6). Next, they were analyzed by Discovery Studio Visualizer and PyMOL. To analyze and identify the ligands, a mass spectrometry analysis of the chemical constituents in the extracts of BSYJF was performed using liquid chromatography-tandem mass spectrometry (LC-MS/MS) analysis in the positive/negative ion mode (Thermo Fisher, USA), which was performed on an ultra-high-performance liquid chromatography (UHPLC) system (Vanquish, Thermo Fisher Scientific) with a Waters UPLC BEH C18 column.

### Preparation of materials

The herbs were purchased from Jiangsu Hospital of Traditional Chinese Medicine (Nanjing, China). BSYJF is a mixture of the proportion of each plant (SDH 40 g, SD 20 g, SZY 30 g, TSZ 15 g, RS 10 g, BS 10 g, HQ 30 g, and HFT 15 g). The raw herbal materials were extracted for 2 h with boiling distilled water under reflux. The extraction was repeated once. Then, the aqueous extract of BSYJF was filtered, concentrated, and lyophilized into a freeze-dried powder and stored at − 20 ℃ before use. The powder was dissolved in PBS (100 mg/mL) and stored at − 80 ℃. 3-(4,5)-Dimethylthiahiazo (-z-y1)-3,5-di-phenytetrazoliumromide (MTT) and dimethyl sulfoxide (DMSO) were obtained from Sigma-Aldrich (St. Louis, MO, USA). Reactive oxygen species (ROS), lipid peroxidation (MDA), catalase (CAT), total superoxide dismutase (SOD), DAPI staining solution, and mitochondrial membrane potential probe (JC-1 assay kit) were acquired from Beyotime Biotechnology (Beijing, China). Annexin V- FITC/PI apoptosis assay kit was acquired from Vazyme Biotechnology (Nanjing, China). Cell viability/cytotoxicity detection kit (Calcein-AM/PI) was bought from KeyGEN BioTECH (Nanjing, China). Minimum essential medium (MEM) and fetal bovine serum (FBS) were obtained from Gibco (Grand Island, NY, USA). Primary antibodies against caspase-3 (#66470-2-Ig), BAX (#50599-2-Ig), Bcl2 (#12789-1-AP), HO-1 (#10701-1-AP), ACSL4 (#22401-1-AP), AKT (51077-1-AP), phospho-AKT (Ser473) (#66444-1-Ig), histone-H3 (#17168-1-AP), and β-actin (#66009-1-Ig) were bought from Proteintech (Wuhan, China). Antibodies against caspase-9 (#9508) and cytochrome C (#11,940) were purchased from Cell Signaling Technology (Beverly, MA, United States). The antibody against GPX4 (ET1706-45) was procured from HUABIO (Hangzhou, China). The antibody against Nrf2 (ab137550) was obtained from Abcam (Cambridge, England). The antibody against NQO1 (AF301136), GSK3β (AF04957), and GSK3β (phosphor ser9) (AF00575) was bought from AiFang Biological (Hunan, China).

### LC-MS/MS analysis

An LC-MS/MS analysis was performed to determine the chemical constituents in the extracts of BSYJF. The analysis was performed on a UHPLC system (Vanquish, Thermo Fisher Scientific). An Orbitrap Exploris 120 mass spectrometer coupled with Xcalibur software was employed to obtain the MS and MS/MS data based on the IDA acquisition mode. And the corresponding MS/MS data were further acquired. Results were obtained according to reliable matches based on the BIOTREE database by BIOTREE Corporation.

### Cell culture

Human SKNMC neuroepithelioma cells were purchased from Fu Heng Biology (Shanghai, China). The cell medium contains 10% FBS, 100 U/mL penicillin, 100 mg/mL streptomycin, and 100 mg/mL amphotericin cultured in a 37 ℃, 5% CO_2_/95% air incubator. The Aβ_1–42_ peptide (Cell Signaling Technology, USA) was dissolved in hexafluoroisopropanol to obtain monomer Aβ_1–42_. The monomer was then dissolved with DMSO and PBS and incubated at 4 °C for 24 h. Finally, MEM was added to form Aβ_1–42_ oligomer and stored at − 80 ℃ for 200 µM.

### Western blot

The protein levels of total AKT, phospho-AKT, GSK3β, phospho-GSK3β, Nrf2, NQO1, HO-1, cytochrome C, cleaved caspase 9/caspase 9, cleaved caspase 3/caspase 3, BAX and Bcl2, ACSL4, and GPX4 were detected using western blot assay. After appropriate treatments, the cells were collected, and the proteins were extracted according to instructions. Protein samples were loaded on 8–15% resolving SDS-PAGE gel and transferred onto a PVDF membrane. Membranes were blocked in 5% non-fat milk. The blocked membranes were washed with TBST thrice for 10 min and incubated with the primary antibody overnight at 4 °C. Subsequently, the membrane was further incubated with horseradish peroxidase‑conjugated anti-mouse/rabbit secondary antibodies (1:3000, Servicebio, Wuhan, China) for 1 h at room temperature. Finally, the specific bands were visualized by using ECL chemiluminescence (Biosharp, Shanghai, China) and imaged using a Gel Imager System (CHEMIDOC XRS+, BIO-RED, USA). Band density was quantified by Image Lab software. β-actin served as the internal control, and all protein expression levels were normalized to β-actin.

### Immunofluorescence staining

SKNMC cells were cultured in 12-well plates with a density of 1 × 10^5^/cm^2^. After adequate treatment, cells were washed with ice-cold PBS. Then, cells were fixed with 4% paraformaldehyde for 10 min, permeabilized with 0.3% Triton X-100 for 30 min, and blocked with Immunol Staining Blocking Buffer (Beyotime, China) for 60 min at room temperature. Primary antibodies anti-Nrf2 (1:200, Proteintech) were incubated (overnight, 4 ℃) and washed with PBS thrice. The cells were incubated with coupled fluorescent-labeled secondary antibodies (1:1000, Proteintech) followed by 60 min of dark incubation at room temperature. The nucleus was stained with DAPI for 10 min. Images were captured with a fluorescence microscope (DS-Qi2, NIKON, Japan).

### ROS detection with DCFH-DA assay

DCFH-DA, 2′7′-dichloro-dihydro-fluorescein diacetate, is a non-fluorescent cell-permeable molecule, which is the most widely used probe for the detection of oxidative species in living cells. 2′,7′-dichloro-dihydro-fluorescein (DCFH) is produced by the hydrolysis of the acetate groups by intracellular esterase. The high fluorescence of ROS generates, in which mainly hydroperoxides oxidize DCFH to DCF [17]. Cells were resuspended with diluted DCFH-DA. After suspension, all samples were incubated for 20 min at 37 °C. Images were obtained with an upright fluorescence microscope, and fluorescence intensity was analyzed using the ImageJ software.

### The detection of SOD/CAT/MDA/ GSH/GSSG activity

SKNMC cells were cultured in well plates with a density of 1 × 10^5^/cm^2^. The cells were collected and lysated with RIPA or SOD sample solution and were then centrifuged and extracted. The supernatant was collected under centrifugation at 12,000 rpm and 4 °C for 20 min. According to kit instructions, the SOD, CAT, and MDA activities were measured. Glutathione activity was assayed by using a glutathione assay kit in accordance with the manufacturer’s instructions. A BCA protein assay kit (Beyotime, China) was used to determine protein concentration.

### Calcein-AM/Propidium iodide (PI) staining

After different treatments, the cells were collected by trypsinization and centrifuged. Cells were washed thrice with ice-cold PBS. The cells were labeled with Calcein-AM/PI for 30 min at room temperature. After that, the living cells (green fluorescence) and dead cells (red fluorescence) were observed under the fluorescence microscope.

### JC-1 mitochondrial membrane potential assay

According to the manufacturer’s instructions, cells treated with different concentrations of drugs were collected and then incubated with JC-1 in a cell incubator for 20 min. Image analysis was performed with an upright fluorescence microscope and the software ImageJ.

### Flow cytometric analysis of apoptosis

Apoptotic cells were quantified using flow cytometry in cell suspensions after dissociation. Apoptosis was detected with Annexin V-FITC/PI staining kit following the manufacturer’s instructions (Roche, Swiss). Then, the cells were collected and washed thrice with PBS. After centrifugation, the cells were resuspended in 100 µL labeling solution and 5 µL Annexin V-FITC/PI. Then, all samples were incubated at room temperature for 10 min, and a 400 µL binding buffer (1x) was added. Apoptotic activities of cells were quantified via flow cytometry (FACS Celesta, BD Biosciences), and the data were analyzed using FlowJo.

### Detection of cellular lipid peroxidation

C11-BODIPY^581/591^ is a lipophilic fluorescent probe that is an effective tool for estimating lipid oxidation and antioxidant efficacy in cells. After appropriate treatment and loading with C11-BODIPY^581/591^, the cells were completely washed with PBS. Images were captured with the fluorescence microscope and ImageJ. The green fluorescence was divided by the total fluorescence (green + red) to obtain the fraction of oxidized C11 BODIPY^581/591^. The green/red fluorescence ratio was used as a measurement of lipid peroxidation.

### Detection of intracellular Fe^2+^ deposition

The SKNMC cells were cultured on coverslips in 12-well plates. After drug treatment, the cells were washed thrice with ice-cold PBS and incubated with 5 µM FeRhonox-1 at 37 ℃ for 1 h. Then, the cells were completely washed with PBS. Images were taken with the fluorescence microscope and measured using ImageJ.

### Statistical analysis

All data were expressed as means ± standard error of the mean (SEM) and followed a normal distribution. All experiments were repeated thrice independently. Comparisons were performed by one-way analysis of variance and followed by Tukey’s multiple comparison post-hoc test or Dunnett’s T3 post-hoc test. Statistical analysis was performed using SPSS 22.0 software (SPSS Inc., Chicago, IL) and GraphPad Prism 6.0 (GraphPad Software, La Jolla, CA). For all tests, differences were considered significant when *p* < 0.05.

## Results

### The active ingredients and coupled targets of BSYJF and their relationship with AD genes

**Fig. 2:**
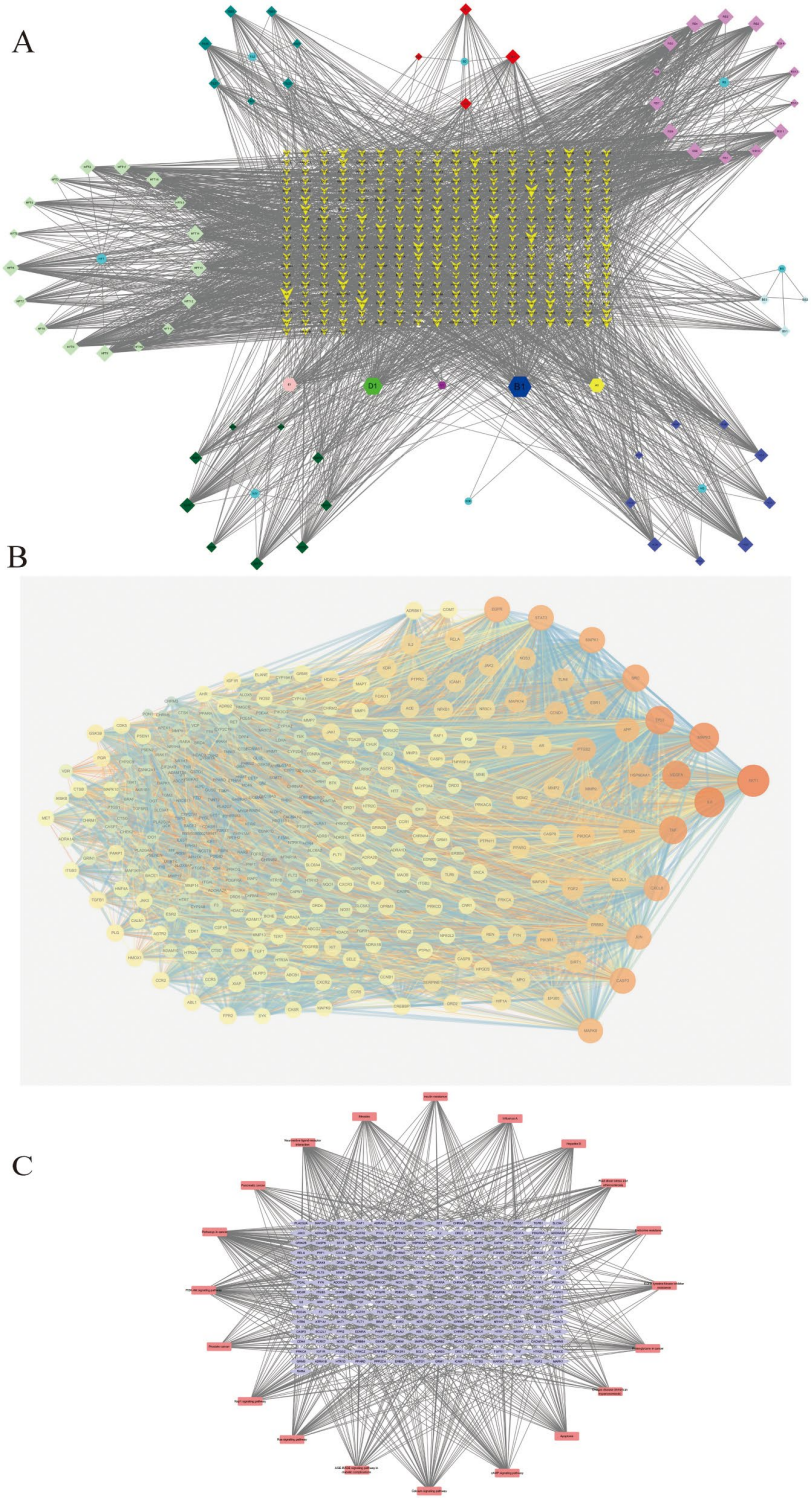
(**A**) Chemical components of the compound on AD’s targets. The colors were coupled with chemical components of compounds, except purple. The purple represents 346 targets, removing overlapping targets of each component and anti-AD targets of BSYJF. (**B**) Network of protein–protein interaction includes 346 target protein nodes with a total of 7542 interaction lines. (**C**) The network of ingredient–target–pathway contained a total of 53 nodes, 263 overlapping targets of composition–disease, and 20 signal path nodes

Through the selection of valid conditions, there had been 77 active ingredients of eight herbs. The chemical composition–action target data are specified in Table 1. For the 77 components, the targets were obtained from Swiss Target Prediction. The overlapping targets of each component were removed. The 913 targets were mapped to human homologous genes through the UniProt database. Through the disease database, the term “Alzheimer’s disease” was used to find 1520 of its relevant targets. Through the Venn diagram, the target intersection of anti-AD targets of BSYJF was combined with 913 targets, and 346 targets were acquired. These 346 targets were potentially effective targets of BSYJF in AD. The relationships of drug–composition and disease–target were visualized using the Cytoscape software (Fig. 2A).

**Table 1:** Active components of compound

The active ingredients of Bushenyijinfang						
Chinese Name	English Name	OB	BBB	DL	Coding	MOL ID
Shudihuang	sitosterol	36.91	0.87	0.75	A1	MOL000359
	Stigmasterol	43.83	1	0.76	B1	MOL000449
Shanzhuyu	Mandenol	42	1.14	0.19	SZY1	MOL001494
	Ethyl linolenate	46.1	1.12	0.2	SZY2	MOL001495
	poriferast-5-en-3beta-ol	36.91	1.14	0.75	SZY3	MOL001771
	Diop	43.59	0.26	0.39	C1	MOL002879
	Ethyl oleate (NF)	32.4	1.1	0.19	SZY4	MOL002883
	beta-sitosterol	36.91	0.99	0.75	D1	MOL000358
	sitosterol	36.91	0.87	0.75	A1	MOL000359
	Stigmasterol	43.83	1	0.76	B1	MOL000449
	2,6,10,14,18-pentamethylicosa-2,6,10,14,18-pentaene	33.4	1.99	0.24	SZY5	MOL005481
	3,4-Dehydrolycopen-16-al	46.64	0.6	0.49	SZY6	MOL005486
	Cornudentanone	39.66	0.09	0.33	SZY7	MOL005503
	Tetrahydroalstonine	32.42	0.33	0.81	SZY8	MOL008457
	lanosta-8,24-dien-3-ol,3-acetate	44.3	1.31	0.82	SZY9	MOL005557
Tusizi	sesamin	56.55	-0.08	0.83	TSZ1	MOL001558
	NSC63551	39.25	1.22	0.76	TSZ2	MOL000184
	beta-sitosterol	36.91	0.99	0.75	D1	MOL000358
	campest-5-en-3beta-ol	37.58	0.94	0.71	TSZ3	MOL005043
	Isofucosterol	43.78	0.97	0.76	TSZ4	MOL005440
	matrine	63.77	1.52	0.25	TSZ5	MOL005944
	sophranol	55.42	0.68	0.28	TSZ6	MOL006649
	CLR	37.87	1.13	0.68	TSZ7	MOL000953
Renshen	Diop	43.59	0.26	0.39	C1	MOL002879
	Stigmasterol	43.83	1	0.76	B1	MOL000449
	beta-sitosterol	36.91	0.99	0.75	D1	MOL000358
	Inermin	65.83	0.36	0.54	RS1	MOL003648
	Aposiopolamine	66.65	0.4	0.22	RS2	MOL005308
	Celabenzine	101.88	0.05	0.49	RS3	MOL005314
	Deoxyharringtonine	39.27	-0.25	0.81	RS4	MOL005317
	arachidonate	45.57	0.58	0.2	RS5	MOL005320
	Frutinone A	65.9	0.46	0.34	RS6	MOL005321
	Ginsenoside-Rh4	31.11	-0.18	0.78	RS7	MOL005348
	Girinimbin	61.22	1.22	0.31	RS8	MOL005356
	Gomisin B	31.99	0.18	0.83	RS9	MOL005357
	Panaxadiol	33.09	0.23	0.79	RS10	MOL005376
	suchilactone	57.52	0.28	0.56	RS11	MOL005384
	alexandrin	36.91	0.88	0.75	RS12	MOL005399
	ginsenoside Rg5	39.56	0.21	0.79	RS13	MOL005401
	Fumarine	59.26	-0.13	0.83	RS14	MOL000787
Huangqi	Mairin	55.38	0.22	0.78	HQ1	MOL000211
	Jaranol	50.83	-0.22	0.29	HQ2	MOL000239
	hederagenin	36.91	0.96	0.75	HQ3	MOL000296
	(3 S,8 S,9 S,10R,13R,14 S,17R)-10,13-dimethyl-17-[(2R,5 S)-5-propan-2-yloctan-2-yl]-2,3,4,7,8,9,11,12,14,15,16,17-dodecahydro-1 H-cyclopenta[a]phenanthren-3-ol	36.23	1.09	0.78	E1	MOL000033
	3,9-di-O-methylnissolin	53.74	0.63	0.48	HQ4	MOL000371
	7-O-methylisomucronulatol	74.69	0.84	0.3	HQ5	MOL000378
	(6aR,11aR)-9,10-dimethoxy-6a,11a-dihydro-6 H-benzofurano[3,2-c]chromen-3-ol	64.26	0.55	0.42	HQ6	MOL000380
	Bifendate	31.1	-0.06	0.67	HQ7	MOL000387
	formononetin	69.67	0.02	0.21	HQ8	MOL000392
	(3R)-3-(2-hydroxy-3,4-dimethoxyphenyl)chroman-7-ol	67.67	0.34	0.26	HQ9	MOL000438
	1,7-Dihydroxy-3,9-dimethoxy pterocarpene	39.05	-0.04	0.48	HQ10	MOL000442
Baishu	α-Amyrin	39.51	1.28	0.76	BS1	MOL000028
	(3 S,8 S,9 S,10R,13R,14 S,17R)-10,13-dimethyl-17-[(2R,5 S)-5-propan-2-yloctan-2-yl]-2,3,4,7,8,9,11,12,14,15,16,17-dodecahydro-1 H-cyclopenta[a]phenanthren-3-ol	36.23	1.09	0.78	E1	MOL000033
	3β-acetoxyatractylone	54.07	1.08	0.22	BS2	MOL000049
	8β-ethoxy atractylenolide III	35.95	1.12	0.21	BS3	MOL000072
Haifengteng	(2R,3R,3aS)-3a-allyl-2-(1,3-benzodioxol-5-yl)-5-methoxy-3-methyl-2,3-dihydrobenzofuran-6-one	59.99	0.33	0.43	HFT1	MOL000308
	Denudatin B	61.47	0.35	0.38	HFT2	MOL000310
	futokadsurin C	61.09	0.78	0.45	HFT3	MOL000312
	Galgravin	57.12	0.48	0.39	HFT4	MOL000313
	(2 S,3 S,4 S,5 S)-2,5-bis(3,4-dimethoxyphenyl)-3,4-dimethyltetrahydrofuran	57.12	0.55	0.39	HFT5	MOL000314
	isofutoquinol A	59.2	0.31	0.48	HFT6	MOL000316
	Bicyclo(3.2.1)oct-3-ene-2,8-dione, 7-(4-hydroxy-3-methoxyphenyl)-5-methoxy-6-methyl-3-(2-propenyl)-, (1R-(6-endo,7-exo))-	94.67	-0.28	0.32	HFT7	MOL000319
	(2 S,3 S)-2-(3,4-dimethoxyphenyl)-7-methoxy-3-methyl-2,3-dihydrobenzofuran-5-carbaldehyde	42.15	0.63	0.32	HFT8	MOL000321
	Kadsurenone	54.72	0.52	0.38	HFT9	MOL000322
	Kadsurin A	56.83	0.17	0.5	HFT10	MOL000323
	kadsurin B	30.55	0.8	0.46	HFT11	MOL000324
	(4R)-2-allyl-4-[(E)-2-(4-hydroxy-3-methoxyphenyl)-1-methylvinyl]-4,5-dimethoxy-1-cyclohexa-2,5-dienone	55.14	0.22	0.3	HFT12	MOL000327
	piperkadsin B	55.44	0.21	0.41	HFT13	MOL000328
	piperlactam S	40.44	0.12	0.4	HFT14	MOL000330
	Stigmasterol	43.83	1	0.76	B1	MOL000449
	n-coumaroyltyramine	85.63	-0.28	0.2	HFT15	MOL000332
	wallichinine	61.64	0.3	0.33	HFT16	MOL000334
	futoquinol	59.83	0.32	0.36	HFT17	MOL000336
Shengdi	transp-hydroxy cinnamic acid methyl ester	Pharmacokinetics	Conditions		SD1	According to the chemical formula
	3-methoxy-4-hydroxyl cinnamic aldehyde		“GI absorption: High”		SD2	
	rehmapicrogenin		“BBB permeant: Yes”		SD3	
	3-indolecarboxylic acid		“Druglikenes: Yes”		SD4	

### The PPI network analysis

The target–gene data of the active ingredient of the drug and AD were derived, and the intersection gene of the drug and disease was screened by Venn mapping method. By using the STRING database, the network nodes analysis of the regulatory targets of interaction with AD was performed, and the PPI network was graphed, including 346 target protein nodes with a total of 7542 interaction lines (Fig. 2B). Targets AKT1, IL6, MAPK3, TP53, VEGFA, EGFR, and MAPK1 were predicted by the topological parameters of protein interactions combined with the pathways in the KEGG enrichment analysis, which were the prime objects of our next experiment (Table 2).

**Table 2:** Topological parameters of 14 major targets between ingredient targets from compound and AD significant targets

Uniprot ID	Symbol	AverageShortestPathLength	BetweennessCentrality	ClosenessCentrality	Degree
P31749	AKT1	1.40988372	0.05840201	0.70927835	203
P05231	IL6	1.46802326	0.05047889	0.68118812	186
P27361	MAPK3	1.47383721	0.03877163	0.67850099	181
P04637	TP53	1.53197674	0.02620906	0.65275142	169
P15692	VEGFA	1.52616279	0.02459147	0.6552381	167
P01375	TNF	1.54069767	0.02556992	0.6490566	164
P12931	SRC	1.55523256	0.02601209	0.64299065	154
P28482	MAPK1	1.58139535	0.01918835	0.63235294	145
P42574	CASP3	1.59302326	0.01900327	0.62773723	145
P10145	CXCL8	1.59883721	0.02352492	0.62545455	143
P00533	EGFR	1.59593023	0.01554633	0.62659381	142
P40763	STAT3	1.625	0.01193503	0.61538462	139
P05412	JUN	1.63081395	0.01189425	0.61319073	136
P45983	MAPK8	1.625	0.01209135	0.61538462	135

### GO analysis

**Fig. 3:**
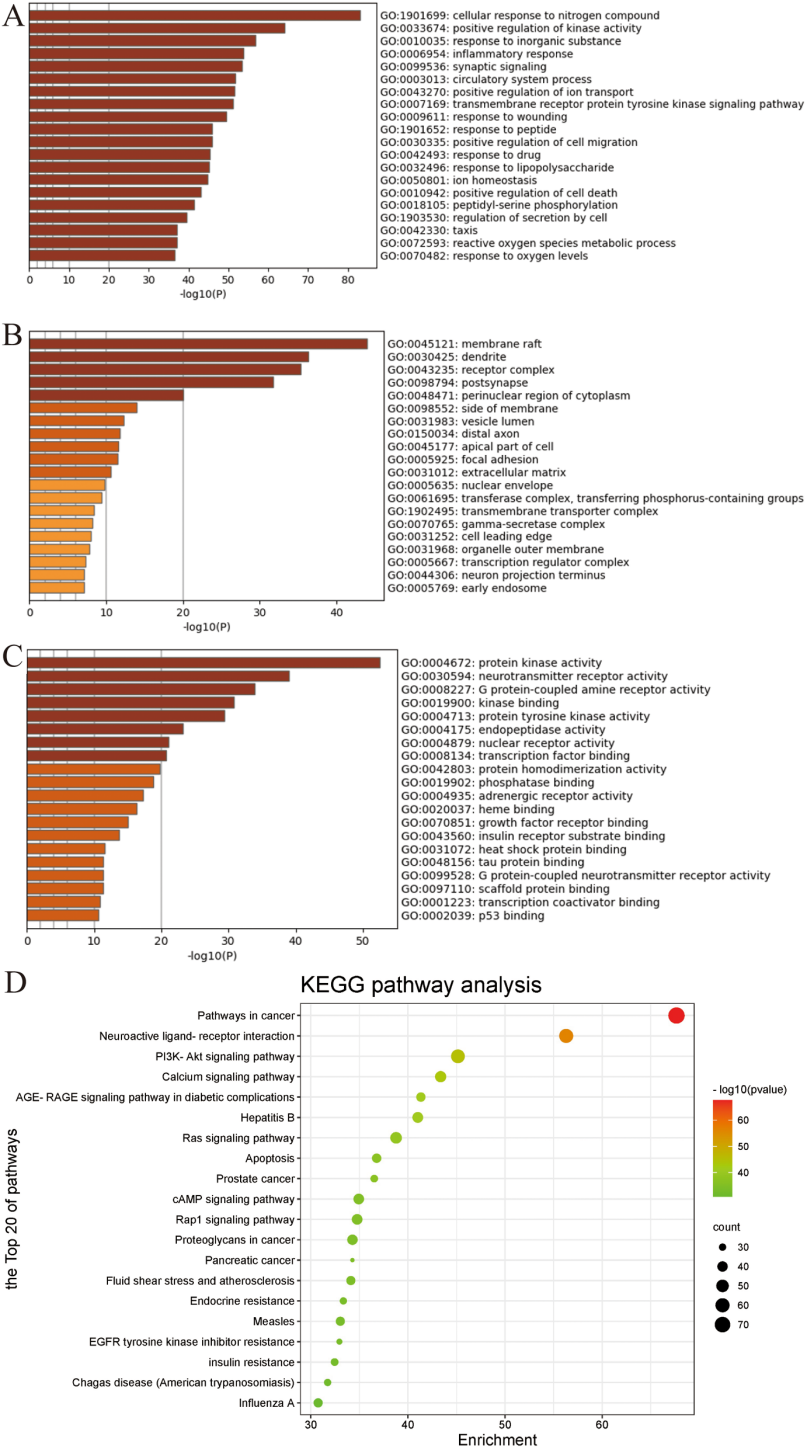
(**A**, **B**, **C**) The top 20 significantly enriched terms in BP (biological process) and MF (molecular function), CC (cellular component) categories. (**D**) The top 20 key signal pathway of KEGG enrichment analysis

Using the Metascape database for GO enrichment analysis, the top 20 entries with the highest correlation were obtained, including 20 biological process (BP) entries (Fig. 3A), 20 molecular function (MF) entries (Fig. 3B), and 20 cell composition (CC) entries (Fig. 3C). BSYJF was primarily involved in BPs involving regulation of biological quality, cellular response to synaptic signaling, ion homeostasis, positive regulation of cell death, ROS metabolic process, and response to oxygen levels. BSYJF affects AD by regulating MF, protein kinase activity, neurotransmitter receptor activity, kinase binding, protein tyrosine kinase activity, nuclear receptor activity, heme binding, heat shock protein binding, tau protein binding, G-protein-coupled neurotransmitter receptor activity, and p53 binding. Membrane raft, dendrite, receptor complex, transferring phosphorus-containing groups, gamma-secretase complex, neuron projection terminus, and early endosome are possibly related to BSYJF-treatment-targeted neural cells.

### KEGG pathway enrichment analysis

The KEGG signal pathway analysis was carried out by using Metascape database, and the first 20 key signal pathways were selected (Supplement 1 and Fig. 3D), mainly involved neuroactive ligand–receptor interaction, PI3K/AKT signaling pathway, calcium signaling pathway, Ras signaling pathway, apoptosis, cAMP signaling pathway, and Rap1 signaling pathway. These pathways are closely associated with anti-AD. Above the degree of targets, the 14 key target points of the highest value were AKT1, MAPK3, IL6, TP53, VEGFA, TNF, SRC, MAPK1, STAT3, EGFR, CXCL8, JUN, CASP3, and MAPK8, suggesting that these 14 targets may be critical in AD with BSYJF treatment.

### The interaction of target–signaling pathway–network

Our drug composition–disease target–signal pathway analysis results are depicted in Fig. 2C, including a total of 53 nodes, 263 overlapping targets of composition–disease, and 20 signal path nodes. The more edges of the node showed the higher description value, indicating that the node may be the key node for treating AD. These findings implied that the BSYJF components might have a strong potential to directly bind with AKT1, Bcl-2, and CASP3. The PI3K/Akt signaling pathway was ranked as the top three matched with targets of p-value. The supposition should be validated experimentally in further studies.

### Molecular docking and a mass spectrometry analysis

**Fig. 4:**
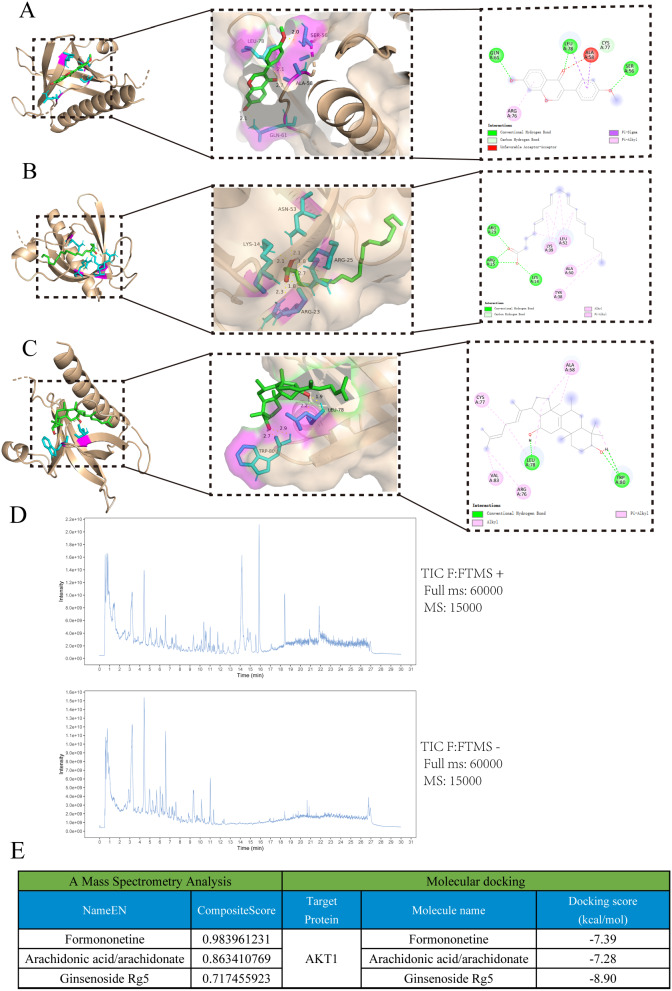
Molecular models of the binding of AKT1 with (**A**) Formononetin, (**B**) Arachidonate, and (**C**) Ginsenoside Rg5 shown as 3D and 2D diagrams. (**D**) The results of ESI-Q-Exactive-Orbitrap MS in positive/negative ion mode. (**E**) Molecular docking and a mass spectrometry analysis

The results of the PPI network analysis and enrichment analysis implied that the target of the AKT1 gene might have strong potential. The PI3K/AKT signaling pathway was ranked as the top three matched with targets of p-value. Therefore, AKT1 was selected for molecular docking with the chief ingredients of herbs in further experiments. The high binding affinity of several natural compounds with the target had been discovered. Three results among them with the most favorable free binding energy were selected by a mass spectrometry analysis, which was to determine the three constituents that existed in the extracts of BSYJF (Fig. 4D and Supplement 2). The protein AKT1 was molecularly docked with the active components (formononetin, arachidonate, and ginsenoside Rg5), with the highest docking scores in the molecular docking analysis. Figure 4A and C depict the best docking combinations for the target protein and components, including formononetin, arachidonate, and ginsenoside Rg5, with binding energies of − 7.39, − 7.28, and − 8.90 kcal/mol, respectively. These values showed that the target protein exhibited a good binding ability to these components (Fig. 4E).

## Effects of different concentrations of BSYJF on the expression of AKT and p-AKT proteins and the translocation of nuclear factor erythroid 2-related factor (Nrf2) to the nucleus

**Fig. 5:**
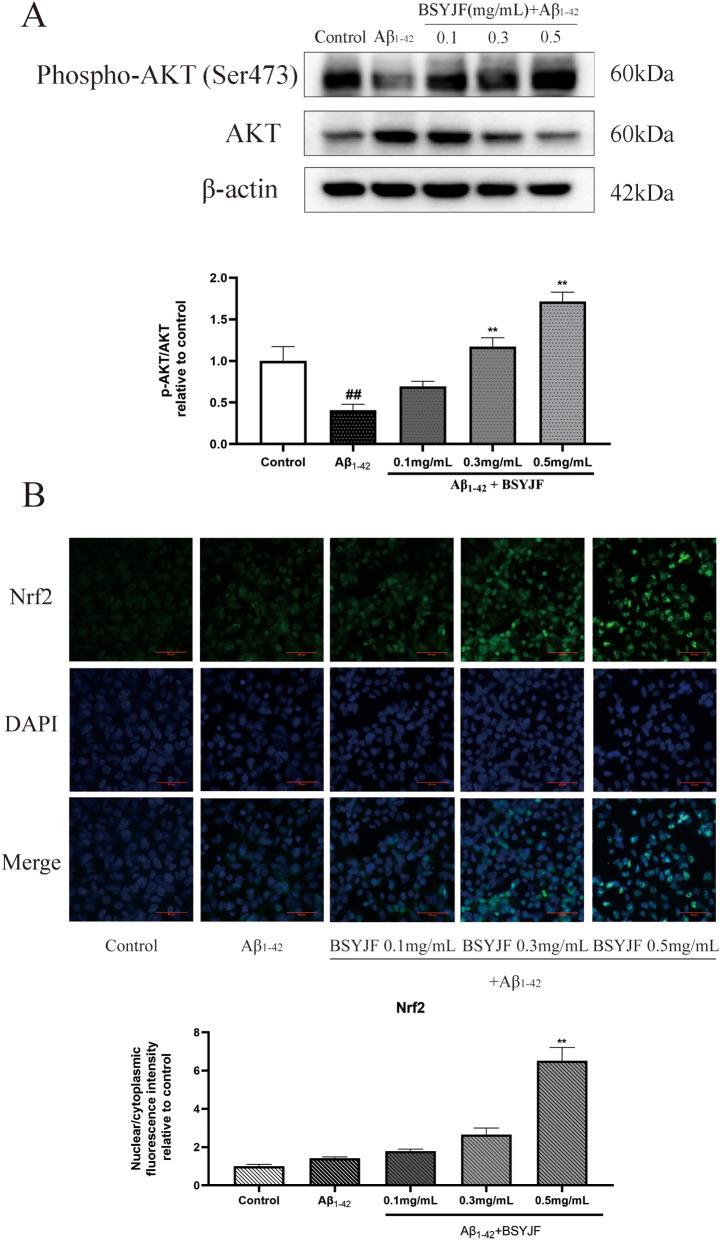
(**A**) Effects of BSYJF (0.1, 0.3, and 0.5 mg/mL) on the expression of the PI3K/AKT pathway proteins in Aβ_1–42_-stimulated SKNMC cells. Western blotting analysis revealed the expression levels of p-AKT and AKT. Actin levels were measured for the confirmation of an equal amount of protein loading. (**B**) Immunofluorescence staining demonstrated the nuclear translocation of Nrf2 in SKNMC cells with BSYJF (0.1, 0.3, and 0.5 mg/mL). Images were captured with the fluorescence microscope at a magnification of × 400. The fluorescence intensity was semi-quantified using ImageJ. Data are expressed as mean ± SEM. ^#^*p* < 0.05, ^##^*p* < 0.01 versus control. ^*^p < 0.05, ^**^p < 0.01 versus the group with Aβ_1–42_

To examine the effect of BSYJF on the PI3K/AKT/Nrf2 pathway, SKNMC cells were pretreated with different concentrations of BSYJF (0.1, 0.3, and 0.5 mg/mL) for 2 h and treated with Aβ_1–42_ (20 µM) for 24 h. The expression levels of p-AKT/AKT were determined by western blot, and Nrf2 was measured by immunofluorescence staining. As depicted in Fig. 5A, cells treated with Aβ_1–42_ significantly inhibited the level of p-AKT/AKT. Compared with the Aβ_1–42_-induced group, pretreatment with BSYJF significantly reversed the inhibitory effect of Aβ_1–42_ in a concentration-dependent manner. The SKNMC cells treated with Aβ_1–42_ displayed a slight increase in the translocation of Nrf2 to the nucleus compared with the control; however, there was no statistical significance. Compared with the model, the translocation of Nrf2 to the nucleus in the cells pretreated with BSYJF was increased in a concentration-dependent manner, and 0.5 mg/mL BSYJF displayed statistical significance (Fig. 5B). Therefore, our study showed that BSYJF could activate the PI3K/AKT/Nrf2 pathway in Aβ_1–42_-treated SKNMC cells in a concentration-dependent manner. A high concentration of BSYJF (0.5 mg/mL) demonstrated the best effect. Therefore, 0.5 mg/mL was selected for the follow-up experiments.

### Effects of BSYJF on the expression of PI3K/AKT/Nrf2 pathway-related proteins

**Fig. 6:**
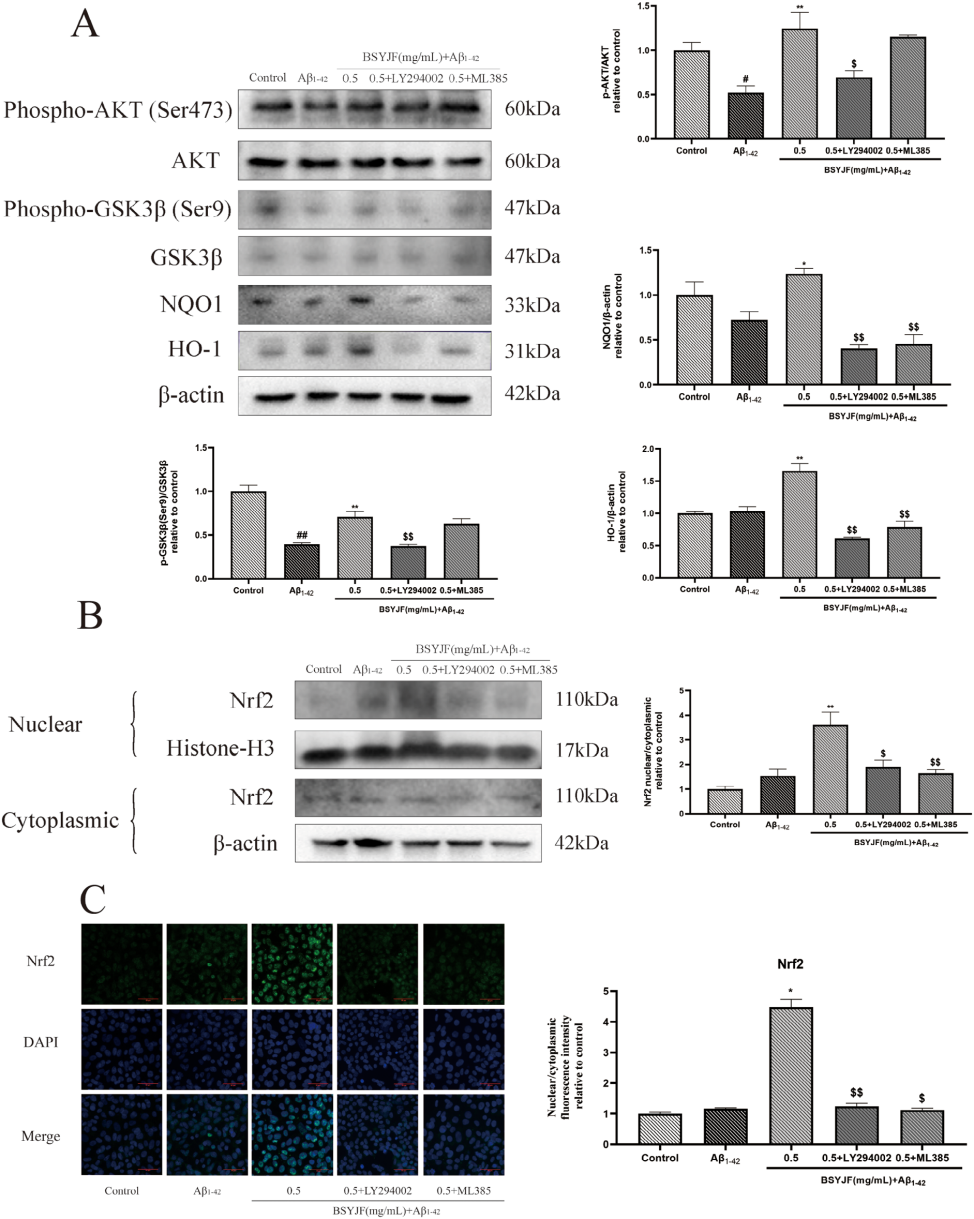
(**A**) Effects of the expression of PI3K/AKT/Nrf2 pathway proteins in Aβ_1–42_-stimulated SKNMC cells with or without BSYJF (0.5 mg/mL) or inhibitors. Western blotting analysis showed the expression levels of p-AKT, AKT, p-GSK3β, GSK3β, NQO1, and HO-1. Actin levels were measured for the confirmation of an equal amount of protein loading. (**B**) Effects of the expression of PI3K/AKT/Nrf2 pathway proteins in Aβ_1–42_-stimulated SKNMC cells with or without BSYJF (0.5 mg/mL) or inhibitors. Western blotting analysis showed the expression levels of Nrf-2. Actin levels were measured for the confirmation of an equal amount of protein loading. (**C**) Immunofluorescence staining revealed the nuclear translocation of Nrf2 in SKNMC cells with BSYJF (0.5 mg/mL). Images were captured with the fluorescence microscope at a magnification (×200). The fluorescence intensity was semi-quantified by ImageJ. Data are expressed as mean ± SEM. ^*^p < 0.05, ^**^p < 0.01 versus the group with Aβ_1–42_. ^$^*p* < 0.05, ^$$^*p* < 0.01 versus the group treated with BSYJF

The above results indicated that BSYJF might activate the PI3K/AKT/Nrf2 pathway in Aβ_1–42_-stimulated SKNMC cells. To further evaluate the effect of BSYJF through the PI3K/AKT/Nrf2 signaling pathway, SKNMC cells were pretreated with/without 0.5 mg/mL BSYJF or 20 µM LY294002 (PI3K/AKT inhibitor)/10 µM ML385 (Nrf2 inhibitor) for 2 h and subsequently stimulated with Aβ_1–42_ for 24 h. The expression levels of AKT, p-AKT (Ser473), GSK3β, p-GSK3β (Ser9), NQO1, and HO-1 were determined by western blotting. Our results showed that BSYJF could reverse the levels of p-AKT/AKT and p-GSK3β/GSK3β compared with the Aβ_1–42_-induced group, while LY294002 treatment attenuated this effect. Compared with the Aβ_1–42_ group, BSYJF obviously increased the expression of NQO1 and HO-1, while LY294002/ML385 restrained the expression of the two proteins (Fig. 6A). We observed Nrf2 nuclear translocation via western blotting and immunofluorescence staining. Compared with the model, BSYJF markedly enhanced the translocation of Nrf2 to the nucleus, whereas LY294002/ ML385 reversed the translocation (Fig. 6B-C). In summary, BSYJF could activate the pathay through the PI3K/AKT/Nrf2 pathway in Aβ_1–42_-stimulated SKNMC cells.

### Effects of BSYJF on the levels of ROS, CAT, and SOD activities

**Fig. 7:**
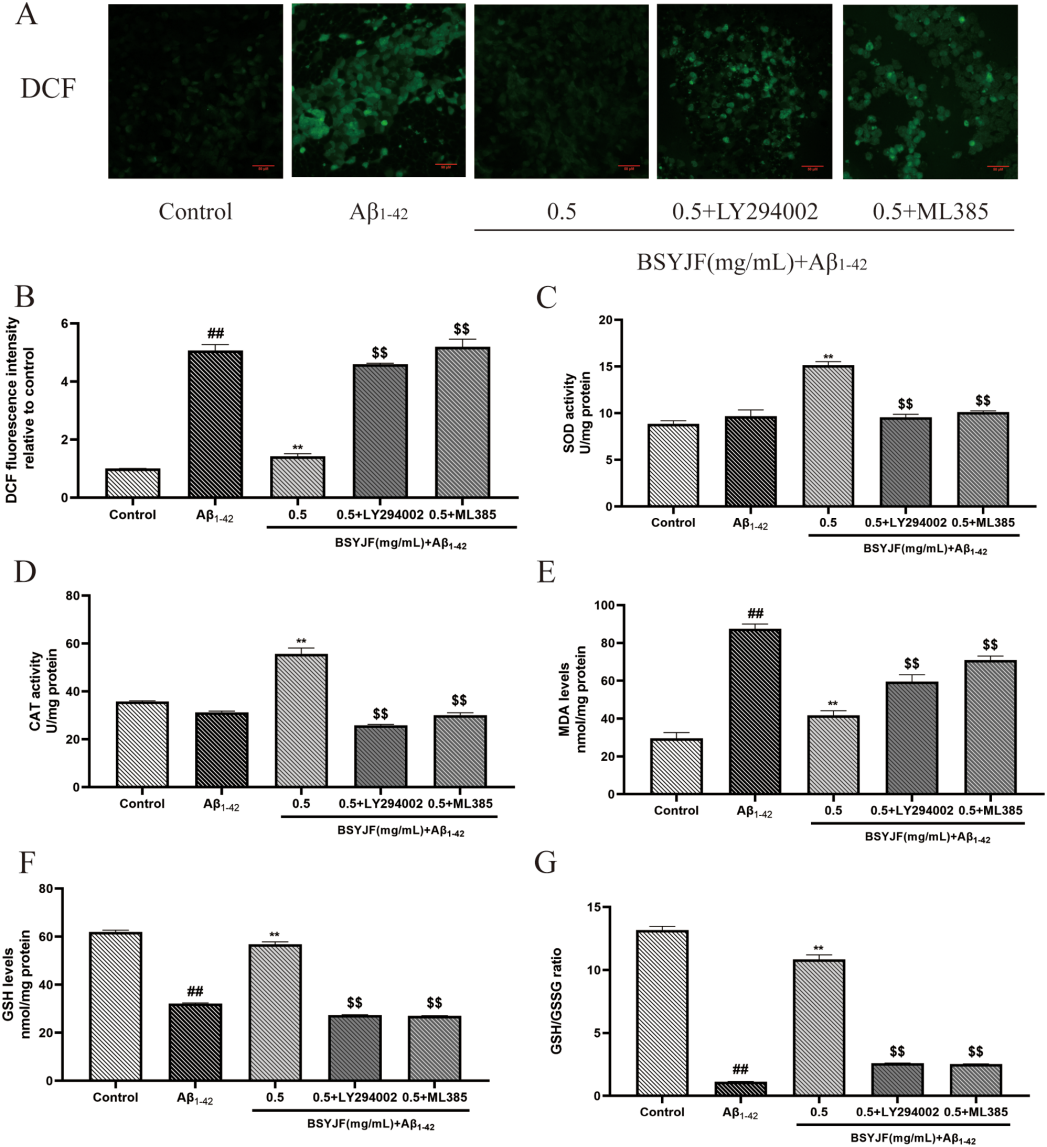
(**A**-**B**) Effects of BSYJF with or without LY294002/ML385 on ROS levels in Aβ_1–42_-induced SKNMC cells. Images were taken with a fluorescence microscope at a magnification (×200). The fluorescence intensity was calculated by ImageJ. (**C**) Effects of BSYJF with or without LY294002/ML385 on CAT levels in Aβ_1–42_-induced SKNMC cells. (**D**) Effects of BSYJF with or without LY294002/ML385 on SOD levels in Aβ_1–42_-induced SKNMC cells. (**E**) Effects of BSYJF with or without LY294002/ML385 on MDA levels in Aβ_1–42_-induced SKNMC cells. (**F**-**G**) Effects of BSYJF with or without LY294002/ML385 on levels of GSH and GSH/GSSG ratio in Aβ_1–42_-induced SKNMC cells. Data are expressed as mean ± SEM. ^##^*p* < 0.01, compared with the control; ^**^p < 0.01, compared with the Aβ_1–42_; ^$$^*p* < 0.01, compared with BSYJF treatment

The ROS levels in Aβ_1–42_-induced SKNMC cells were detected via DCFH-DA. The production of ROS discernibly increased in SKNMC cells treated with Aβ_1–42_. We found that BSYJF could reduce ROS levels, and LY294002/ML385 could suppress the effect of BSYJF (Fig. 7A-B). Compared with the model, BSYJF obviously increased the activity of CAT and total SOD, while LY294002/ML385 reversed the levels. Considering this result, the anti-oxidative effects of BSYJF on Aβ_1–42_-treated SKNMC cells were associated with the PI3K/AKT/Nrf2 signaling pathway possibly (Fig. 7C-D).

## BSYJF could attenuate ferroptosis in Aβ_1–42_-treated SKNMC cells by activating the PI3K/AKT/Nrf2 pathway

### Effects of BSYJF on cellular lipid peroxidation

**Fig. 8:**
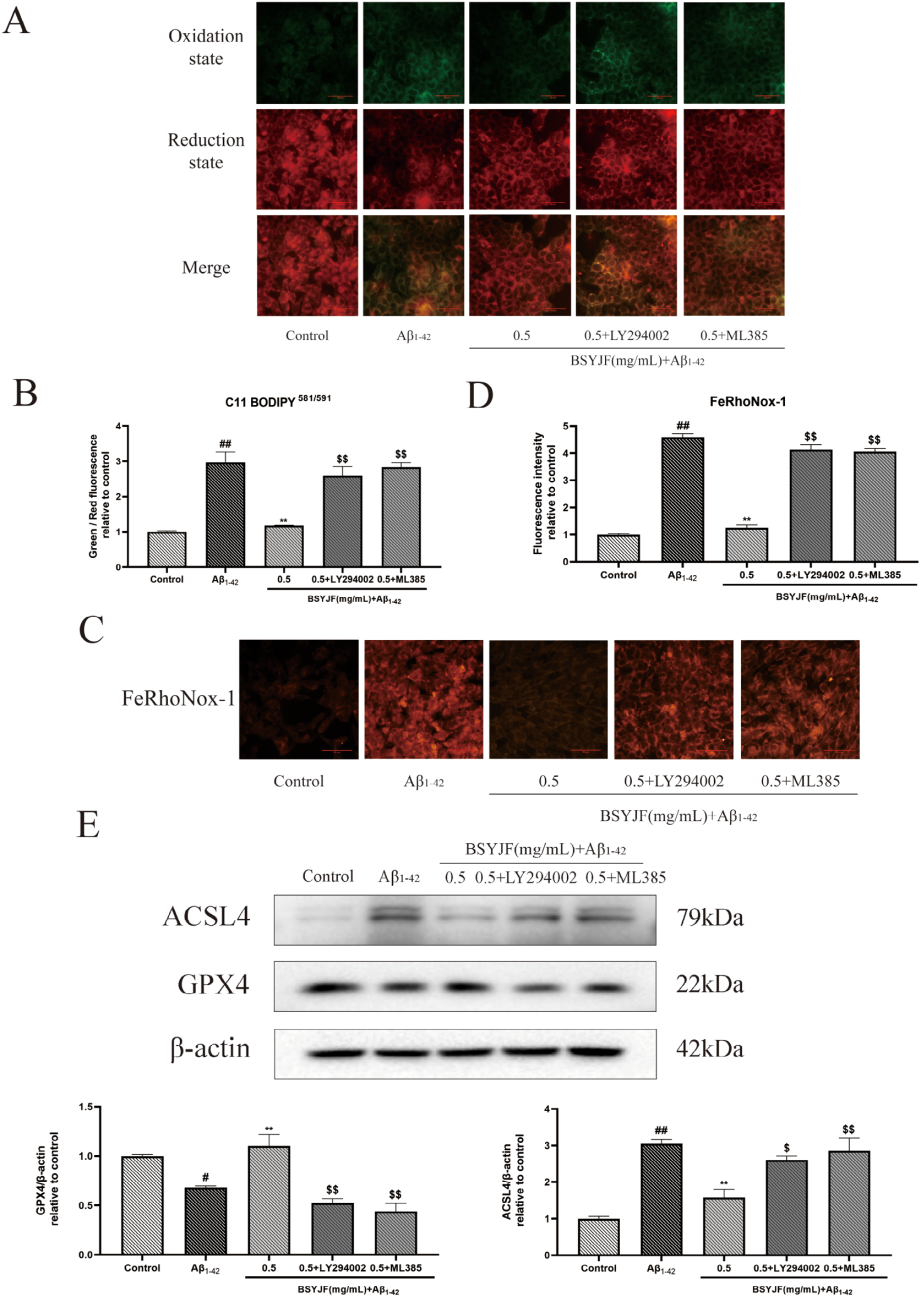
(**A**, **B**) The cellular lipid peroxidation was detected by the C11 Bodipy^581/591^ probe stained. The green fluorescence was oxidized state and red fluorescence was non-oxidized state. Images were taken with a fluorescence microscope (×400). The fluorescence intensity was semi-quantified by Image J. (**C**, **D**) The deposition of Fe^2+^ by FeRhonox-1 probe stained in Aβ_1−42_-treated SKNMC cells. Effects of BSYJF with or without LY294002/ML385 on Fe^2+^ levels in Aβ_1−42_-induced SKNMC cells. Images were taken with a fluorescence microscope. The fluorescence intensity was semi-quantified by Image J. (**E**) The protective mechanism of BSYJF on alleviating ferroptosis. Western blot analysis showed the levels of ACSL4 and GPX4 proteins in in Aβ_1−42_-stimulated SKNMC cells. Actin levels were measured for the confirmation of equal amount of protein loading. Data were expressed as mean ± SEM. ^#^*p* < 0.05, ^##^*p* < 0.01, compared with the control; ^**^p < 0.01, compared with the model; ^$^*p* < 0.05, ^$$^*p* < 0.01, compared with the BSYJF-treatment

To evaluate the effect of BSYJF on lipid peroxidation in Aβ_1–42_-treated SKNMC cells, we detected the levels of MDA and used C11 Bodipy^581/591^ staining. We noted that BSJYF could reduce the aggregation of the MDA in Aβ_1–42_-treated SKNMC cells, and inhibitors reversed the decrease of the MDA with BSYJF treatment (Fig. 7E). C11 Bodipy^581/591^ probe, a lipid-soluble fluorescent indicator of lipid oxidation, was used to measure cellular lipid peroxidation. After BSYJF treatment, the green fluorescence (oxidation state) was decreased, and red fluorescence (reduction state) was increased in Aβ_1–42_-treated SKNMC cells, while the inhibitors reversed this phenomenon. In other words, BSYJF could alleviate the lipid peroxidation of Aβ_1–42_-treated SKNMC cells (Fig. 8A-B).

### Effects of BSYJF on the level of GSH/GSSG

The expression levels of GSH and GSH/GSSG were explored in the next experiment. BSJYF increased GSH levels in Aβ_1–42_-treated SKNMC cells, and the inhibitors decreased the GSH levels compared with BSYJF treatment alone. After treatment with BSYJF, GSSG was reduced by glutathione reductase to yield a greater level of GSH. Specifically, the synthesis of GSSG was reduced to a certain extent. Treatment with BSYJF also increased the GSH/GSSG ratio when low levels of GSH were measured in model cells, and this may be ascribed to the stimulation of GSH synthesis. To sum up, GSH levels were improved by BSYJF treatment in a state of oxidative stress (Fig. 7F-G) [18].

### Effects of BSYJF on the aggregation of Fe^2+^ and the expression of ferroptosis-related proteins

Next, we detected the deposition of Fe^2+^ by FeRhonox-1 probe staining. Compared with control, Aβ_1–42_ could induce the deposition of Fe^2+^ in SKNMC cells. Fe^2+^ deposition was decreased upon treatment with BSYJF alone, while the inhibitors LY294002/ML385 increased Fe^2+^ deposition (Fig. 8C-D). Then, we evaluated the changes in ferroptosis-related protein expression in Aβ_1–42_-treated SKNMC cells. The expression of the protein ACSL4 increased, and the expression of the protein GPX4 decreased. After treatment with BSYJF, the increase in the level of protein ACSL4 and the decrease in the level of protein GPX4 were reversed. The protection was blocked by pretreatment with inhibitors (Fig. 8E). Considering the results, BSYJF could attenuate ferroptosis in Aβ_1–42_-treated SKNMC cells by activating the PI3K/AKT/Nrf2 pathway.

### Effects of BSYJF on the apoptosis of SKNMC cells by activating the PI3K/AKT/Nrf2 pathway

**Fig. 9:**
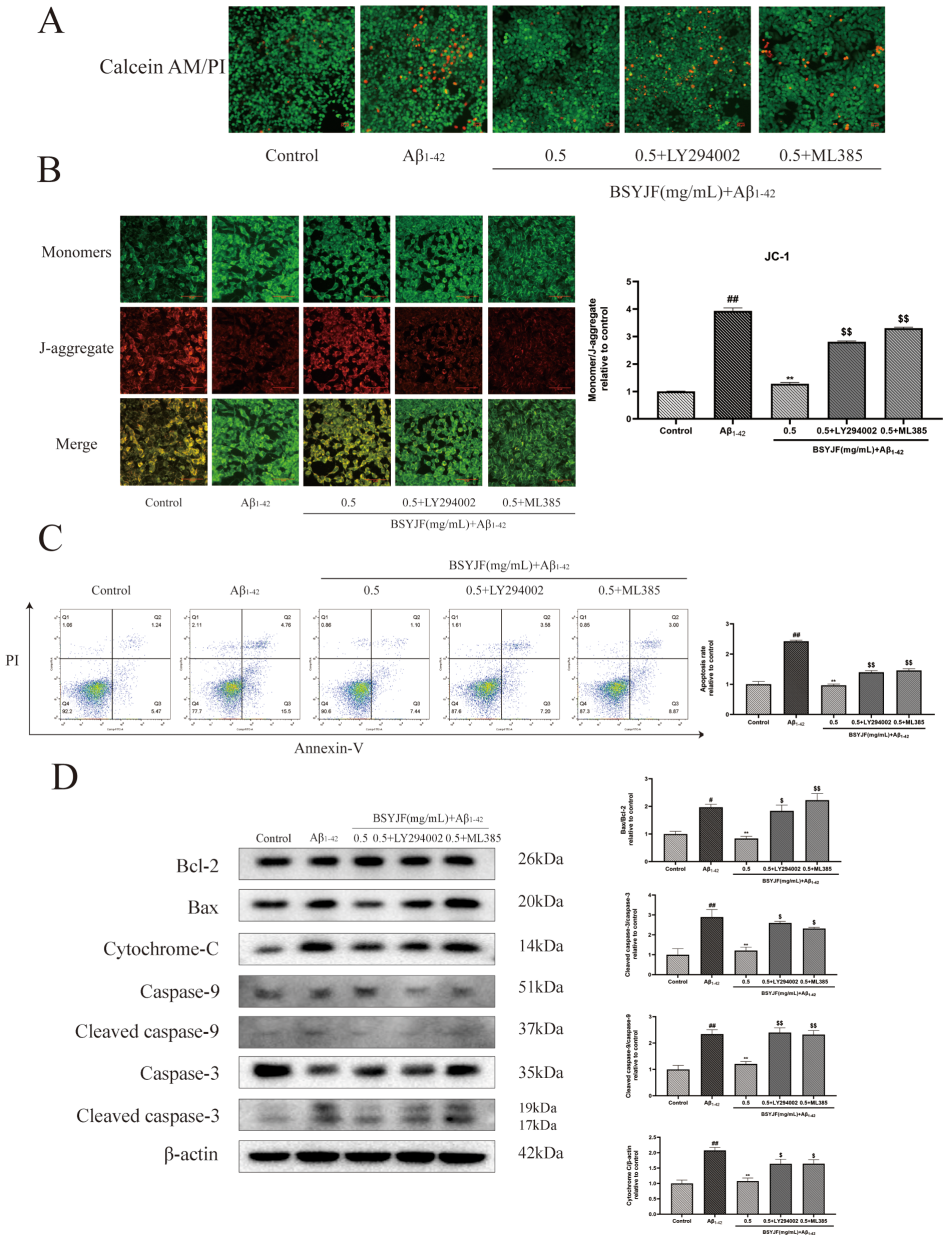
BSYJF prevented Antioxidant-mediated apoptosis in Aβ_1−42_-stimulated SKNMC cells. (**A**) Effect of BSYJF on cell viability with or without inhibitors LY294002/ML385 in Aβ_1−42_-induced SKNMC cells. Images were taken by an inverted fluorescence microscope at a magnification (×100). (**B**) Mitochondrial potential in Aβ_1−42_-stimulated SKNMC cells pretreated with or without BSYJF or inhibitors was taken by JC-1 staining. Red fluorescence represented the “J-aggregates” form and green represented the monomeric form of JC-1. Images were taken with a fluorescence microscope (×400). The fluorescence intensity was semi-quantified by Image J. A ratio of the green fluorescence and red fluorescence was analyzed. (**C**) The effect of BSYJF against apoptosis in Aβ_1−42_-stimulated SKNMC cells was detected by flow cytometric analysis. Cells were stained with Annexin V-fluorescein isothiocyanate/propidium iodide. The data were analyzed by Flow Jo. (**D**) Western blot analysis showed the levels of Bax/Bcl-2, cytochrome c, cleaved caspase-9/caspase-9 and cleaved caspase-3/caspase-3 proteins in in Aβ_1−42_-stimulated SKNMC cells with different treatments. Actin levels were measured for the confirmation of equal amount of protein loading. Data were expressed as mean ± SEM. ^#^*p* < 0.05, ^##^*p* < 0.01, compared with the control; ^**^p < 0.01, compared with the model; ^$^*p* < 0.05, ^$$^*p* < 0.01, compared with the BSYJF-treatment

We used calcein-AM/PI staining to evaluate the cell activity induced by Aβ_1–42_. The number of cells labeled with calcein-AM (live cells) increased, while those labeled with PI (dead cells) decreased upon treatment with BSYJF compared with cells treated with Aβ_1–42_ alone. Contrarily, a combination with LY294002/ML385 could increase the number of dead cells and reduce cell viability. The result demonstrated that BSYJF could alleviate Aβ_1–42_-induced oxidative injury in SKNMC cells (Fig. 9A). Next, we assessed the effect of BSYJF on the apoptosis of SKNMC cells treated with Aβ_1–42_ by JC-1 staining and flow cytometric analysis. The increase in green fluorescence intensity and the decrease in red fluorescence intensity indicated that cellular mitochondrial membrane potential (MMP) was reduced. The results showed that pretreatment with BSYJF could reverse the decline in MMP induced by Aβ_1–42_, and conversely, the two inhibitors dropped the MMP (Fig. 9B). Flow cytometric analysis indicated that pretreatment with BSYJF (0.5 mg/mL) decreased the apoptosis rates. However, the two inhibitors blocked the protection of BSYJF-pretreated cells (Fig. 9C). Next, we examined the effect of BSYJF on the expression of apoptosis-related proteins in Aβ_1–42_-treated SKNMC cells. The levels of Bax/Bcl-2, cytochrome-C, cleaved caspase-9/caspase-9, and cleaved caspase-3/caspase-3 were significantly increased in Aβ_1–42_-induced SKNMC cells. The expression of apoptosis-related proteins was downregulated upon treatment with BSYJF, while that of LY294002/ML385-treated cells was enhanced above proteins’ expression (Fig. 9D). We concluded that BSYJF could suppress apoptosis of SKNMC cells via the PI3K/AKT/Nrf2 pathway.

## Discussion

Network pharmacology is a systematic biological theory based on the composition analysis of large databases and the targets of large data on diseases, which explores the correlation between disease and drugs through a multi-layered analysis [19]. TCM is a complex chemical composition system. In this study, the therapies for AD were initially predicted by network pharmacology. By screening all 77 core components, the corresponding target genes of drugs and AD, which had a common total of 346 genes, were constructed to form the PPI network. The most valuable target genes were AKT1, MAPK3, IL6, TP53, VEGFA, TNF, SRC, MAPK1, STAT3, EGFR, CXCL8, JUN, CASP3, and MAPK8, suggesting that these 14 targets may be the key of AD with BSYJF treatment. KEGG pathway enrichment showed that the first 20 pathways with many enriched targets were neuroactive ligand–receptor interaction, PI3K-AKT signaling pathway, calcium signaling pathway, Ras signaling pathway, apoptosis, cAMP signaling pathway, and Rap1 signaling pathway. These signaling pathways are all related to the pathogenesis of AD, and nerve cells are the most basic structures in the nervous system, which are important functional units that transmit information and affect learning memory. The PI3K/AKT signaling pathway plays a vital role in the mechanism of AD [20]. The AKT gene was the key target with the highest value, which was linked to Aβ deposition and AD development [21]. The Akt family kinases consist of three different subtypes (AKT1, AKT2, and AKT3), which are involved in the PI3K/AKT pathway and play a central role in many cellular processes, including promoting cell proliferation, impairing apoptosis, and so on [22, 23]. Existing molecules have a favorable binding ability to the gene (AKT1) by a mass spectrometry analysis. According to the results of network pharmacology, we predicted the mechanisms of BSYJF on AD and verified that in our study.

The human brain is most susceptible to the effects of blood oxygen. Therefore, when the body is subjected to ischemia and hypoxia, the brain structure is the most vulnerable; specifically, neurons are the most sensitive to oxidative damage. Oxidative stress is a critical and inevitable process in AD pathogenesis [24–26]. Elevated oxidative stress markers lead to the deposition of Aβ plaques [27]. Aβ protein plays a destructive role in the brain, which in turn promotes the occurrence of oxidative stress [28]. Recently, many studies have demonstrated that increasing the activity of the PI3K/AKT pathway can exert anti-AD effects [20]. Oxidative stress is also regulated by various mechanisms, among which the PI3K/AKT pathway is a typical multifunctional signaling pathway related to cellular defense. GSK3β is a key component downstream of the PI3K/AKT pathway and is associated with Aβ deposition in AD. Inhibition of GSK3β activity was found to improve cognitive deficits and reduce oxidative stress response [29]. In another study, activation of the PI3K/AKT pathway could protect neuronal cells from Aβ-induced injury and apoptosis [30]. Glycogen synthase kinase 3 (GSK-3) is a downstream target of the PI3K/AKT signaling pathway, which is considered to play a key role in AD pathology such as tau phosphorylation, Aβ aggregation, memory deficit, neurogenesis, and synaptic dysfunction [31, 32]. GSK3β is one of the kinases of the GSK family, and its activity is related to AD progression, which can be regulated through the PI3K/AKT pathway [32]. Our study revealed that the expression of p-AKT/AKT was inhibited in Aβ_1–42_-induced SKNMC cells, while BSYJF could obviously enhance the p-AKT/AKT expression with increasing concentration. In addition, BSYJF combined with LY294002 was used to further explore its mechanism. LY294002 significantly inhibited the level of p-AKT/AKT and p-GSK3β/GSK3β compared with BSYJF treatment alone. Besides, GSK3β has been found to display an antioxidant effect by regulating Nrf2 [33], an important regulator under oxidative stress [34]. Triggering dissociation of Nrf2 from Keap1 or attenuating the effect of the Keap1-mediated protease on Nrf2 were impacted by oxidative stress and then translocated to the nucleus [35]. We found that BSYJF could elevate Nrf2 nuclear expression and promote nuclear translocation; however, LY294002 and ML385 reversed this effect. Consequently, activation of the PI3K/AKT pathway could inhibit the activity of GSK3β (phosphorylation at Ser9 indicates inhibition of activity) and affect the subsequent nuclear translocation of Nrf2. The results indicated that BSYJF could generate this effect via the PI3K/AKT/Nrf2 pathway.

The Nrf2/ARE pathway is involved in the regulation of oxidative stress, maintenance of the redox state, and regulation of antioxidant reaction elements. The protective effect against oxidative damage has also been widely studied in neurodegenerative diseases [36–38]. ROS, lipid oxidation, and glutathione are metabolites of oxidative stress [39, 40]. Nrf2 translocated to nuclear and bounded with ARE, which triggered transcription of downstream target genes, including HO-1, NQO1, and other antioxidant enzymes and proteins, such as superoxide dismutase and GSH-Px [41, 42]. The expression of downstream target genes plays a crucial role in maintaining redox homeostasis [43]. In Aβ_1–42_-stimulated SKNMC cells, LY294002 and ML385 inhibited the expressions of HO-1 and NQO1, while the above protein expressions were enhanced with BSYJF treatment. ROS accumulation can result in cellular injury, which affects the antioxidant defense systems in cells [44]. CAT is one of the crucial non-protein antioxidants in cells, scavenging the lipid peroxide free radicals [45], while SOD generates physiological activities, including antioxidant effects [46]. Our results demonstrated that BSYJF could eliminate ROS production and elevate the activity of CAT and SOD in Aβ1–42-treated SKNMC cells, while LY294002 and ML385 treatment inhibited the antioxidant capacity. Particularly, BSYJF could exert antioxidant effects via the Nrf2/ARE signaling pathway.

The metabolism of metal ions is also a way of contributing to AD progression [47]. Oxidative stress is also an important way to maintain iron metabolism [48]. Iron death is a mechanism leading to cell-dependent cell death and has been widely used in investigations on AD in recent years [49]. Lipid peroxidation is the driving force regulating iron death, and GPX4 is a key regulator of iron death [50, 51]. The imbalance of the antioxidant system of the nervous system can lead to the death of nerve cells. In this study, we verified the effects of BSYJF on apoptosis and ferroptosis. Ferroptosis is an iron-dependent, lipid peroxide-mediated cell death characterized by lipid peroxidation, Fe^2+^ accumulation, and GPX4 inactivation [52, 53]. Nrf2 was a transcriptional defense mechanism in ferroptosis [52]. The Nrf2/ARE pathway may be an appropriate strategy for regulating ferroptosis [54]. The deposition of Fe^2+^ assessed by FeRhonox-1 probe was decreased after Aβ_1–42_-induced SKNMC cells with BSYJF pretreatment. The inhibitors LY294002/ML385 conversely enhanced Fe^2+^ deposition. Fe^2+^, redox-active iron, could increase the production of ROS, leading to an increased production of lipid peroxides [55]. MDA—the end product of lipid peroxidation—was used to detect the extent of lipid peroxidation [45]. The expression level of MDA can reflect oxidative damage [56]. C11 Bodipy^581/591^ probe staining also indicated cellular lipid peroxidation. After BSYJF treatment, the MDA production and the green fluorescence (oxidized state) intensity decreased in Aβ_1–42_-treated SKNMC cells. The two inhibitors reversed the phenomenon. BSYJF could alleviate the lipid peroxidation in Aβ_1–42_-treated SKNMC cells. GSH could protect cells from oxidative damage [56]. GSH is an important antioxidant in cells, which protects cells from the damage of ROS and lipid peroxidation. Depletion of cellular GSH can lead to the weakened antioxidant capacity of cells, which is related to ferroptosis [57]. GPX4 protected cells from the detrimental effects of lipid peroxides, and ACSL4 was an essential component for ferroptosis execution [51, 58]. In Aβ_1–42_-induced SKNMC cells, BSYJF could improve GSH levels and GSH/GSSG ratio, but the two inhibitors were not. The protein expression of ACSL4 increased and that of GPX4 decreased when reversed-treated with BSYJF compared with Aβ_1–42_. The protection was blocked by pretreatment with inhibitors. Considering the results, we conclude that BSYJF could attenuate ferroptosis in Aβ_1–42_-treated SKNMC cells by activating the PI3K/AKT/Nrf2 pathway.

Caspases, which regulate apoptosis, are central components of the machinery [59]. Bax and Bcl-2 are also crucial downstream factors of the PI3K/AKT pathway. The PI3K/AKT pathway is also a major regulator of neuronal survival, which inhibits the apoptotic activity of GSK-3 and increases the level of Bcl-2 to block the neuronal apoptotic pathway [60, 61]. Our study disclosed that the apoptosis-related protein levels of Bax/Bcl-2, cytochrome-C, cleaved caspase-9/caspase-9, and cleaved caspase-3/caspase-3 were significantly decreased in Aβ_1–42_-cultured SKNMC cells with BSYJF pretreatment. The loss of integrity of the outer mitochondrial membrane was caused by proapoptotic members of the Bcl-2 family [44]. The Bax and Bcl-2 expression as well as ROS production were linked to changes in MMP [62]. The decline in MMP was a universal feature of cell apoptosis [63]. Pretreatment with BSYJF could reverse the repression of MMP in Aβ_1–42_-cultured SKNMC cells, and inhibitors were not. Flow cytometry analysis indicated that BSYJF could decrease the apoptosis rates compared with Aβ_1–42_ treatment, and the two inhibitors blocked the protection of cells with BSYJF pretreatment. BSYJF could protect cells against apoptosis, and this effect was mediated by the activation of PI3K/AKT/Nrf2 pathway.

**Fig. 10:**
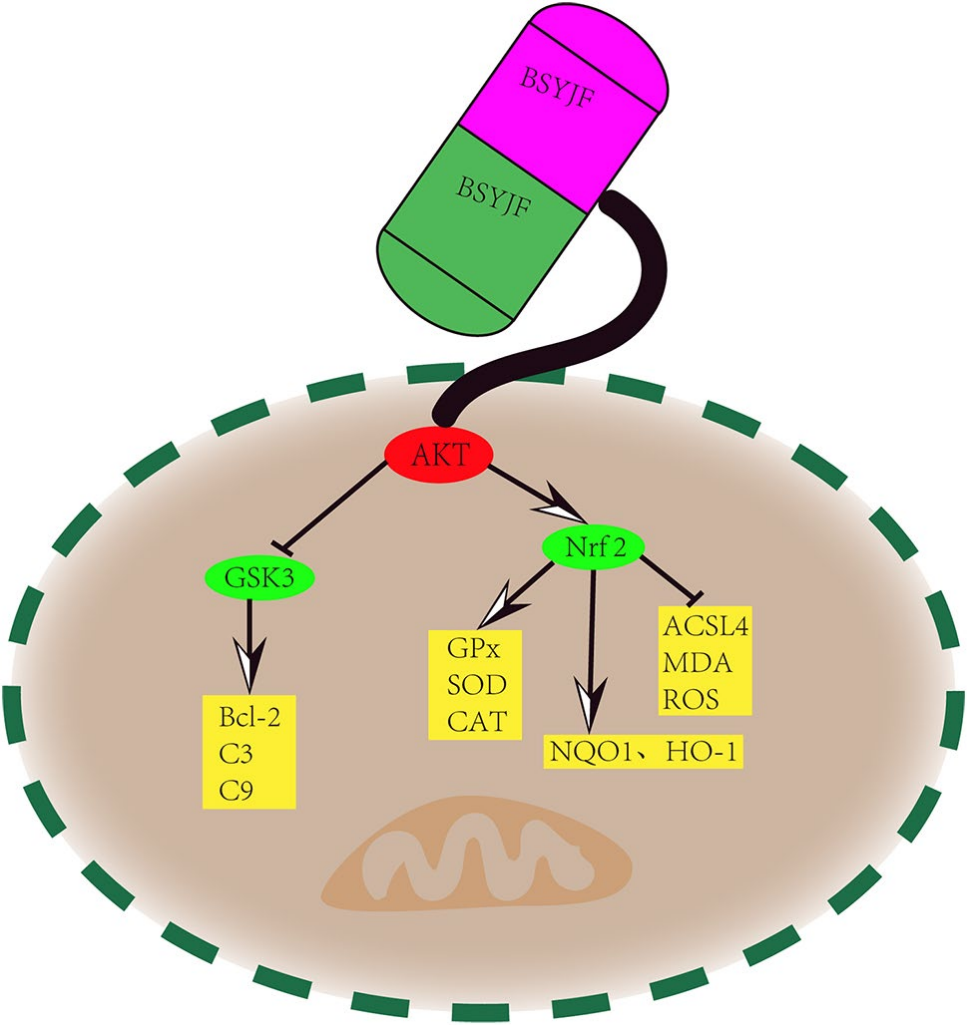
The BSYJF potential mechanism in the signal pathway

In Aβ_1–42_-treated SKNMC cells, we found that BSYJF could reduce cell apoptosis and attenuate ferroptosis. These experiments demonstrated that BSYJF plays an antioxidant effect, and this effect was mediated by the PI3K/AKT/Nrf2 pathway(Fig. 10). Our results indicated that in vitro experiments combined with network pharmacology effectively clarified that BSYJF could exert a protective effect in AD through the PI3K/AKT/Nrf2 signaling pathway.

For the current research, based on the multiple pathogenic mechanisms of AD and the urgent need for Chinese medicine screening for multi-target effects, it is necessary to establish a multi-biological model of the disease in addition to a multi-target efficacy evaluation system. Furthermore, in the future, we need to adjust the compatibility ratio of BSYJF to enhance its efficacy through the screening of a multidimensional efficacy evaluation system on AD.

## Conclusion

We conclude that KEGG pathway enrichment screened out the PI3K/AKT signaling pathway. Experimental validation proved that BSYJF treatment markedly increased the activation of PI3K/AKT pathway, inhibited the activity of GSK3β, promoted the nuclear translocation of Nrf2, and then attenuated cell apoptosis and ferroptosis in Aβ_1–42_-stimulated SKNMC cells. Our data provided compelling evidence that the protective effects of BSYJF might be linked to the regulation of PI3K/AKT/Nrf2 signal pathway, which can exert an anti-AD effect. Our study provides more options and evidence for AD treatment. However, only this part of the experiment is not enough, we needs further verification in *vivo* and in *vitro* experiments, so as to explore the value application of the compound in combination with clinical studies.

### Electronic supplementary material

Below is the link to the electronic supplementary material.

Supplementary Material 1

Supplementary Material 2

Supplementary Material 3

## Data Availability

The datasets generated or analyzed during the current study available from the corresponding author on reasonable request.

## Abbreviations

BSYJ: Bu-Shen-Yi-Jing-Fang
AD: Alzheimer’s disease
TTD: Therapeutic Target Database
TCMSP: Traditional Chinese Medicine System Pharmacology Database and Analysis Platform
GO: Gene ontology
KEGG: Kyoto Encyclopedia of Genes and Genomes
OB: Oral bioavailability
DL: Drug-like-ness
RCSB: Research Collaboratory for Structural Bioinformatics
PDB: Protein Data Bank
ROS: Reactive Oxygen Species
MDA: Lipid Peroxidation
CAT: Catalase
SOD: Total Superoxide Dismutase
