# Supplementary material for: Experimental verification about treatment of Bu-Shen-Yi-Jing-Fang in Alzheimer’s disease by the analysis of the feasible signaling pathway of network pharmacology
Source: BMC Complement Med Ther. 2024 Jun 8;24:222. doi: 10.1186/s12906-024-04527-w (PMC11162075; doi:10.1186/s12906-024-04527-w)
Supplement: Supplementary file 1 — Supplementary Material 1 [file 12906_2024_4527_MOESM1_ESM.docx]

**UHPLC-QE-MS: Non-target metabolomics detection report of traditional Chinese medicine**


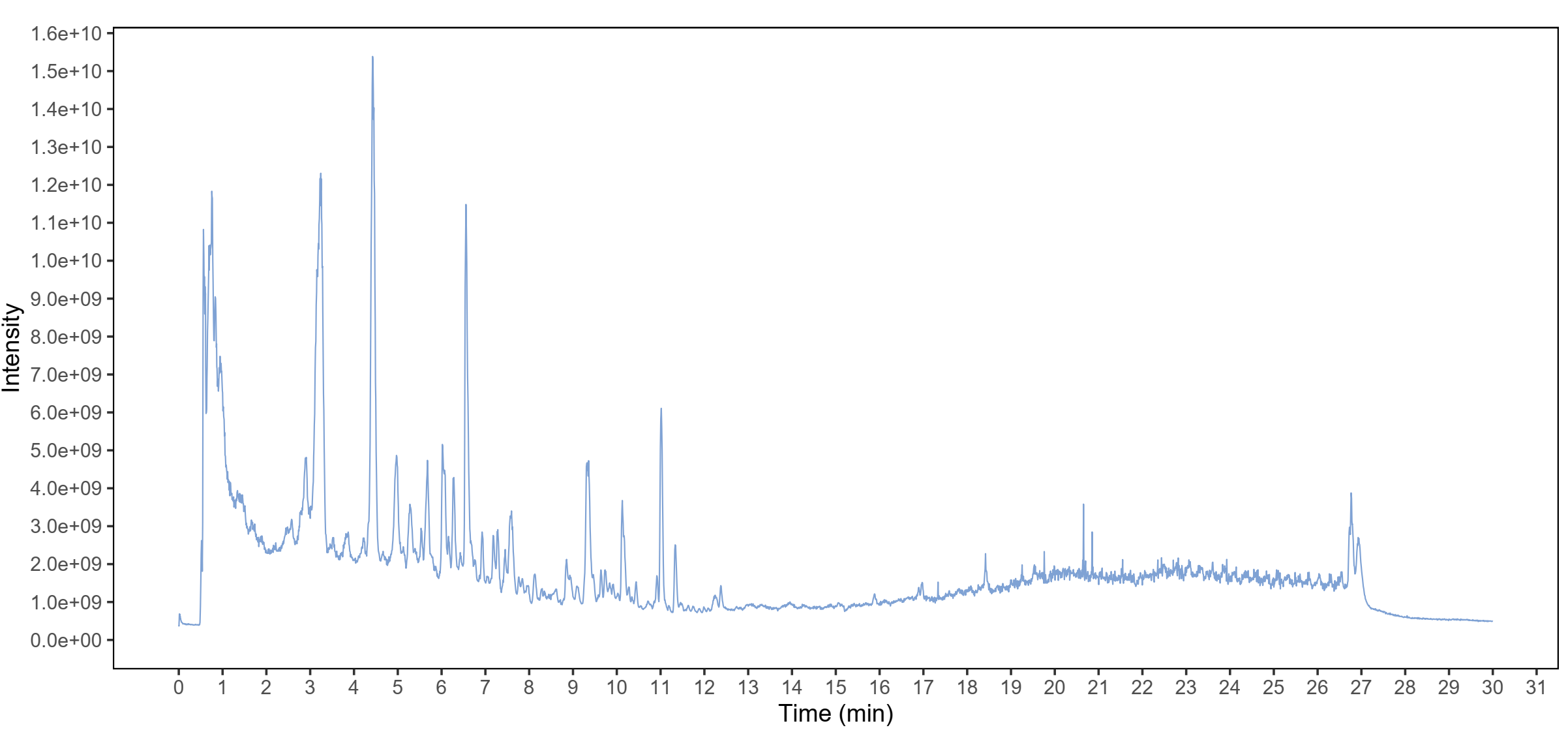


Fig1-1 NEG-T

Table 1-1 NEG-final

| **NameEN** | **CompositeScore** | **InChIKey** | **Formula** | **name** |
| --- | --- | --- | --- | --- |
| 5-OXO-D-PROLINE | 1 | ODHCTXKNWHHXJC-UHFFFAOYSA-N | C5H7NO3 | M128.035T266.768 |
| Coumaric acid | 1 | NGSWKAQJJWESNS-UHFFFAOYSA-N | C9H8O3 | M163.040T284.285 |
| Irigenin | 1 | TUGWPJJTQNLKCL-UHFFFAOYSA-N | C18H16O8 | M359.077T403.459 |
| Neohesperidin | 1 | ARGKVCXINMKCAZ-UHFFFAOYSA-N | C28H34O15 | M609.181T491.690 |
| p-Hydroxybenzaldehyde | 1 | RGHHSNMVTDWUBI-UHFFFAOYSA-N | C7H6O2 | M121.029T224.532 |
| Wogonin | 1 | XLTFNNCXVBYBSX-UHFFFAOYSA-N | C16H12O5 | M283.060T456.862 |
| Pyrogallol | 0.999118462 | WQGWDDDVZFFDIG-UHFFFAOYSA-N | C6H6O3 | M125.024T1617.600 |
| Sumaresinolic acid | 0.998933462 | KLHSKTMVSOWVLD-UHFFFAOYSA-N | C30H48O4 | M471.347T758.703 |
| 4-AMINOBENZOATE | 0.998788462 | ALYNCZNDIQEVRV-UHFFFAOYSA-N | C7H7NO2 | M136.040T331.378 |
| Cellobiose | 0.998322615 | GUBGYTABKSRVRQ-QRZGKKJRSA-N | C12H22O11 | M341.108T39.872 |
| Kaempferol | 0.997891615 | IYRMWMYZSQPJKC-UHFFFAOYSA-N | C15H10O6 | M285.040T450.315 |
| Asiatic acid | 0.997457077 | JXSVIVRDWWRQRT-UYDOISQJSA-N | C30H48O5 | M487.341T654.840 |
| C12-AS (TENTATIVE) | 0.995512 | MOTZDAYCYVMXPC-UHFFFAOYSA-N | C12H26O4S | M265.147T734.107 |
| 1,7-bis(4-hydroxyphenyl)heptan-3-one | 0.995089846 | QUHYUSAHBDACNG-UHFFFAOYSA-N | C19H22O3 | M297.153T735.899 |
| Scutellarein | 0.994718923 | JVXZRQGOGOXCEC-UHFFFAOYSA-N | C15H10O6 | M285.040T509.086 |
| Genkwanin | 0.994120846 | JPMYFOBNRRGFNO-UHFFFAOYSA-N | C16H12O5 | M283.060T629.380 |
| Ginsenoside Ro | 0.993805462 | NFZYDZXHKFHPGA-UHFFFAOYNA-N | C48H76O19 | M955.491T743.028 |
| Isorhamnetin | 0.993458692 | IZQSVPBOUDKVDZ-UHFFFAOYSA-N | C16H12O7 | M315.050T521.709 |
| Taurochenodeoxycholic Acid | 0.992257846 | BHTRKEVKTKCXOH-BJLOMENOSA-N | C26H45NO6S | M498.289T583.860 |
| 6-Gingerol | 0.991353769 | NLDDIKRKFXEWBK-AWEZNQCLSA-N | C17H26O4 | M293.178T838.326 |
| Nicotinic acid | 0.991162462 | PVNIIMVLHYAWGP-UHFFFAOYSA-N | C6H5NO2 | M122.025T1719.770 |
| Quinic acid | 0.990523 | AAWZDTNXLSGCEK-LNVDRNJUSA-N | C7H12O6 | M191.056T759.506 |
| Chrysin | 0.988957923 | RTIXKCRFFJGDFG-UHFFFAOYSA-N | C15H10O4 | M253.050T611.095 |
| Methyl hexadecanoate | 0.988355769 | FLIACVVOZYBSBS-UHFFFAOYSA-N | C17H34O2 | M315.253T712.871 |
| Ellagic acid | 0.987015077 | AFSDNFLWKVMVRB-UHFFFAOYSA-N | C14H6O8 | M300.998T326.048 |
| Citric acid | 0.986610769 | KRKNYBCHXYNGOX-UHFFFAOYSA-N | C6H8O7 | M191.019T49.895 |
| Formononetine | 0.983961231 | HKQYGTCOTHHOMP-UHFFFAOYSA-N | C16H12O4 | M267.066T469.804 |
| Kaempferol-3-O-glucoside | 0.983193308 | JPUKWEQWGBDDQB-UHFFFAOYNA-N | C21H20O11 | M447.093T375.782 |
| Aloeemodin | 0.982949462 | YDQWDHRMZQUTBA-UHFFFAOYSA-N | C15H10O5 | M269.045T542.862 |
| Gallic acid | 0.981096923 | LNTHITQWFMADLM-UHFFFAOYSA-N | C7H6O5 | M169.014T59.712 |
| Protocatechualdehyde | 0.979626077 | IBGBGRVKPALMCQ-UHFFFAOYSA-N | C7H6O3 | M137.024T160.164 |
| (E)-5-(2,3-dimethyl-4,5,6,7-tetrahydro-1H-tricyclo[2.2.1.02,6]heptan-3-yl)-2-methylpent-2-enoic acid | 0.978809692 | NZSCHTYUGUVLHG-WEVVVXLNSA-N | C15H22O2 | M233.154T761.485 |
| Salicylic acid | 0.976865538 | YGSDEFSMJLZEOE-UHFFFAOYSA-N | C7H6O3 | M137.024T385.599 |
| Catechol | 0.975877769 | YCIMNLLNPGFGHC-UHFFFAOYSA-N | C6H6O2 | M109.029T104.527 |
| Abscisic acid | 0.969448154 | JLIDBLDQVAYHNE-UHFFFAOYSA-N | C15H20O4 | M263.129T421.511 |
| Astragaloside II | 0.968103615 | AYWNHWGQTMCQIV-PENCHUSISA-N | C43H70O15 | M871.465T607.571 |
| [(2S,3R,4S,5S,6R)-6-[[(2R,3R,4R,5S,6R)-3,4-dihydroxy-6-(hydroxymethyl)-5-[(2S,3R,4R,5R,6S)-3,4,5-trihydroxy-6-methyloxan-2-yl]oxyoxan-2-yl]oxymethyl]-3,4,5-trihydroxyoxan-2-yl] (1S,2R,4aS,6aS,6bR,9R,10R,11R,12aR)-10,11-dihydroxy-9-(hydroxymethyl)-1,2,6a,6b,9,12a-hexamethyl-2,3,4,5,6,6a,7,8,8a,10,11,12,13,14b-tetradecahydro-1H-picene-4a-carboxylate | 0.965448231 | WYQVAPGDARQUBT-ZKFJACKJSA-N | C48H78O19 | M957.507T698.742 |
| Biochanin A | 0.963344154 | WUADCCWRTIWANL-UHFFFAOYSA-N | C16H12O5 | M283.060T519.439 |
| Genistein | 0.960456077 | TZBJGXHYKVUXJN-UHFFFAOYSA-N | C15H10O5 | M269.045T686.964 |
| Astragaloside III | 0.960274923 | FVFSMBDVZVUETN-BQAOMNQWSA-N | C41H68O14 | M783.450T584.304 |
| Soybean saponin fraction B1 | 0.960023615 | PTDAHAWQAGSZDD-UHFFFAOYNA-N | C48H78O18 | M941.511T616.587 |
| 5,7-dihydroxy-2-(4-hydroxyphenyl)-6,8-bis[3,4,5-trihydroxy-6-(hydroxymethyl)oxan-2-yl]chromen-4-one | 0.959584615 | FIAAVMJLAGNUKW-UHFFFAOYSA-N | C27H30O15 | M593.149T264.695 |
| Soyasapogenol E base + O-HexA-Hex-dHex | 0.958780923 | CROUPKILZUPLQA-UHFFFAOYSA-N | C48H76O18 | M939.495T650.910 |
| FA 18:1+3O | 0.955715154 | MDIUMSLCYIJBQC-UHFFFAOYSA-N | C18H34O5 | M329.233T602.607 |
| 3-[(Carboxycarbonyl)amino]-L-alanine | 0.949655769 | NEEQFPMRODQIKX-REOHCLBHSA-N | C5H8N2O5 | M174.956T1791.465 |
| alpha-Hederin | 0.947669923 | KEOITPILCOILGM-UHFFFAOYNA-N | C41H66O12 | M795.454T630.512 |
| Lamiide | 0.944143615 | VFYACENSDOLJGQ-SNONCDODSA-N | C17H26O12 | M421.133T142.040 |
| Glutamylphenylalanine | 0.944012308 | XHHOHZPNYFQJKL-UHFFFAOYSA-N | C14H18N2O5 | M293.124T197.260 |
| 1-Methoxy-3-carbaldehyde | 0.943322615 | NFGIENSPALNOON-UHFFFAOYSA-N | C10H9NO2 | M174.055T85.994 |
| columbianetin | 0.942338462 | YRAQEMCYCSSHJG-UHFFFAOYSA-N | C14H14O4 | M245.081T688.046 |
| N-Acetyl-DL-glutamic acid | 0.939383462 | RFMMMVDNIPUKGG-UHFFFAOYSA-N | C7H11NO5 | M188.056T51.496 |
| Geniposidic acid | 0.932451615 | ZJDOESGVOWAULF-OGJQONSISA-N | C16H22O10 | M373.112T101.056 |
| geniposide | 0.932168615 | IBFYXTRXDNAPMM-BVTMAQQCSA-N | C17H24O10 | M387.128T225.582 |
| Ethyl gallate | 0.932004769 | VFPFQHQNJCMNBZ-UHFFFAOYSA-N | C9H10O5 | M197.045T302.171 |
| DGMG 18:3 | 0.931462154 | MPSGDHOYFIUPSO-UHFFFAOYSA-N | C33H56O14 | M721.366T706.107 |
| Maleic acid | 0.929722462 | VZCYOOQTPOCHFL-UPHRSURJSA-N | C4H4O4 | M115.003T44.384 |
| Quercetin | 0.926444462 | REFJWTPEDVJJIY-UHFFFAOYSA-N | C15H10O7 | M301.035T509.086 |
| rhodioloside | 0.924238154 | ILRCGYURZSFMEG-RKQHYHRCSA-N | C14H20O7 | M299.113T152.863 |
| Psicose | 0.922876538 | BJHIKXHVCXFQLS-PUFIMZNGSA-N | C6H12O6 | M179.056T67.265 |
| Glyceric acid | 0.920541692 | RBNPOMFGQQGHHO-UHFFFAOYSA-N | C3H6O4 | M105.019T266.471 |
| Syringic acid | 0.917107846 | JMSVCTWVEWCHDZ-UHFFFAOYSA-N | C9H10O5 | M197.045T235.561 |
| Quercetin-3-O-glucoside | 0.915217385 | OVSQVDMCBVZWGM-UHFFFAOYSA-N | C21H20O12 | M463.086T340.659 |
| Methyl gallate | 0.914187154 | FBSFWRHWHYMIOG-UHFFFAOYSA-N | C8H8O5 | M183.030T184.303 |
| D-tartaric acid | 0.912909846 | FEWJPZIEWOKRBE-LWMBPPNESA-N | C4H6O6 | M149.009T45.234 |
| Chlorogenic Acid | 0.910981077 | CWVRJTMFETXNAD-JUHZACGLSA-N | C16H18O9 | M353.087T174.723 |
| Phellodendrine chloride | 0.910452385 | RBBVPNQTBKHOEQ-BJQOMGFOSA-O | C20H24NO4+.Cl- | M340.154T268.173 |
| Shanzhiside methyl ester | 0.909214308 | KKSYAZCUYVRKML-IRDZEPHTSA-N | C17H26O11 | M405.139T154.109 |
| Gentisic acid | 0.900860385 | WXTMDXOMEHJXQO-UHFFFAOYSA-N | C7H6O4 | M153.019T167.942 |
| Soyasapogenol B base + O-HexA+HexA+dHex | 0.898255308 | NWEOPKVXZATAQT-UHFFFAOYSA-N | C48H74O19 | M953.475T730.300 |
| (2S,3S,4S,5R,6R)-6-[[(3S,4R,6aR,6bS,8aS,14bR)-4-(hydroxymethyl)-4,6a,6b,11,11,14b-hexamethyl-8a-[(2S,3R,4S,5S,6R)-3,4,5-trihydroxy-6-(hydroxymethyl)oxan-2-yl]oxycarbonyl-1,2,3,4a,5,6,7,8,9,10,12,12a,14,14a-tetradecahydropicen-3-yl]oxy]-3,4,5-trihydroxyoxane-2-carboxylic acid | 0.898161308 | RZQHWSDMLZHIRN-MCTPBCADSA-N | C42H66O15 | M809.430T738.116 |
| cirsimaritin | 0.896882385 | ZIIAJIWLQUVGHB-UHFFFAOYSA-N | C17H14O6 | M313.071T630.512 |
| Sinapic acid | 0.896501538 | PCMORTLOPMLEFB-ONEGZZNKSA-N | C11H12O5 | M223.061T319.719 |
| Loganic acid | 0.890912077 | JNNGEAWILNVFFD-CDJYTOATSA-N | C16H24O10 | M375.129T169.803 |
| LPC 16:0 | 0.889609769 | ASWBNKHCZGQVJV-UHFFFAOYSA-N | C24H50NO7P | M540.330T794.054 |
| Caffeic Acid | 0.888070769 | QAIPRVGONGVQAS-DUXPYHPUSA-N | C9H8O4 | M179.035T210.398 |
| Kaempferol-3-O-glucoside-6''-p-coumaroyl | 0.887675154 | DVGGLGXQSFURLP-UHFFFAOYNA-N | C30H26O13 | M593.127T448.167 |
| Mannose | 0.884210077 | WQZGKKKJIJFFOK-QTVWNMPRSA-N | C6H12O6 | M179.056T857.442 |
| Sorbose | 0.884040385 | BJHIKXHVCXFQLS-OTWZMJIISA-N | C6H12O6 | M179.056T411.223 |
| 9-Hydroxy-10,12,15-octadecatrienoic acid | 0.872122 | RIGGEAZDTKMXSI-UHFFFAOYSA-N | C18H30O3 | M293.211T735.241 |
| Galactose | 0.866138923 | WQZGKKKJIJFFOK-FPRJBGLDSA-N | C6H12O6 | M179.056T150.977 |
| (+/-)-Jasmonic acid | 0.864879 | ZNJFBWYDHIGLCU-UHFFFAOYSA-N | C12H18O3 | M209.118T594.774 |
| Arachidonic acid | 0.863410769 | YZXBAPSDXZZRGB-DOFZRALJSA-N | C20H32O2 | M303.136T441.151 |
| methyl (1S,4aR,7aR)-4a-hydroxy-7-(hydroxymethyl)-1-[(2S,3R,4S,5S,6R)-3,4,5-trihydroxy-6-(hydroxymethyl)oxan-2-yl]oxy-5,7a-dihydro-1H-cyclopenta[c]pyran-4-carboxylate | 0.863398 | LDBMLOLBWUOZGG-DOFVRBEMSA-N | C17H24O11 | M403.123T267.691 |
| 2H-Pyran-4-acetic acid, 5-carboxy-3-ethylidene-2-(beta-D-glucopyranosyloxy)-3,4-dihydro-, alpha-[2-(4-hydroxyphenyl)ethyl] ester, (2S,3Z,4S)- | 0.862698769 | BSUNYCAAALCBNU-MONCMAELSA-N | C24H30O12 | M509.165T462.640 |
| Medicagenic acid | 0.856229385 | IDGXIXSKISLYAC-WNTKNEGGSA-N | C30H46O6 | M501.320T603.173 |
| Gossypetin-8-C-glucoside | 0.841594923 | SJRXVLUZMMDCNG-UHFFFAOYSA-N | C21H20O13 | M479.081T340.659 |
| Piperonylic Acid | 0.839799231 | VDVJGIYXDVPQLP-UHFFFAOYSA-N | C8H6O4 | M165.019T155.155 |
| sweroside | 0.837775923 | VSJGJMKGNMDJCI-ZASXJUAOSA-N | C16H22O9 | M357.118T152.596 |
| 13-HOTrE | 0.834294385 | KLLGGGQNRTVBSU-FQSPHKRJSA-N | C18H30O3 | M293.211T665.454 |
| (2S,3S)-2-(3,4-dihydroxyphenyl)-3,7-dihydroxy-2,3-dihydrochromen-4-one | 0.833961769 | FNUPUYFWZXZMIE-CABCVRRESA-N | C15H12O6 | M287.056T428.149 |
| P-Anisic acid | 0.833951385 | ZEYHEAKUIGZSGI-UHFFFAOYSA-N | C8H8O3 | M151.040T205.120 |
| DIGOXIGENIN | 0.832393769 | SHIBSTMRCDJXLN-KCZCNTNESA-N | C23H34O5 | M389.235T903.747 |
| Jasmonic acid | 0.832231769 | ZNJFBWYDHIGLCU-HWKXXFMVSA-N | C12H18O3 | M209.118T559.779 |
| Isofraxidin | 0.832046538 | HOEVRHHMDJKUMZ-UHFFFAOYSA-N | C11H10O5 | M221.045T299.635 |
| 2-O-methyl 4a-O-[(2S,3R,4S,5S,6R)-3,4,5-trihydroxy-6-(hydroxymethyl)oxan-2-yl] (2S,4aR,6aS,6bR,9R,10R,11S,12aR,14bS)-11-hydroxy-9-(hydroxymethyl)-2,6a,6b,9,12a-pentamethyl-10-[(2S,3R,4S,5R)-3,4,5-trihydroxyoxan-2-yl]oxy-1,3,4,5,6,6a,7,8,8a,10,11,12,13,14b-tetradecahydropicene-2,4a-dicarboxylate | 0.827966923 | XPZGXZBKRPZWMJ-NSUQAVABSA-N | C42H66O16 | M825.426T522.273 |
| Dihydroartemisinin | 0.824072846 | BJDCWCLMFKKGEE-KXTPALSWSA-N | C15H24O5 | M283.154T329.724 |
| [(1S,3R,5R,6S,8S)-3-{[(2S,3R,4S,5S,6R)-6-[(benzoyloxy)methyl]-3,4,5-trihydroxyoxan-2-yl]oxy}-6-hydroxy-8-methyl-9,10-dioxatetracyclo[4.3.1.0²,⁵.0³,⁸]decan-2-yl]methyl benzoate | 0.823645538 | LATYEZNGPQKAIK-BRVOSZROSA-N | C30H32O12 | M629.187T507.957 |
| methyl (1S)-7-hydroxy-7-methyl-1-[(2S,3R,4S,5S,6R)-3,4,5-trihydroxy-6-(hydroxymethyl)oxan-2-yl]oxy-4a,5,6,7a-tetrahydro-1H-cyclopenta[c]pyran-4-carboxylate | 0.822442538 | XBGJTRDIWPEIMG-YVAUHRMASA-N | C17H26O10 | M389.143T190.653 |
| Flavanone + 3O, O-Hex | 0.817385231 | DLIKSSGEMUFQOK-UHFFFAOYSA-N | C21H22O10 | M433.112T388.102 |
| Octyl gallate | 0.812523769 | NRPKURNSADTHLJ-UHFFFAOYSA-N | C15H22O5 | M281.139T545.517 |
| Santonin | 0.810510769 | XJHDMGJURBVLLE-BOCCBSBMSA-N | C15H18O3 | M245.118T538.804 |
| Taxifolin | 0.808904 | CXQWRCVTCMQVQX-LSDHHAIUSA-N | C15H12O7 | M303.051T428.690 |
| 3-hydroxybenzoic acid | 0.805409923 | IJFXRHURBJZNAO-UHFFFAOYSA-N | C7H6O3 | M137.024T224.532 |
| Azelaic acid | 0.802398154 | BDJRBEYXGGNYIS-UHFFFAOYSA-N | C9H16O4 | M187.097T397.204 |
| Pectolinarin | 0.799052615 | DUXQKCCELUKXOE-UHFFFAOYSA-N | C29H34O15 | M621.180T408.536 |
| Calcium pantothenate | 0.795941462 | FAPWYRCQGJNNSJ-UBKPKTQASA-L | C9H17NO5.1/2 Ca | M218.103T93.702 |
| Sucrose | 0.795094923 | CZMRCDWAGMRECN-UGDNZRGBSA-N | C12H22O11 | M341.108T1734.190 |
| Shikimic acid | 0.791658231 | JXOHGGNKMLTUBP-HSUXUTPPSA-N | C7H10O5 | M173.045T76.201 |
| D-(+)-Malic acid | 0.791548231 | BJEPYKJPYRNKOW-UWTATZPHSA-N | C4H6O5 | M133.014T193.809 |
| Vanillic acid | 0.790693 | WKOLLVMJNQIZCI-UHFFFAOYSA-N | C8H8O4 | M167.034T261.551 |
| Isomucronulatol 7-O-glucoside | 0.787205462 | SXHOGLPTLQBGDO-ZVSSUSCDSA-N | C23H28O10 | M463.161T465.925 |
| Pennogenin 3-O-beta-chacotrioside | 0.787193308 | NABPSKKFOWENEB-KUYDPMQHSA-N | C45H72O17 | M883.467T684.642 |
| Saikosaponin b2 | 0.783595692 | WRYJYFCCMSVEPQ-UHFFFAOYSA-N | C42H68O13 | M825.461T641.369 |
| Quillaic acid | 0.780265615 | MQUFAARYGOUYEV-UAWZMHPWSA-N | C30H46O5 | M485.327T688.046 |
| (1S,4aR,6aS,6bR,9R,10R,11R,12aR,14bS)-1,10,11-trihydroxy-9-(hydroxymethyl)-2,2,6a,6b,9,12a-hexamethyl-1,3,4,5,6,6a,7,8,8a,10,11,12,13,14b-tetradecahydropicene-4a-carboxylic acid | 0.776926154 | IFIQVSCCFRXSJV-ZIZFEDMCSA-N | C30H48O6 | M503.337T575.011 |
| Phytolaccagenin | 0.773009538 | CYJWWQALTIKOAG-FLORRLIPSA-N | C31H48O7 | M531.331T648.640 |
| 2H-Pyran-4-acetic acid, 2-(beta-D-glucopyranosyloxy)-3,4-dihydro-3-(2-hydroxyethylidene)-5-(methoxycarbonyl)-, 2-(4-hydroxyphenyl)ethyl ester, (2S,3Z,4S)- | 0.762579846 | AHTRGGWSBFOEEG-BXDNVMCDSA-N | C25H32O13 | M539.175T465.925 |
| (R)-Mandelic acid | 0.761597538 | IWYDHOAUDWTVEP-SSDOTTSWSA-N | C8H8O3 | M151.040T266.768 |
| Stachyose | 0.759177385 | UQZIYBXSHAGNOE-XNSRJBNMSA-N | C24H42O21 | M665.212T1659.480 |
| Sebacic acid | 0.758423077 | CXMXRPHRNRROMY-UHFFFAOYSA-N | C10H18O4 | M201.113T456.300 |
| Hydroxyvalerenic Acid | 0.757595154 | XJNQXTISSHEQKD-UNXUOHHUSA-N | C15H22O3 | M249.149T704.907 |
| (1S,4aR,6aS,6bR,9S,10R,11R,12aR,14bS)-1,10,11-trihydroxy-9-(hydroxymethyl)-2,2,6a,6b,9,12a-hexamethyl-1,3,4,5,6,6a,7,8,8a,10,11,12,13,14b-tetradecahydropicene-4a-carboxylic acid | 0.754415923 | IFIQVSCCFRXSJV-UFTBQYHPSA-N | C30H48O6 | M503.337T589.158 |
| Madecassic acid | 0.753014231 | PRAUVHZJPXOEIF-AOLYGAPISA-N | C30H48O6 | M503.337T613.294 |
| Xylose | 0.750788923 | PYMYPHUHKUWMLA-VPENINKCSA-N | C5H10O5 | M149.045T50.396 |
| Ginsenoside F3 | 0.741604462 | HJRVLGWTJSLQIG-UHFFFAOYNA-N | C41H70O13 | M815.476T580.246 |
| Shanzhiside | 0.737676692 | YSIFYNVXJOGADM-KDYWOABDSA-N | C16H24O11 | M391.123T66.535 |
| isosakuranetin-7-O-rutinoside | 0.735847231 | RMCRQBAILCLJGU-UHFFFAOYNA-N | C28H34O14 | M639.190T369.963 |
| (2S,3S)-2-(3,4-dihydroxyphenyl)-3,5,7-trihydroxy-6-methyl-2,3-dihydrochromen-4-one | 0.735633231 | KPCWWZLBHGSXPW-CVEARBPZSA-N | C16H14O7 | M317.067T132.883 |
| Naringenin | 0.732154 | FTVWIRXFELQLPI-UHFFFAOYNA-N | C15H12O5 | M271.060T494.767 |
| PYROCATECHUIC ACID | 0.732123231 | GLDQAMYCGOIJDV-UHFFFAOYSA-N | C7H6O4 | M153.019T219.603 |
| Methylsuccinic acid | 0.728262923 | WXUAQHNMJWJLTG-UHFFFAOYSA-N | C5H8O4 | M131.036T1577.950 |
| Pentose-Hexose + C10H17 | 0.726246462 | IEGFOTASSBZIBZ-UHFFFAOYSA-N | C21H36O10 | M493.228T176.479 |
| Tectoridin | 0.725959462 | CNOURESJATUGPN-UDEBZQQRSA-N | C22H22O11 | M461.107T285.838 |
| Pinocembrin | 0.722578154 | URFCJEUYXNAHFI-UHFFFAOYSA-N | C15H12O4 | M255.065T546.645 |
| Dihydromyricetin | 0.72143 | KJXSIXMJHKAJOD-LSDHHAIUSA-N | C15H12O8 | M319.045T371.576 |
| Phenylalanine | 0.719555923 | COLNVLDHVKWLRT-QMMMGPOBSA-N | C9H11NO2 | M164.071T81.829 |
| Ginsenoside Rg5 | 0.717455923 | NJUXRKMKOFXMRX-UHFFFAOYSA-N | C42H70O12 | M811.484T772.229 |
| Syringetin-3-O-glucoside | 0.716823923 | JMFWYRWPJVEZPV-UHFFFAOYNA-N | C23H24O13 | M507.113T390.729 |
| Naringenin chalcone | 0.709152923 | YQHMWTPYORBCMF-ZZXKWVIFSA-N | C15H12O5 | M271.060T576.636 |
| Oleic acid | 0.707996538 | ZQPPMHVWECSIRJ-KTKRTIGZSA-N | C18H34O2 | M281.248T965.349 |
| Bruceine A | 0.707301615 | LPZSTPCYWWRQFU-VILODJCFSA-N | C26H34O11 | M521.203T388.366 |
| phellopterin | 0.707012385 | BMLZFLQMBMYVHG-UHFFFAOYSA-N | C17H16O5 | M299.092T501.238 |
| Aconitic Acid | 0.704779615 | GTZCVFVGUGFEME-IWQZZHSRSA-N | C6H6O6 | M173.009T50.396 |
| Aurantio-obtusin beta-D-glucoside | 0.698654308 | LQYQYAJWKXDTHR-PHVGODQESA-N | C23H24O12 | M491.118T341.555 |
| Ginsenoside Rg3(S-FORM) | 0.697899 | RWXIFXNRCLMQCD-UHFFFAOYSA-N | C42H72O13 | M829.495T690.016 |
| DIMETHYLCAFFEIC ACID | 0.693637846 | HJBWJAPEBGSQPR-GQCTYLIASA-N | C11H12O4 | M207.066T225.582 |
| 5,9-dihydroxy-5,7,7-trimethyl-4,5a,6,8,8a,9-hexahydro-1H-azuleno[5,6-c]furan-3-one | 0.690463846 | UUZWMJQAEYBHAO-UHFFFAOYSA-N | C15H22O4 | M265.143T581.616 |
| 10-Deacetylbaccatin III | 0.686895538 | YWLXLRUDGLRYDR-ZHPRIASZSA-N | C29H36O10 | M543.225T548.338 |
| Isochlorogenic acid B | 0.679497 | UFCLZKMFXSILNL-BBLPPJRLSA-N | C25H24O12 | M515.124T49.895 |
| 3-phenyllactic acid | 0.676737538 | VOXXWSYKYCBWHO-UHFFFAOYSA-N | C9H10O3 | M165.055T309.189 |
| Tetrasaccharides (Hex-Hex-Hex-Hex) | 0.676205692 | UQZIYBXSHAGNOE-UHFFFAOYSA-N | C24H42O21 | M711.219T1623.170 |
| Phenyl-beta-glucopyranoside | 0.675633231 | NEZJDVYDSZTRFS-RMPHRYRLSA-N | C12H16O6 | M255.087T115.445 |
| Kaempferide | 0.673787231 | SQFSKOYWJBQGKQ-UHFFFAOYSA-N | C16H12O6 | M299.055T513.044 |
| Asperulosidic acid | 0.673319 | DGDWCRWJRNMRKX-DILZHRMZSA-N | C18H24O12 | M431.118T88.069 |
| Astragaloside IV | 0.670545923 | QMNWISYXSJWHRY-CSXKERSZSA-N | C41H68O14 | M829.457T536.692 |
| [(2R,3R,4S,5R,6R)-6-[2-(3,4-dihydroxyphenyl)ethoxy]-3,5-dihydroxy-4-[(2R,3R,4R,5R,6S)-3,4,5-trihydroxy-6-methyloxan-2-yl]oxyoxan-2-yl]methyl (E)-3-(3,4-dihydroxyphenyl)prop-2-enoate | 0.669596692 | FNMHEHXNBNCPCI-GCELSKRESA-N | C29H36O15 | M623.197T376.850 |
| Austricine | 0.668908308 | YMUOZXZDDBRJEP-UHFFFAOYSA-N | C15H18O4 | M261.113T614.982 |
| Orcinol | 0.668313538 | OIPPWFOQEKKFEE-UHFFFAOYSA-N | C7H8O2 | M123.045T88.522 |
| (R,R)-TARTARIC ACID | 0.667812077 | FEWJPZIEWOKRBE-UHFFFAOYSA-N | C4H6O6 | M149.009T183.201 |
| 4-Methoxysalicylic acid | 0.665709692 | MRIXVKKOHPQOFK-UHFFFAOYSA-N | C8H8O4 | M167.035T88.069 |
| Dehydroandrographolidesuccinate | 0.663003538 | YTHKMAIVPFVDNU-GPTWTFMPSA-N | C28H36O10 | M531.221T451.184 |
| isosakuranetin-7-O-neohesperidoside | 0.659008615 | NLAWPKPYBMEWIR-UHFFFAOYNA-N | C28H34O14 | M639.193T328.291 |
| Eriodictyol | 0.655342538 | SBHXYTNGIZCORC-ZDUSSCGKSA-N | C15H12O6 | M287.056T470.964 |
| Rehmannioside A | 0.654641615 | DTNSOISBYQKHCS-XTOBSYSVSA-N | C21H32O15 | M569.171T64.463 |
| (-)-12-hydroxyjasmonic acid | 0.653100385 | RZGFUGXQKMEMOO-BSANDHCLSA-N | C12H18O4 | M225.113T478.730 |
| Calceolarioside B | 0.652296923 | LFKQVVDFNHDYNK-FOXCETOMSA-N | C23H26O11 | M477.145T39.380 |
| Isocitrate | 0.647802385 | ODBLHEXUDAPZAU-UHFFFAOYSA-N | C6H8O7 | M173.009T39.348 |
| (1S,3R,4S,5R)-4-{[(2E)-3-(3,4-dihydroxyphenyl)prop-2-enoyl]oxy}-1,3,5-trihydroxycyclohexane-1-carboxylic acid | 0.644286154 | GYFFKZTYYAFCTR-JUHZACGLSA-N | C16H18O9 | M353.086T206.796 |
| Coumaroyl quinic acid | 0.640392538 | BMRSEYFENKXDIS-UHFFFAOYSA-N | C16H18O8 | M337.092T253.077 |
| 18b-glycyrrhetinic acid | 0.640288769 | MPDGHEJMBKOTSU-AWOGJAJBSA-N | C30H46O4 | M469.332T770.531 |
| 7,4'-Dimethoxy-5-hydroxyflavanone | 0.638744769 | CKEXCBVNKRHAMX-UHFFFAOYSA-N | C17H16O5 | M299.092T487.800 |
| Loganin | 0.637273308 | AMBQHHVBBHTQBF-UHFFFAOYNA-N | C17H26O10 | M435.150T214.723 |
| Coumaroyl Hexoside | 0.636245615 | DSNCQKUYZOSARM-UHFFFAOYSA-N | C15H18O8 | M325.092T136.687 |
| Ajugol | 0.636008077 | VELYAQRXBJLJAK-XKKWFBPMSA-N | C15H24O9 | M393.139T112.649 |
| (2R,3S,4S,5R,6R)-5-[(2S,3R,4R)-3,4-dihydroxy-4-(hydroxymethyl)oxolan-2-yl]oxy-2-(hydroxymethyl)-6-(2-phenylethoxy)oxane-3,4-diol | 0.635630231 | UWKRNCNWJVCHGZ-DERWZFJFSA-N | C19H28O10 | M415.160T323.953 |
| Isoginkgetin | 0.632009692 | HUOOMAOYXQFIDQ-UHFFFAOYSA-N | C32H22O10 | M565.113T669.048 |
| Taxifolin 7-rhamnoside | 0.631191615 | HNGAZJABJOAMSW-HVKWJXAYSA-N | C21H22O11 | M449.108T257.357 |
| Petunidin-3-O-beta-glucopyranoside | 0.629232077 | CCQDWIRWKWIUKK-QKYBYQKWSA-O | C22H23O12 | M477.103T381.589 |
| 17-Hydroxyisolathyrol | 0.627930077 | XKXYJTIBKZGIPR-YDYNUCMUSA-N | C20H30O5 | M349.205T879.970 |
| MGMG 18:2 | 0.626539308 | LBHUIJRTHBBWHP-UHFFFAOYSA-N | C27H48O9 | M561.328T810.301 |
| Gallic acid hexoside | 0.624379077 | GDVRUDXLQBVIKP-UHFFFAOYSA-N | C13H16O10 | M331.067T77.891 |
| Morroniside | 0.621464769 | YTZSBJLNMIQROD-SFBCHFHNSA-N | C17H26O11 | M451.145T191.800 |
| (2S,3S)-3,5,7-trihydroxy-6-methyl-2-(3,4,5-trihydroxyphenyl)-2,3-dihydrochromen-4-one | 0.617331692 | NZRLODDQFPZTPM-CVEARBPZSA-N | C16H14O8 | M333.061T288.986 |
| Hypocrellin B | 0.617295231 | SBMXTMAIKRQSQE-UHFFFAOYSA-N | C30H26O10 | M527.141T257.357 |
| Ecliptasaponin A | 0.611162308 | WYDPEADEZMZKNM-ZBKPBKBGSA-N | C36H58O9 | M633.399T667.631 |
| (2R,3R,4R,5R,6S)-2-[[(2R,3S,4S,5R,6S)-6-(4-ethenylphenoxy)-3,4,5-trihydroxyoxan-2-yl]methoxy]-6-methyloxane-3,4,5-triol | 0.609327615 | KVPBAPOAIIQDGQ-XXGVTNRTSA-N | C20H28O10 | M427.159T404.880 |
| D-Gluconic acid | 0.604149846 | RGHNJXZEOKUKBD-UHFFFAOYSA-N | C6H12O7 | M195.050T39.449 |
| 3,5,5-Trimethyl-4-[3-[3,4,5-trihydroxy-6-(hydroxymethyl)oxan-2-yl]oxybut-1-enyl]cyclohex-2-en-1-one | 0.603281308 | SZOPSAFLRCYJCX-UHFFFAOYSA-N | C19H30O7 | M415.196T401.353 |
| Azuleno(5,6-c)furan-1(3H)-one, 4,4a,5,6,7,7a,8,9-octahydro-3,4,8-trihydroxy-6,6,8-trimethyl- | 0.602784769 | MWDNWQAVYQDZQI-UHFFFAOYSA-N | C15H22O5 | M281.139T497.284 |


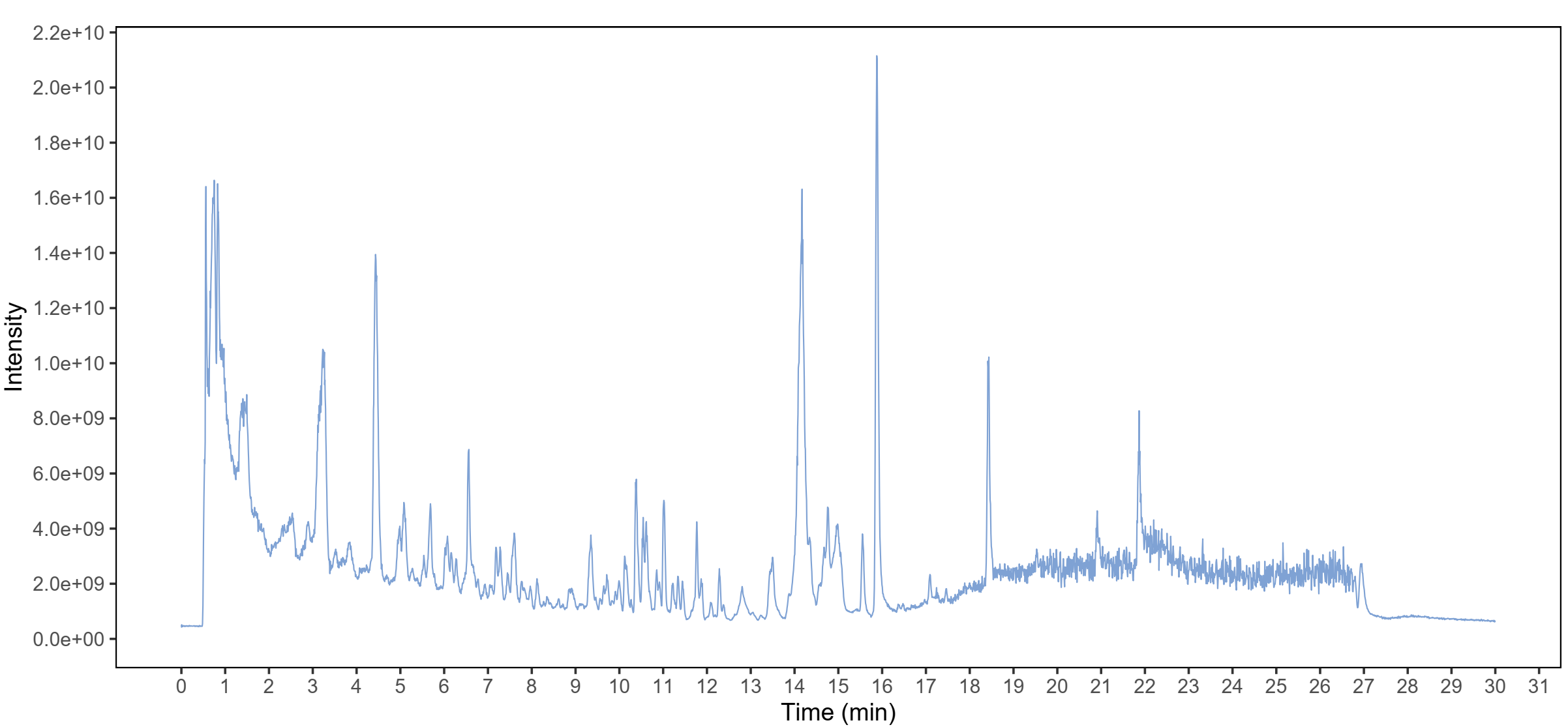


Fig1-2 POS-T

Table 1-2 POS-final

| **NameEN** | **CompositeScore** | **InChIKey** | **Formula** | **name** |  |  |  |
| --- | --- | --- | --- | --- | --- | --- | --- |
| (-)-Epicatechin | 1 | PFTAWBLQPZVEMU-UKRRQHHQSA-N | C15H14O6 | M291.086T423.333 |  |  |  |
| (1R,2E,7R,10E,12S,13S,15R)-12,15-dihydroxy-7-methyl-8-oxabicyclo[11.3.0]hexadeca-2,10-dien-9-one | 1 | KQNZDYYTLMIZCT-SWGFJCPHSA-N | C16H24O4 | M319.130T756.713 |  |  |  |
| (1R,7R)-7-ethenyl-1,4a,7-trimethyl-3,4,4b,5,6,9,10,10a-octahydro-2H-phenanthrene-1-carboxylic acid | 1 | MHVJRKBZMUDEEV-WANGQUDJSA-N | C20H30O2 | M320.256T953.648 |  |  |  |
| (2R,3R,4S,5S,6R)-2-[(2E)-4-hydroxy-3,7-dimethylocta-2,6-dienoxy]-6-(hydroxymethyl)oxane-3,4,5-triol | 1 | PBPYEEMQIFDGSQ-MIYBPCGNSA-N | C16H28O7 | M371.147T391.373 |  |  |  |
| [2,3-dihydroxy-1-(7-methoxy-2-oxochromen-6-yl)-3-methylbutyl] (Z)-2-methylbut-2-enoate | 1 | BAHUBXAYVOCLNA-WDZFZDKYSA-N | C20H24O7 | M377.157T460.243 |  |  |  |
| 2-AMINOPHENOL | 1 | PEXGTUZWTLMFID-UHFFFAOYSA-N | C6H7NO | M110.060T49.667 |  |  |  |
| 4-Hydroxybenzaldehyde | 1 | RGHHSNMVTDWUBI-UHFFFAOYSA-N | C7H6O2 | M123.044T466.405 |  |  |  |
| 5,6,7,8-tetrahydroxy-2-(4-hydroxyphenyl)chromen-4-one | 1 | SPZXXUUDYMHBSG-UHFFFAOYSA-N | C15H10O7 | M303.051T290.422 |  |  |  |
| 6-(1,1-DIMETHYLALLYL)-2-(1-HYDROXY-1-METHYLETHYL)-2,3-DIHYDRO-7H-FURO[3,2-G]CHROMEN-7-ONE | 1 | JCDLLLXYAICSQV-INIZCTEOSA-N | C19H22O4 | M337.143T737.032 |  |  |  |
| Acetophenone | 1 | KWOLFJPFCHCOCG-UHFFFAOYSA-N | C8H8O | M121.065T881.552 |  |  |  |
| Adenine | 1 | GFFGJBXGBJISGV-UHFFFAOYSA-N | C5H5N5 | M136.062T49.667 |  |  |  |
| Anisic aldehyde | 1 | ZRSNZINYAWTAHE-UHFFFAOYSA-N | C8H8O2 | M137.060T82.674 |  |  |  |
| Apigenin-7-O-glucoside | 1 | KMOUJOKENFFTPU-QNDFHXLGSA-N | C21H20O10 | M433.114T356.059 |  |  |  |
| Berberine | 1 | YBHILYKTIRIUTE-UHFFFAOYSA-N | C20H18NO4+ | M336.121T435.520 |  |  |  |
| Bergenin | 1 | YWJXCIXBAKGUKZ-HJJNZUOJSA-N | C14H16O9 | M351.068T43.194 |  |  |  |
| beta-D-Glucopyranoside, phenylmethyl 6-O-[(2R,3R,4R)-tetrahydro-3,4-dihydroxy-4-(hydroxymethyl)-2-furanyl]- | 1 | NJMQSVWMCODQIP-FQXXIRCGSA-N | C18H26O10 | M425.142T253.560 |  |  |  |
| Calceolarioside B | 1 | LFKQVVDFNHDYNK-FOXCETOMSA-N | C23H26O11 | M479.156T297.361 |  |  |  |
| Carpachromene | 1 | YXOATFKTEDZPFL-UHFFFAOYSA-N | C20H16O5 | M337.107T680.067 |  |  |  |
| Chaulmoogric Acid | 1 | XMVQWNRDPAAMJB-UHFFFAOYSA-N | C18H32O2 | M298.275T798.132 |  |  |  |
| Chrysophanol 8-O-beta-D-glucoside | 1 | WMMOMSNMMDMSRB-JNHRPPPUSA-N | C21H20O9 | M439.100T398.749 |  |  |  |
| Cinnamamide | 1 | APEJMQOBVMLION-VOTSOKGWSA-N | C9H9NO | M148.076T322.885 |  |  |  |
| Cinnamic acid | 1 | WBYWAXJHAXSJNI-VOTSOKGWSA-N | C9H8O2 | M149.060T81.703 |  |  |  |
| Corosolic acid | 1 | HFGSQOYIOKBQOW-ZSDYHTTISA-N | C30H48O4 | M473.362T782.572 |  |  |  |
| Dibutylphthalate | 1 | DOIRQSBPFJWKBE-UHFFFAOYSA-N | C16H22O4 | M279.157T442.349 |  |  |  |
| eudesmin | 1 | PEUUVVGQIVMSAW-RZTYQLBFSA-N | C22H26O6 | M425.136T636.545 |  |  |  |
| geniposide | 1 | IBFYXTRXDNAPMM-BVTMAQQCSA-N | C17H24O10 | M411.127T258.372 |  |  |  |
| Glyceryl linolenate | 1 | GGJRAQULURVTAJ-PDBXOOCHSA-N | C21H36O4 | M375.250T844.285 |  |  |  |
| Hypocrellin B | 1 | SBMXTMAIKRQSQE-UHFFFAOYSA-N | C30H26O10 | M529.153T316.066 |  |  |  |
| isoimperatorin | 1 | IGWDEVSBEKYORK-UHFFFAOYSA-N | C16H14O4 | M293.079T676.754 |  |  |  |
| Karakoline | 1 | HKQZUYOVMYOFIT-UHFFFAOYSA-N | C22H35NO4 | M378.262T577.861 |  |  |  |
| N-methylnicotinate | 1 | WWNNZCOKKKDOPX-UHFFFAOYSA-N | C7H7NO2 | M138.055T53.760 |  |  |  |
| Norharman | 1 | AIFRHYZBTHREPW-UHFFFAOYSA-N | C11H8N2 | M169.076T259.985 |  |  |  |
| Periplogenin | 1 | QJPCKAJTLHDNCS-FBAXFMHRSA-N | C23H34O5 | M391.246T768.161 |  |  |  |
| Phlorizin | 1 | IOUVKUPGCMBWBT-QNDFHXLGSA-N | C21H24O10 | M437.143T365.844 |  |  |  |
| rutamarin | 1 | AWMHMGFGCLBSAY-UHFFFAOYSA-N | C21H24O5 | M379.152T599.479 |  |  |  |
| Stepharine | 1 | OGJKMZVUJJYWKO-UHFFFAOYSA-N | C18H19NO3 | M298.142T622.721 |  |  |  |
| LOVASTATIN | 0.999996 | PCZOHLXUXFIOCF-BXMDZJJMSA-N | C24H36O5 | M405.262T793.486 |  |  |  |
| Luteolin | 0.999952538 | IQPNAANSBPBGFQ-UHFFFAOYSA-N | C15H10O6 | M287.056T508.995 |  |  |  |
| Isopropyl 4-Hydroxybenzoate | 0.99994 | CMHMMKSPYOOVGI-UHFFFAOYSA-N | C10H12O3 | M181.086T709.575 |  |  |  |
| [(2R)-2-[(E,2S,4R)-4,6-dimethyloct-6-en-2-yl]-6-oxo-2,3-dihydropyran-3-yl] (2E,4E,6S)-8-hydroxy-6-(hydroxymethyl)-4-methylocta-2,4-dienoate | 0.999926 | OHRGHFXATDKGOV-MDKHPKFKSA-N | C25H38O6 | M435.272T769.846 |  |  |  |
| 7-Methoxy-4-methylcoumarin | 0.999883 | UDFPKNSWSYBIHO-UHFFFAOYSA-N | C11H10O3 | M191.070T531.935 |  |  |  |
| Deoxyandrographolide | 0.999809231 | DAXSYTVXDSOSIE-HNJRGHQBSA-N | C20H30O4 | M335.219T655.374 |  |  |  |
| Caudatin | 0.999778615 | VWLXIXALPNYWFH-UBHIOMQOSA-N | C28H42O7 | M491.298T860.060 |  |  |  |
| Withanone | 0.999524769 | FAZIYUIDUNHZRG-UHFFFAOYSA-N | C28H38O6 | M471.276T984.638 |  |  |  |
| Alisol B 23-acetate | 0.999521769 | NLOAQXKIIGTTRE-CXWFPJGHSA-N | C32H50O5 | M515.372T1141.160 |  |  |  |
| monolinolein | 0.999473 | WECGLUPZRHILCT-HZJYTTRNSA-N | C21H38O4 | M355.282T937.898 |  |  |  |
| Genistein | 0.999301923 | TZBJGXHYKVUXJN-UHFFFAOYSA-N | C15H10O5 | M271.061T542.220 |  |  |  |
| Atractylenolide III | 0.998163615 | FBMORZZOJSDNRQ-GLQYFDAESA-N | C15H20O3 | M249.148T651.229 |  |  |  |
| Glabrolide | 0.998155615 | SSHDNSCEQSPWIM-FVTWEACWSA-N | C30H44O4 | M469.333T748.544 |  |  |  |
| Proline | 0.997824308 | ONIBWKKTOPOVIA-UHFFFAOYSA-N | C5H9NO2 | M116.071T45.136 |  |  |  |
| Piperine | 0.997654923 | MXXWOMGUGJBKIW-YPCIICBESA-N | C17H19NO3 | M286.144T646.493 |  |  |  |
| syringin | 0.997569308 | QJVXKWHHAMZTBY-GCPOEHJPSA-N | C17H24O9 | M373.149T265.634 |  |  |  |
| 8-hydroxy-5,7-dimethoxy-2-phenyl-2,3-dihydrochromen-4-one | 0.997154462 | TVWVHFSJQNXIRZ-UHFFFAOYSA-N | C17H16O5 | M301.107T574.075 |  |  |  |
| methyl (1S)-7-hydroxy-7-methyl-1-[(2S,3R,4S,5S,6R)-3,4,5-trihydroxy-6-(hydroxymethyl)oxan-2-yl]oxy-4a,5,6,7a-tetrahydro-1H-cyclopenta[c]pyran-4-carboxylate | 0.996512769 | XBGJTRDIWPEIMG-YVAUHRMASA-N | C17H26O10 | M413.141T265.634 |  |  |  |
| Nicotinic acid | 0.996483462 | PVNIIMVLHYAWGP-UHFFFAOYSA-N | C6H5NO2 | M124.039T49.667 |  |  |  |
| epsilon-Viniferin | 0.996475615 | FQWLMRXWKZGLFI-YVYUXZJTSA-N | C28H22O6 | M455.154T323.395 |  |  |  |
| Spiraeoside | 0.995507846 | OIUBYZLTFSLSBY-HMGRVEAOSA-N | C21H20O12 | M465.102T290.422 |  |  |  |
| Chelidonine | 0.994600846 | GHKISGDRQRSCII-ZOCIIQOWSA-N | C20H19NO5 | M354.134T383.620 |  |  |  |
| 5-Hydroxy-1-tetralone | 0.993837692 | YPPZCRZRQHFRBH-UHFFFAOYSA-N | C10H10O2 | M163.076T50.272 |  |  |  |
| Apigenin | 0.993010615 | KZNIFHPLKGYRTM-UHFFFAOYSA-N | C15H10O5 | M271.061T498.904 |  |  |  |
| Rubiadin | 0.992899846 | IRZTUXPRIUZXMP-UHFFFAOYSA-N | C15H10O4 | M255.065T611.115 |  |  |  |
| Wogonin | 0.992814308 | XLTFNNCXVBYBSX-UHFFFAOYSA-N | C16H12O5 | M285.075T8.832 |  |  |  |
| 1,5,9-trihydroxy-5,7,7-trimethyl-4,5a,6,8,8a,9-hexahydro-1H-azuleno[5,6-c]furan-3-one | 0.992567846 | MWDNWQAVYQDZQI-UHFFFAOYSA-N | C15H22O5 | M305.136T466.405 |  |  |  |
| Alisol C 23-acetate | 0.991712769 | KOOCQNIPRJEMDH-QSKXMHMESA-N | C32H48O6 | M529.350T1013.160 |  |  |  |
| 1-methoxyindole-3-carbaldehyde | 0.991593692 | NFGIENSPALNOON-UHFFFAOYSA-N | C10H9NO2 | M176.070T87.902 |  |  |  |
| Cianidanol | 0.991433 | PFTAWBLQPZVEMU-DZGCQCFKSA-N | C15H14O6 | M291.086T412.879 |  |  |  |
| Betaine | 0.990097692 | KWIUHFFTVRNATP-UHFFFAOYSA-N | C5H11NO2 | M118.086T1610.080 |  |  |  |
| Ajugol | 0.989495077 | VELYAQRXBJLJAK-XKKWFBPMSA-N | C15H24O9 | M371.131T112.111 |  |  |  |
| Herbacetin | 0.987700385 | ZDOTZEDNGNPOEW-UHFFFAOYSA-N | C15H10O7 | M303.050T340.010 |  |  |  |
| Dioscin | 0.987507385 | VNONINPVFQTJOC-ZGXDEBHDSA-N | C45H72O16 | M869.480T894.079 |  |  |  |
| (+)-beta-Hydrastine | 0.986632077 | JZUTXVTYJDCMDU-RBUKOAKNSA-N | C21H21NO6 | M384.143T523.061 |  |  |  |
| 3-[(3S,5R,10S,13R,14S,17S)-3-[(2R,3S,4R,5S,6S)-3,5-dihydroxy-4-methoxy-6-methyloxan-2-yl]oxy-14-hydroxy-10,13-dimethyl-1,2,3,4,5,6,7,8,9,11,12,15,16,17-tetradecahydrocyclopenta[a]phenanthren-17-yl]-2H-furan-5-one | 0.986409769 | VPUNMTHWNSJUOG-JGFVFIPGSA-N | C30H46O8 | M535.325T861.627 |  |  |  |
| (1S,2R,5R,6R,10R,13S,15S)-5-[(2R,3E,5R)-5,6-dimethylhept-3-en-2-yl]-6,10-dimethyl-16,17-dioxapentacyclo[13.2.2.0¹,⁹.0²,⁶.0¹⁰,¹⁵]nonadec-18-en-13-ol | 0.985830231 | VXOZCESVZIRHCJ-LNSUSAKYSA-N | C28H44O3 | M467.295T699.204 |  |  |  |
| Formononetin | 0.985544538 | HKQYGTCOTHHOMP-UHFFFAOYSA-N | C16H12O4 | M291.063T562.245 |  |  |  |
| Curcumenol | 0.982865154 | ISFMXVMWEWLJGJ-UHFFFAOYSA-N | C15H22O2 | M235.169T761.361 |  |  |  |
| (4aS,6aS,6bR,9R,10R,11R,12aR)-10,11-dihydroxy-9-(hydroxymethyl)-2,2,6a,6b,9,12a-hexamethyl-1,3,4,5,6,6a,7,8,8a,10,11,12,13,14b-tetradecahydropicene-4a-carboxylic acid | 0.982811846 | RWNHLTKFBKYDOJ-IJDLJTCJSA-N | C30H48O5 | M489.356T1041.300 |  |  |  |
| 3,5-Dimethoxy-4-hydroxybenzaldehyde | 0.982525231 | KCDXJAYRVLXPFO-UHFFFAOYSA-N | C9H10O4 | M183.065T194.361 |  |  |  |
| Salicylic acid | 0.981189385 | YGSDEFSMJLZEOE-UHFFFAOYSA-N | C7H6O3 | M139.039T191.652 |  |  |  |
| Coumaric acid | 0.981085308 | KKSDGJDHHZEWEP-UHFFFAOYSA-N | C9H8O3 | M147.044T374.765 |  |  |  |
| Purpurin | 0.978596846 | BBNQQADTFFCFGB-UHFFFAOYSA-N | C14H8O5 | M257.043T377.578 |  |  |  |
| Atractylenolide II | 0.978237308 | OQYBLUDOOFOBPO-KCQAQPDRSA-N | C15H20O2 | M233.153T722.269 |  |  |  |
| (1S,2R,4aS,6aR,6bR,10S,12aR)-10-[(2R,3R,4S,5R,6S)-3,4-dihydroxy-6-methyl-5-[(2S,3R,4S,5S,6R)-3,4,5-trihydroxy-6-(hydroxymethyl)oxan-2-yl]oxyoxan-2-yl]oxy-1,2,6b,9,9,12a-hexamethyl-2,3,4,5,6,6a,7,8,8a,10,11,12,13,14b-tetradecahydro-1H-picene-4a,6a-dicarboxylic acid | 0.977246923 | IIIOQVDDEWZCEQ-YKQGQIHISA-N | C42H66O14 | M833.412T647.549 |  |  |  |
| Sarracenin | 0.974018385 | QGBCGMGBGAHJIT-UHFFFAOYSA-N | C11H14O5 | M227.092T393.574 |  |  |  |
| Biochanin A | 0.973344692 | WUADCCWRTIWANL-UHFFFAOYSA-N | C16H12O5 | M285.075T629.237 |  |  |  |
| Gal(alpha1-6)Glc(alpha1-2beta)Fruf | 0.972171308 | MUPFEKGTMRGPLJ-OARNWLFOSA-N | C18H32O16 | M527.153T443.031 |  |  |  |
| Valine | 0.969791769 | KZSNJWFQEVHDMF-BYPYZUCNSA-N | C5H11NO2 | M118.086T1622.290 |  |  |  |
| (2S,3S,4S,5R,6R)-6-[[(3S,4S,6aR,6bS,8aR,9R,12aS,14bR)-9-hydroxy-4-(hydroxymethyl)-4,6a,6b,8a,11,11,14b-heptamethyl-1,2,3,4a,5,6,7,8,9,10,12,12a,14,14a-tetradecahydropicen-3-yl]oxy]-5-[(2S,3R,4S,5R,6R)-4,5-dihydroxy-6-(hydroxymethyl)-3-[(2S,3R,4R,5R,6S)-3,4,5-trihydroxy-6-methyloxan-2-yl]oxyoxan-2-yl]oxy-3,4-dihydroxyoxane-2-carboxylic acid | 0.969076385 | PTDAHAWQAGSZDD-VJQGOKKYSA-N | C48H78O18 | M943.519T609.582 |  |  |  |
| Arginine | 0.968978692 | ODKSFYDXXFIFQN-UHFFFAOYSA-N | C6H14N4O2 | M175.119T54.423 |  |  |  |
| Vindoline | 0.968801308 | CXBGOBGJHGGWIE-KKFURDAFSA-N | C25H32N2O6 | M457.236T810.399 |  |  |  |
| (+/-)-Jasmonic acid | 0.968195538 | ZNJFBWYDHIGLCU-UHFFFAOYSA-N | C12H18O3 | M211.133T502.849 |  |  |  |
| Tectochrysin | 0.967571769 | IRZVHDLBAYNPCT-UHFFFAOYSA-N | C16H12O4 | M269.081T470.195 |  |  |  |
| (2R,3R,4R,5R,6S)-2-[[(2R,3S,4S,5R,6S)-6-(4-ethenylphenoxy)-3,4,5-trihydroxyoxan-2-yl]methoxy]-6-methyloxane-3,4,5-triol | 0.967357615 | KVPBAPOAIIQDGQ-XXGVTNRTSA-N | C20H28O10 | M429.173T374.882 |  |  |  |
| Palmatine | 0.965653 | QUCQEUCGKKTEBI-UHFFFAOYSA-N | C21H22NO4 | M352.154T445.218 |  |  |  |
| 4-[(E)-2-(3,5-dimethoxyphenyl)ethenyl]phenol | 0.964897462 | VLEUZFDZJKSGMX-ONEGZZNKSA-N | C16H16O3 | M257.114T476.851 |  |  |  |
| Cryptotanshinone | 0.963922692 | GVKKJJOMQCNPGB-JTQLQIEISA-N | C19H20O3 | M297.149T756.713 |  |  |  |
| Asparagine | 0.962515154 | DCXYFEDJOCDNAF-UHFFFAOYSA-N | C4H8N2O3 | M133.061T46.395 |  |  |  |
| BIOTIN | 0.961517462 | YBJHBAHKTGYVGT-UHFFFAOYSA-N | C10H16N2O3S | M245.099T138.496 |  |  |  |
| methyl (2S,3R,4S)-3-ethenyl-4-[2-(3,4,5-trihydroxybenzoyl)oxyethyl]-2-[3,4,5-trihydroxy-6-(hydroxymethyl)oxan-2-yl]oxy-3,4-dihydro-2H-pyran-5-carboxylate | 0.961384 | SMTKSCGLXONVGL-FMDXMRPMSA-N | C24H30O14 | M543.170T393.896 |  |  |  |
| (1S,2R,4aS,6aS,6bR,10S,12aR)-10-hydroxy-1,2,6a,6b,9,9,12a-heptamethyl-2,3,4,5,6,6a,7,8,8a,10,11,12,13,14b-tetradecahydro-1H-picene-4a-carboxylic acid | 0.961367615 | WCGUUGGRBIKTOS-POPPTPIPSA-N | C30H48O3 | M457.368T544.477 |  |  |  |
| Choline | 0.960865923 | OEYIOHPDSNJKLS-UHFFFAOYSA-N | C5H14NO | M104.107T41.741 |  |  |  |
| Kojic Acid | 0.959734 | BEJNERDRQOWKJM-UHFFFAOYSA-N | C6H6O4 | M143.034T131.657 |  |  |  |
| Harmane | 0.959359154 | PSFDQSOCUJVVGF-UHFFFAOYSA-N | C12H10N2 | M183.091T300.684 |  |  |  |
| p-Coumaraldehyde | 0.958724615 | CJXMVKYNVIGQBS-OWOJBTEDSA-N | C9H8O2 | M149.060T194.361 |  |  |  |
| Pyocyanin | 0.958682308 | YNCMLFHHXWETLD-UHFFFAOYSA-N | C13H10N2O | M211.087T569.433 |  |  |  |
| Benzoic acid | 0.957115308 | WPYMKLBDIGXBTP-UHFFFAOYSA-N | C7H6O2 | M123.044T194.048 |  |  |  |
| Leucodin | 0.957093846 | BJPSSVHNEGMBDQ-NUZBWSBOSA-N | C15H18O3 | M247.133T531.935 |  |  |  |
| Dehydrocorydaline | 0.956710615 | RFKQJTRWODZPHF-UHFFFAOYSA-N | C22H24NO4+ | M366.170T468.527 |  |  |  |
| Sciadopitysin | 0.956054077 | YCXRBCHEOFVYEN-UHFFFAOYSA-N | C33H24O10 | M581.144T752.061 |  |  |  |
| Kaempferol-3-O-glucoside | 0.955937231 | JPUKWEQWGBDDQB-QSOFNFLRSA-N | C21H20O11 | M449.108T375.831 |  |  |  |
| Vanillin | 0.955097615 | MWOOGOJBHIARFG-UHFFFAOYSA-N | C8H8O3 | M153.055T276.296 |  |  |  |
| syringaresinol | 0.954629769 | KOWMJRJXZMEZLD-VGBAVMOLSA-N | C22H26O8 | M441.152T756.145 |  |  |  |
| Isoleucine | 0.953970154 | AGPKZVBTJJNPAG-UHFFFAOYSA-N | C6H13NO2 | M132.102T56.765 |  |  |  |
| alpha-Linolenic acid | 0.952535615 | DTOSIQBPPRVQHS-PDBXOOCHSA-N | C18H30O2 | M279.232T769.846 |  |  |  |
| Dehydrocostus lactone | 0.950662692 | NETSQGRTUNRXEO-XUXIUFHCSA-N | C15H18O2 | M231.138T695.232 |  |  |  |
| Vincamine | 0.950656692 | RXPRRQLKFXBCSJ-UHFFFAOYNA-N | C21H26N2O3 | M355.202T371.629 |  |  |  |
| trans-Cinnamaldehyde | 0.949810462 | KJPRLNWUNMBNBZ-QPJJXVBHSA-N | C9H8O | M133.065T265.996 |  |  |  |
| Paracetamol | 0.949551846 | RZVAJINKPMORJF-UHFFFAOYSA-N | C8H9NO2 | M152.070T289.334 |  |  |  |
| Kaempferide | 0.949490154 | SQFSKOYWJBQGKQ-UHFFFAOYSA-N | C16H12O6 | M301.070T524.191 |  |  |  |
| Pyroglutamic acid (not validated, isomer of 88) | 0.949387077 | ODHCTXKNWHHXJC-UHFFFAOYSA-N | C5H7NO3 | M130.050T50.272 |  |  |  |
| LPC 16:0 | 0.947786385 | ASWBNKHCZGQVJV-UHFFFAOYSA-N | C24H50NO7P | M496.340T794.053 |  |  |  |
| Coumaroyl tyramine | 0.946382308 | RXGUTQNKCXHALN-UHFFFAOYSA-N | C17H17NO3 | M284.128T422.770 |  |  |  |
| sweroside | 0.944520538 | VSJGJMKGNMDJCI-ZASXJUAOSA-N | C16H22O9 | M381.115T268.336 |  |  |  |
| Pechueloic Acid | 0.942423923 | ZFHSKBJBODQVBX-AXTRIDKLSA-N | C15H20O3 | M249.148T561.918 |  |  |  |
| Remerine | 0.940010692 | JCTYWRARKVGOBK-UHFFFAOYNA-N | C18H17NO2 | M280.133T434.999 |  |  |  |
| Loureirin A | 0.937931769 | RSAIVLRELNGZEY-UHFFFAOYSA-N | C17H18O4 | M287.126T449.752 |  |  |  |
| alpha-Cyperone | 0.936359154 | KUFXJZXMWHNCEH-DOMZBBRYSA-N | C15H22O | M219.175T795.855 |  |  |  |
| Tanshinone IIA | 0.936124308 | HYXITZLLTYIPOF-UHFFFAOYSA-N | C19H18O3 | M295.132T821.163 |  |  |  |
| Vicenin 2 | 0.934936 | FIAAVMJLAGNUKW-UHFFFAOYSA-N | C27H30O15 | M595.164T246.990 |  |  |  |
| trans-4-Coumaric acid | 0.934325769 | NGSWKAQJJWESNS-ZZXKWVIFSA-N | C9H8O3 | M165.055T284.545 |  |  |  |
| Coniferyl aldehyde | 0.933134077 | DKZBBWMURDFHNE-UHFFFAOYSA-N | C10H10O3 | M179.070T651.229 |  |  |  |
| Corydine | 0.932811538 | IDQUPXZJURZAGF-ZDUSSCGKSA-N | C20H23NO4 | M342.171T172.751 |  |  |  |
| Guanine | 0.932188385 | UYTPUPDQBNUYGX-UHFFFAOYSA-N | C5H5N5O | M152.057T51.864 |  |  |  |
| Scoparone | 0.930031846 | GUAFOGOEJLSQBT-UHFFFAOYSA-N | C11H10O4 | M207.065T553.356 |  |  |  |
| Ascorbic acid | 0.929890154 | CIWBSHSKHKDKBQ-JLAZNSOCSA-N | C6H8O6 | M177.054T221.612 |  |  |  |
| Azuleno[5,6-c]furan-1(3H)-one, 4,4a,5,6,7,7a,8,9-octahydro-4,8-dihydroxy-6,6,8-trimethyl- | 0.929626462 | UUZWMJQAEYBHAO-UHFFFAOYSA-N | C15H22O4 | M249.149T618.398 |  |  |  |
| Hecogenin | 0.929117538 | QOLRLLFJMZLYQJ-LOBDNJQFSA-N | C27H42O4 | M431.313T984.346 |  |  |  |
| (2E,4E)-N-(2-methylpropyl)deca-2,4-dienamide | 0.928709077 | MAGQQZHFHJDIRE-BNFZFUHLSA-N | C14H25NO | M224.201T734.154 |  |  |  |
| loganic acid | 0.928632538 | JNNGEAWILNVFFD-CDJYTOATSA-N | C16H24O10 | M377.145T173.305 |  |  |  |
| Dehydrotrametenolic acid | 0.927885538 | QFPLAAZRZNKRRY-GIICLEHTSA-N | C30H46O3 | M455.352T660.986 |  |  |  |
| L-Tyrosine | 0.925778308 | OUYCCCASQSFEME-QMMMGPOBSA-N | C9H11NO3 | M182.081T198.674 |  |  |  |
| 4-[2-[(1R,4aS,5R,6R,8aS)-6-hydroxy-5-(hydroxymethyl)-5,8a-dimethyl-2-methylidene-3,4,4a,6,7,8-hexahydro-1H-naphthalen-1-yl]-1-hydroxyethyl]-2H-furan-5-one | 0.924890462 | KHQNSSJNIXVKMK-QSCDNJGLSA-N | C20H30O5 | M351.214T588.035 |  |  |  |
| Strophanthidin |  | ODJLBQGVINUMMR-HZXDTFASSA-N | | | | C23H32O6 | M405.225T525.979 |
| Formononetin-7-O-glucoside | 0.922524077 | MGJLSBDCWOSMHL-UHFFFAOYSA-N | C22H22O9 | M431.133T431.444 |  |  |  |
| Tyramine | 0.920326846 | DZGWFCGJZKJUFP-UHFFFAOYSA-N | C8H11NO | M121.065T52.840 |  |  |  |
| [(1aS,1bS,2S,5aR,6S,6aS)-1a-(hydroxymethyl)-2-[(2S,3R,4S,5S,6R)-3,4,5-trihydroxy-6-(hydroxymethyl)oxan-2-yl]oxy-2,5a,6,6a-tetrahydro-1bH-oxireno[5,6]cyclopenta[1,3-c]pyran-6-yl] benzoate | 0.920312538 | GOHHRVCULPSXEU-RWORTQBESA-N | C22H26O11 | M489.139T423.333 |  |  |  |
| (2S,3R,4S,5S,6R)-2-[3-hydroxy-5-[(Z)-2-(4-hydroxyphenyl)ethenyl]phenoxy]-6-(hydroxymethyl)oxane-3,4,5-triol | 0.918710769 | HSTZMXCBWJGKHG-BUFXCDORSA-N | C20H22O8 | M413.121T682.407 |  |  |  |
| Acetylvanillin | 0.917280154 | PZSJOBKRSVRODF-UHFFFAOYSA-N | C10H10O4 | M195.065T343.330 |  |  |  |
| Isofraxidin | 0.913582846 | HOEVRHHMDJKUMZ-UHFFFAOYSA-N | C11H10O5 | M223.058T116.410 |  |  |  |
| Chelerythrine chloride | 0.913289 | WEEFNMFMNMASJY-UHFFFAOYSA-M | C21H18NO4+.Cl- | M348.124T399.762 |  |  |  |
| Wilforlide A | 0.909314846 | HHQJBWYXBWOFJY-YLXTXNMFSA-N | C30H46O3 | M455.353T680.631 |  |  |  |
| 2,3-dihydroxypropyl hexadecanoate | 0.905428231 | QHZLMUACJMDIAE-UHFFFAOYSA-N | C19H38O4 | M313.274T968.746 |  |  |  |
| Kaempferol-3-O-glucoside-6''-p-coumaroyl | 0.904397692 | DVGGLGXQSFURLP-VWMSDXGPSA-N | C30H26O13 | M595.144T447.745 |  |  |  |
| Progesterone | 0.902466308 | RJKFOVLPORLFTN-LEKSSAKUSA-N | C21H30O2 | M315.232T660.986 |  |  |  |
| Lycorine | 0.901708154 | XGVJWXAYKUHDOO-DANNLKNASA-N | C16H17NO4 | M288.123T231.355 |  |  |  |
| 7-methoxy-9,10-dihydrophenanthrene-2,5-diol | 0.901069308 | RDKDIPDDUFMMMT-UHFFFAOYSA-N | C15H14O3 | M260.128T342.768 |  |  |  |
| Glycitin | 0.900594 | OZBAVEKZGSOMOJ-MIUGBVLSSA-N | C22H22O10 | M447.129T497.211 |  |  |  |
| (4S,5Z,6S)-4-(2-methoxy-2-oxoethyl)-5-[2-[(E)-3-phenylprop-2-enoyl]oxyethylidene]-6-[(2S,3R,4S,5S,6R)-3,4,5-trihydroxy-6-(hydroxymethyl)oxan-2-yl]oxy-4H-pyran-3-carboxylic acid | 0.900558769 | FKBUODICGDOIGB-UHFFFAOYSA-N | C15H20O3 | M266.174T544.696 |  |  |  |
| 6-[[(3S,6aR,6bS,8aS,14bR)-4,4,6a,6b,11,11,14b-heptamethyl-8a-[3,4,5-trihydroxy-6-(hydroxymethyl)oxan-2-yl]oxycarbonyl-1,2,3,4a,5,6,7,8,9,10,12,12a,14,14a-tetradecahydropicen-3-yl]oxy]-3,5-dihydroxy-4-[3,4,5-trihydroxy-6-(hydroxymethyl)oxan-2-yl]oxyoxane-2-carboxylic acid | 0.899363769 | QZMAEZWZCGBZFK-UOWPQPKISA-N | C48H76O19 | M995.456T742.966 |  |  |  |
| Sinomenine | 0.898559308 | INYYVPJSBIVGPH-QHRIQVFBSA-N | C19H23NO4 | M330.170T164.178 |  |  |  |
| judaicin (eudesmane naphthofuran) | 0.897474308 | NGPDZEACIWDCKX-WUDKWMPASA-N | C15H20O4 | M265.144T185.367 |  |  |  |
| Menadione | 0.896618154 | MJVAVZPDRWSRRC-UHFFFAOYSA-N | C11H8O2 | M173.060T221.116 |  |  |  |
| Corticosterone | 0.895360154 | OMFXVFTZEKFJBZ-HJTSIMOOSA-N | C21H30O4 | M347.219T771.352 |  |  |  |
| Tetrahydropiperine | 0.893908462 | APZYKUZPJCQGPP-UHFFFAOYSA-N | C17H23NO3 | M290.175T663.200 |  |  |  |
| 5-Hydroxymethylfurfural | 0.891333692 | NOEGNKMFWQHSLB-UHFFFAOYSA-N | C6H6O3 | M127.039T85.288 |  |  |  |
| Isopeonol | 0.889648154 | XPHIPEXPAGCEBM-UHFFFAOYSA-N | C9H10O3 | M167.071T124.625 |  |  |  |
| Maltol | 0.887198077 | XPCTZQVDEJYUGT-UHFFFAOYSA-N | C6H6O3 | M127.039T127.856 |  |  |  |
| 3-{(E)-2-[(1R,4aS,5R,6R,8aR)-6-Hydroxy-5-(hydroxymethyl)-5,8a-dimethyl-2-methylenedecahydro-1-naphthalenyl]vinyl}-2(5H)-furanone | 0.884743231 | XMJAJFVLHDIEHF-CRBRZBHVSA-N | C20H28O4 | M333.204T737.849 |  |  |  |
| Phenylalanine | 0.882916077 | COLNVLDHVKWLRT-UHFFFAOYSA-N | C9H11NO2 | M166.086T81.703 |  |  |  |
| Caffeic acid | 0.881186231 | QAIPRVGONGVQAS-DUXPYHPUSA-N | C9H8O4 | M181.049T361.588 |  |  |  |
| L-Tryptophan | 0.880896923 | QIVBCDIJIAJPQS-VIFPVBQESA-N | C11H12N2O2 | M205.097T144.722 |  |  |  |
| Chlorogenic Acid | 0.880871231 | CWVRJTMFETXNAD-JUHZACGLSA-N | C16H18O9 | M355.102T189.401 |  |  |  |
| Glycocholic acid | 0.880429538 | RFDAIACWWDREDC-FRVQLJSFSA-N | C26H43NO6 | M466.311T849.612 |  |  |  |
| Loganin | 0.880357308 | AMBQHHVBBHTQBF-UOUCRYGSSA-N | C17H26O10 | M408.186T265.996 |  |  |  |
| Curcumol | 0.879797231 | QRMPRVXWPCLVNI-XIQJJJERSA-N | C15H24O2 | M237.185T612.136 |  |  |  |
| Poststerone | 0.879596 | VNLQNGYIXVTQRR-BLMSIPRSSA-N | C21H30O5 | M363.214T698.070 |  |  |  |
| Phenethylacetate | 0.879498154 | MDHYEMXUFSJLGV-UHFFFAOYSA-N | C10H12O2 | M165.091T849.612 |  |  |  |
| [(2R,3R,4S,5R,6R)-6-[2-(3,4-dihydroxyphenyl)ethoxy]-3,5-dihydroxy-4-[(3R,4R,5R,6S)-3,4,5-trihydroxy-6-methyloxan-2-yl]oxyoxan-2-yl]methyl (E)-3-(3,4-dihydroxyphenyl)prop-2-enoate | 0.879035769 | FNMHEHXNBNCPCI-KAUJWNRDSA-N | C29H36O15 | M625.214T361.588 |  |  |  |
| testolactone | 0.879002 | BPEWUONYVDABNZ-WFZCBACDSA-N | C19H24O3 | M301.177T666.095 |  |  |  |
| Enoxolone | 0.878766231 | MPDGHEJMBKOTSU-YKLVYJNSSA-N | C30H46O4 | M471.347T771.045 |  |  |  |
| Isoastragaloside I | 0.878227769 | HVPKALQHGQMJER-XOUPSZAESA-N | C45H72O16 | M869.487T660.986 |  |  |  |
| L-2,3-DIAMINOPROPIONIC ACID | 0.876829231 | PECYZEOJVXMISF-UHFFFAOYSA-N | C3H8N2O2 | M105.070T265.634 |  |  |  |
| Demethyleneberberine | 0.873769 | HVTCKKMWZDDWOY-UHFFFAOYSA-O | C19H18NO4+ | M324.124T332.154 |  |  |  |
| Norboldine | 0.872654538 | KYVJVURXKAZJRK-LBPRGKRZSA-N | C18H19NO4 | M314.139T238.039 |  |  |  |
| Biochanin-7-O-glucoside | 0.871169769 | LFEUICHQZGNOHD-UHFFFAOYSA-N | C22H22O10 | M447.129T341.757 |  |  |  |
| Isoxanthohumol | 0.870758385 | YKGCBLWILMDSAV-UHFFFAOYSA-N | C21H22O5 | M355.150T55.176 |  |  |  |
| Daidzein | 0.870750308 | ZQSIJRDFPHDXIC-UHFFFAOYSA-N | C15H10O4 | M255.066T15.062 |  |  |  |
| Phthalic anhydride | 0.870426846 | LGRFSURHDFAFJT-UHFFFAOYSA-N | C8H4O3 | M149.023T809.898 |  |  |  |
| Lindenenol | 0.870268923 | XRDJYSVGPBJZSG-PSDLAXTLSA-N | C15H18O2 | M231.138T651.229 |  |  |  |
| Isochlorogenic acid B | 0.870063 | UFCLZKMFXSILNL-BBLPPJRLSA-N | C25H24O12 | M517.135T470.195 |  |  |  |
| Trigonelline HCl | 0.869949538 | TZSYLWAXZMNUJB-UHFFFAOYSA-N | C7H7NO2.HCl | M138.055T168.383 |  |  |  |
| 2-Hydroxyacetophenone | 0.869627231 | ZWVHTXAYIKBMEE-UHFFFAOYSA-N | C8H8O2 | M137.060T265.996 |  |  |  |
| Erucamide | 0.868783154 | UAUDZVJPLUQNMU-KTKRTIGZSA-N | C22H43NO | M338.342T1105.850 |  |  |  |
| Loliolide | 0.867506923 | XEVQXKKKAVVSMW-UHFFFAOYSA-N | C11H16O3 | M197.117T361.588 |  |  |  |
| 7,8-Dihydroxyflavone | 0.866496615 | COCYGNDCWFKTMF-UHFFFAOYSA-N | C15H10O4 | M255.066T25.773 |  |  |  |
| (2S,3S)-2-(3,4-dihydroxyphenyl)-3,5,7-trihydroxy-6-methyl-2,3-dihydrochromen-4-one | 0.866020538 | KPCWWZLBHGSXPW-CVEARBPZSA-N | C16H14O7 | M319.081T423.850 |  |  |  |
| Oxypeucedanin hydrate | 0.865584077 | PEWFWDOPJISUOK-CYBMUJFWSA-N | C16H16O6 | M305.102T471.331 |  |  |  |
| Veraguensin | 0.864614077 | JLJAVUZBHSLLJL-GKHNXXNSSA-N | C22H28O5 | M373.201T726.251 |  |  |  |
| Homoplantaginin | 0.862438692 | GCLAFEGUXXHIFT-IWLDQSELSA-N | C22H22O11 | M463.125T390.935 |  |  |  |
| Maslinic acid | 0.861748615 | MDZKJHQSJHYOHJ-UHFFFAOYSA-N | C30H48O4 | M473.361T607.261 |  |  |  |
| Boldine | 0.860768462 | LZJRNLRASBVRRX-UHFFFAOYSA-N | C19H21NO4 | M328.155T313.780 |  |  |  |
| Isobutyl 4-hydroxybenzoate | 0.859672692 | XPJVKCRENWUEJH-UHFFFAOYSA-N | C11H14O3 | M195.101T546.142 |  |  |  |
| Di(2-ethylhexyl)phthalate (DEHP) | 0.858965538 | BJQHLKABXJIVAM-UHFFFAOYSA-N | C24H38O4 | M391.284T1095.850 |  |  |  |
| Azuleno[6,5-b]furan-2,6(3H,4H)-dione, 3a,7,7a,8,9,9a-hexahydro-5,8-dimethyl-3-methylene-, (3aR,7aS,8S,9aR)- | 0.857854615 | UQNONRHPSCIIJO-SKVKQCJPSA-N | C15H18O3 | M247.133T449.231 |  |  |  |
| Scopoletin | 0.857014769 | RODXRVNMMDRFIK-UHFFFAOYSA-N | C10H8O4 | M193.049T321.350 |  |  |  |
| aucubin | 0.850702923 | RJWJHRPNHPHBRN-FKVJWERZSA-N | C15H22O9 | M347.133T212.005 |  |  |  |
| L-Anserine nitrate salt | 0.849871692 | MYYIAHXIVFADCU-QMMMGPOBSA-N | C10H16N4O3 | M241.130T58.581 |  |  |  |
| Dipropylphthalate | 0.848120769 | MQHNKCZKNAJROC-UHFFFAOYSA-N | C14H18O4 | M251.126T502.849 |  |  |  |
| Meperidine | 0.847328615 | XADCESSVHJOZHK-UHFFFAOYSA-N | C15H21NO2 | M248.164T525.321 |  |  |  |
| Liquidambaric acid | 0.846958385 | SLJTWDNVZKIDAU-SVAFSPIFSA-N | C30H46O3 | M455.349T853.860 |  |  |  |
| (2R,3R,4S,5S,6R)-2-[(Z)-hex-3-enoxy]-6-(hydroxymethyl)oxane-3,4,5-triol | 0.846587692 | OZIPFYKAIOOVEJ-ODWUMMNUSA-N | C12H22O6 | M285.131T382.492 |  |  |  |
| Senkyunolide A | 0.843802846 | ZPIKVDODKLJKIN-NSHDSACASA-N | C12H16O2 | M193.122T749.683 |  |  |  |
| beta-Elemonic acid | 0.843386385 | XLPAINGDLCDYQV-UHFFFAOYSA-N | C30H46O3 | M455.352T581.307 |  |  |  |
| Asperphenamate_120258 | 0.841273769 | CVULDJMCSSACEO-VMPREFPWSA-N | C32H30N2O4 | M507.227T740.110 |  |  |  |
| Jervine | 0.841243462 | CLEXYFLHGFJONT-DNMILWOZSA-N | C27H39NO3 | M426.301T699.204 |  |  |  |
| Nardosinone | 0.841046308 | KXGHHSIMRWPVQM-JWFUOXDNSA-N | C15H22O3 | M251.164T545.578 |  |  |  |
| Rubrofusarin | 0.840415077 | FPNKCZKRICBAKG-UHFFFAOYSA-N | C15H12O5 | M273.076T399.215 |  |  |  |
| (-)-Sinoacutine | 0.833733538 | GVTRUVGBZQJVTF-YJYMSZOUSA-N | C19H21NO4 | M328.154T197.846 |  |  |  |
| Azelaic acid | 0.830996385 | BDJRBEYXGGNYIS-UHFFFAOYSA-N | C9H16O4 | M189.114T1199.180 |  |  |  |
| ADENOSINE | 0.830494462 | OIRDTQYFTABQOQ-KQYNXXCUSA-N | C10H13N5O4 | M268.104T49.979 |  |  |  |
| Vanillic acid (not validated) | 0.829632077 | QRMZSPFSDQBLIX-UHFFFAOYSA-N | C9H10O4 | M165.055T308.376 |  |  |  |
| Flavanone base +2O, 1MeO | 0.829046154 | DJOJDHGQRNZXQQ-UHFFFAOYSA-N | C16H14O5 | M287.092T737.338 |  |  |  |
| D(-)-Salicin | 0.828990154 | NGFMICBWJRZIBI-UJPOAAIJSA-N | C13H18O7 | M309.097T412.879 |  |  |  |
| Paeonol | 0.827383154 | UILPJVPSNHJFIK-UHFFFAOYSA-N | C9H10O3 | M167.071T221.612 |  |  |  |
| 1,7-bis(4-hydroxyphenyl)heptan-3-one | 0.827047385 | QUHYUSAHBDACNG-UHFFFAOYSA-N | C19H22O3 | M337.118T287.226 |  |  |  |
| Zizyberanalic acid | 0.825402923 | SLWJVQQNDGLXTK-UHFFFAOYSA-N | C30H46O4 | M453.336T654.916 |  |  |  |
| L-Stepholidine | 0.825026 | JKPISQIIWUONPB-HNNXBMFYSA-N | C19H21NO4 | M328.155T291.636 |  |  |  |
| Perillartine | 0.824353462 | XCOJIVIDDFTHGB-UHFFFAOYSA-N | C10H15NO | M166.123T210.012 |  |  |  |
| Timosaponin A-III | 0.820187 | MMTWXUQMLQGAPC-HRSJJQGESA-N | C39H64O13 | M741.442T1605.640 |  |  |  |
| 2-[3-ethenyl-5-methoxycarbonyl-2-[(2S,3R,4S,5S,6R)-3,4,5-trihydroxy-6-(hydroxymethyl)oxan-2-yl]oxy-3,4-dihydro-2H-pyran-4-yl]acetic acid | 0.819742769 | MQLSOVRLZHTATK-WNCYHATQSA-N | C17H24O11 | M427.121T174.818 |  |  |  |
| Camphor | 0.818657923 | DSSYKIVIOFKYAU-UHFFFAOYSA-N | C10H16O | M153.127T391.373 |  |  |  |
| KOBUSONE | 0.817910846 | UETZJEZFLKASPR-UZWIWUQPSA-N | C14H22O2 | M240.196T549.428 |  |  |  |
| 6,7,8-trimethoxychromen-2-one | 0.815864385 | RAYQKHLZHPFYEJ-UHFFFAOYSA-N | C12H12O5 | M237.076T411.755 |  |  |  |
| 2,6-Dimethoxyquinone | 0.812693615 | OLBNOBQOQZRLMP-UHFFFAOYSA-N | C8H8O4 | M186.076T1597.060 |  |  |  |
| Coumarin | 0.812004231 | ZYGHJZDHTFUPRJ-UHFFFAOYSA-N | C9H6O2 | M147.044T386.227 |  |  |  |
| Methyl-3-hydroxybutyric acid | 0.807646385 | VEXDRERIMPLZLU-UHFFFAOYSA-N | C5H10O3 | M119.073T151.477 |  |  |  |
| 3,4-Dimethoxybenzaldehyde | 0.803611769 | WJUFSDZVCOTFON-UHFFFAOYSA-N | C9H10O3 | M167.070T268.336 |  |  |  |
| (1aS,10aR)-1a,5,9-Trimethyl-1a,3,6,10a-tetrahydrooxireno[4,5]cyclodeca[1,2-b]furan-10(2H)-one | 0.801189231 | CVIVANCKIBYAOP-WSQYCBKMSA-N | C15H18O3 | M229.122T580.447 |  |  |  |
| N-Methyl-2-pyrrolidone | 0.800789 | SECXISVLQFMRJM-UHFFFAOYSA-N | C5H9NO | M100.076T953.648 |  |  |  |
| Dexamethasone | 0.800478077 | UREBDLICKHMUKA-UHFFFAOYSA-N | C22H29FO5 | M393.209T271.624 |  |  |  |
| 20-Hydroxyecdysone 20,22-acetonide | 0.798044462 | GXNNYSDWRVKVJY-VUYJMULXSA-N | C30H48O7 | M521.344T880.034 |  |  |  |
| alpha-Boswellic acid | 0.797997769 | BZXULBWGROURAF-IKNLXHIFSA-N | C30H48O3 | M457.368T569.433 |  |  |  |
| [(1S,4aS,5R,7S)-4a,5-dihydroxy-7-methyl-1-[(2S,3R,4S,5S,6R)-3,4,5-trihydroxy-6-(hydroxymethyl)oxan-2-yl]oxy-1,5,6,7a-tetrahydrocyclopenta[c]pyran-7-yl] (E)-3-(4-hydroxyphenyl)prop-2-enoate | 0.797258154 | AZKQDXZMKREFDY-LGKDJQOASA-N | C24H30O12 | M533.165T488.159 |  |  |  |
| Luteolin 6-C-glucoside 8-C-arabinoside | 0.796701308 | ZLPSOQFIIQIIAX-UHFFFAOYSA-N | C27H30O16 | M611.161T213.128 |  |  |  |
| 12-oxo-phytodienoic acid | 0.795838231 | PMTMAFAPLCGXGK-UHFFFAOYSA-N | C18H28O3 | M293.211T538.894 |  |  |  |
| Carveol | 0.792514538 | BAVONGHXFVOKBV-UHFFFAOYSA-N | C10H16O | M135.117T364.851 |  |  |  |
| Ferulic acid | 0.792463231 | KSEBMYQBYZTDHS-HWKANZROSA-N | C10H10O4 | M195.065T326.779 |  |  |  |
| β-Gentiobiose | 0.790628692 | DLRVVLDZNNYCBX-CQUJWQHSSA-N | C12H22O11 | M365.105T43.705 |  |  |  |
| Isoeugenol acetate | 0.788514846 | IUSBVFZKQJGVEP-SNAWJCMRSA-N | C12H14O3 | M207.101T737.338 |  |  |  |
| Glutathione | 0.786447923 | RWSXRVCMGQZWBV-UHFFFAOYSA-N | C10H17N3O6S | M308.091T588.035 |  |  |  |
| Sinapoyl aldehyde | 0.786246462 | CDICDSOGTRCHMG-UHFFFAOYSA-N | C11H12O4 | M209.081T570.590 |  |  |  |
| Benzenepropanamide, N-[2-(acetyloxy)-1-(phenylmethyl)ethyl]-alpha-(benzoylamino)- | 0.784108 | VZPAURMDJZOGHU-UHFFFAOYSA-N | C27H28N2O4 | M445.212T671.708 |  |  |  |
| Epiberberine | 0.778298615 | FPJQGFLUORYYPE-UHFFFAOYSA-N | C20H18NO4+ | M336.123T341.795 |  |  |  |
| swertiamarin | 0.777087385 | HEYZWPRKKUGDCR-QBXMEVCASA-N | C16H22O10 | M397.110T101.413 |  |  |  |
| beta-Asarone | 0.775874231 | RKFAZBXYICVSKP-WAYWQWQTSA-N | C12H16O3 | M209.117T707.125 |  |  |  |
| Ferulaldehyde | 0.774827462 | DKZBBWMURDFHNE-NSCUHMNNSA-N | C10H10O3 | M179.070T315.451 |  |  |  |
| HYDRASTINE (1R, 9S) | 0.774388769 | JZUTXVTYJDCMDU-MOPGFXCFSA-N | C21H21NO6 | M384.145T372.736 |  |  |  |
| (3Z,6E)-2,2,6-trimethyl-12-oxabicyclo[8.2.1]trideca-3,6,10(13)-triene-5,11-dione | 0.77293 | CAHQQYHQUHYOGU-KLQKCIPISA-N | C15H18O3 | M247.133T353.273 |  |  |  |
| methyl (1S,4aR,7aR)-4a-hydroxy-7-(hydroxymethyl)-1-[(2S,3R,4S,5S,6R)-3,4,5-trihydroxy-6-(hydroxymethyl)oxan-2-yl]oxy-5,7a-dihydro-1H-cyclopenta[c]pyran-4-carboxylate | 0.770673538 | LDBMLOLBWUOZGG-DOFVRBEMSA-N | C17H24O11 | M443.096T179.317 |  |  |  |
| Bicuculline | 0.770036462 | IYGYMKDQCDOMRE-ZWKOTPCHSA-N | C20H17NO6 | M368.113T340.560 |  |  |  |
| Sinomenine HCl | 0.769636385 | YMEVIMJAUHZFMW-VUIDNZEBSA-N | C19H23NO4.HCl | M330.169T295.309 |  |  |  |
| (4Z,7Z)-5,9,9-Trimethyl-11-oxabicyclo[8.2.1]trideca-1(13),4,7-triene-6,12-dione | 0.769418692 | CAHQQYHQUHYOGU-JCPYGFQFSA-N | C15H18O3 | M264.159T466.964 |  |  |  |
| Melibiose | 0.769170308 | DLRVVLDZNNYCBX-ZZFZYMBESA-N | C12H22O11 | M360.151T46.989 |  |  |  |
| 3,8a-Dihydroxy-5-isopropylidene-3,8-dimethyl-2,3,3a,4,5,8a-hexahydro-6(1H)-azulenone | 0.765465308 | IXQJBPRUTQTCMW-UHFFFAOYSA-N | C15H22O3 | M233.153T609.582 |  |  |  |
| Isophorone | 0.765427846 | HJOVHMDZYOCNQW-UHFFFAOYSA-N | C9H14O | M139.112T318.182 |  |  |  |
| Goniothalenol | 0.764136 | ZKIRVBNLJKGIEM-UHFFFAOYSA-N | C13H12O4 | M250.108T78.615 |  |  |  |
| Soyasapogenol B base + O-HexA-Pen-dHex | 0.762498231 | IBZLICPLPYSFNZ-UHFFFAOYSA-N | C47H76O17 | M913.506T891.412 |  |  |  |
| Artemisinic acid | 0.757125846 | PLQMEXSCSAIXGB-SAXRGWBVSA-N | C15H22O2 | M235.169T480.742 |  |  |  |
| Cantharidin | 0.754962308 | DHZBEENLJMYSHQ-XCVPVQRUSA-N | C10H12O4 | M197.081T169.305 |  |  |  |
| 2-Cyclohexen-1-one, 4-hydroxy-4-(3-hydroxybutyl)-3,5,5-trimethyl- | 0.752740385 | CWOFGGNDZOPNFG-UHFFFAOYSA-N | C13H22O3 | M209.154T468.527 |  |  |  |
| (-)-12-hydroxyjasmonic acid | 0.752458 | RZGFUGXQKMEMOO-BSANDHCLSA-N | C12H18O4 | M249.110T479.106 |  |  |  |
| retronecine | 0.749793769 | HJSJELVDQOXCHO-HTQZYQBOSA-N | C8H13NO2 | M156.102T170.755 |  |  |  |
| Isoreserpiline, citrate | 0.749175538 | PEXDQBIIYNZYBT-UHFFFAOYSA-N | C29H36N2O12 | M413.209T812.113 |  |  |  |
| Quillaic acid | 0.745921308 | MQUFAARYGOUYEV-UAWZMHPWSA-N | C30H46O5 | M487.342T748.544 |  |  |  |
| Nuciferine | 0.745887923 | ORJVQPIHKOARKV-OAHLLOKOSA-N | C19H21NO2 | M296.165T440.134 |  |  |  |
| ESTRADIOL | 0.740287923 | VOXZDWNPVJITMN-ZBRFXRBCSA-N | C18H24O2 | M273.185T606.116 |  |  |  |
| 7-Methoxycoumarin | 0.739538462 | LIIALPBMIOVAHH-UHFFFAOYSA-N | C10H8O3 | M177.054T348.265 |  |  |  |
| (2R,3S,4S,5R,6R)-2-[[(2R,3R,4R,5S)-3,4-dihydroxy-5-(hydroxymethyl)oxolan-2-yl]oxymethyl]-6-[(2E)-3,7-dimethylocta-2,6-dienoxy]oxane-3,4,5-triol | 0.738546308 | AWDKYYYAAQQLEF-XRSPUIAPSA-N | C21H36O10 | M466.266T175.898 |  |  |  |
| Tazettine | 0.734886769 | YLWAQARRNQVEHD-UHFFFAOYSA-N | C18H21NO5 | M332.150T102.371 |  |  |  |
| Norisoboldine | 0.733921538 | HORZNQYQXBFWNZ-LBPRGKRZSA-N | C18H19NO4 | M314.138T194.839 |  |  |  |
| plumieride | 0.733094769 | AOPMSFXOYJXDNJ-IRFSQMTFSA-N | C21H26O12 | M471.150T377.098 |  |  |  |
| N-(1-hydroxy-3-phenylpropan-2-yl)benzamide | 0.728534462 | RFYNAVYPYXLVOM-UHFFFAOYSA-N | C16H17NO2 | M256.134T498.904 |  |  |  |
| Cyclo(leucylprolyl) | 0.723375923 | SZJNCZMRZAUNQT-UHFFFAOYSA-N | C11H18N2O2 | M211.144T308.938 |  |  |  |
| Santonin | 0.721665923 | XJHDMGJURBVLLE-BOCCBSBMSA-N | C15H18O3 | M247.133T181.424 |  |  |  |
| 5,7-dihydroxy-2-(4-hydroxyphenyl)-8-[3,4,5-trihydroxy-6-(hydroxymethyl)oxan-2-yl]-6-(3,4,5-trihydroxyoxan-2-yl)chromen-4-one | 0.720117385 | OVMFOVNOXASTPA-UHFFFAOYSA-N | C26H28O14 | M565.157T311.731 |  |  |  |
| Allocryptopine | 0.719287923 | HYBRYAPKQCZIAE-UHFFFAOYSA-N | C21H23NO5 | M370.165T330.082 |  |  |  |
| 3-Hydroxy-5-isopropylidene-3,8-dimethyl-2,3,3a,4,5,8a-hexahydro-6(1H)-azulenone | 0.717078692 | RHBOHEXDGUVIIY-UHFFFAOYSA-N | C15H22O2 | M217.159T536.186 |  |  |  |
| [(1S,6S,7R)-6-acetyloxy-1-(3-methylbutanoyloxy)spiro[4a,5,6,7a-tetrahydro-1H-cyclopenta[c]pyran-7,2'-oxirane]-4-yl]methyl 3-methylbutanoate | 0.716904462 | PHHROXLDZHUIGO-VKLNETFXSA-N | C22H32O8 | M425.215T871.884 |  |  |  |
| Phenylpropanolamine | 0.715055538 | DLNKOYKMWOXYQA-UHFFFAOYSA-N | C9H13NO | M152.107T609.269 |  |  |  |
| Diosbulbin B | 0.713581615 | QEANLIISUSNNDX-XBHMPIGQSA-N | C19H20O6 | M362.160T523.061 |  |  |  |
| L-(-)-Phenylalanine | 0.713060385 | COLNVLDHVKWLRT-QMMMGPOBSA-N | C9H11NO2 | M166.086T233.784 |  |  |  |
| (2R,3S,4S,5R,6R)-5-[(2S,3R,4R)-3,4-dihydroxy-4-(hydroxymethyl)oxolan-2-yl]oxy-2-(hydroxymethyl)-6-(2-phenylethoxy)oxane-3,4-diol | 0.712964615 | UWKRNCNWJVCHGZ-DERWZFJFSA-N | C19H28O10 | M434.203T319.245 |  |  |  |
| Homoorientin | 0.712472154 | ODBRNZZJSYPIDI-VJXVFPJBSA-N | C21H20O11 | M449.107T303.800 |  |  |  |
| 3,4-Dihydrocoumarin | 0.710764 | VMUXSMXIQBNMGZ-UHFFFAOYSA-N | C9H8O2 | M149.060T677.308 |  |  |  |
| isosakuranetin | 0.709026385 | HMUJXQRRKBLVOO-AWEZNQCLSA-N | C16H14O5 | M287.092T496.668 |  |  |  |
| Pesticide3_Propoxur_C11H15NO3_Baygon | 0.708252385 | ISRUGXGCCGIOQO-UHFFFAOYSA-N | C11H15NO3 | M210.113T191.935 |  |  |  |
| Astragaloside II | 0.703127769 | AYWNHWGQTMCQIV-PENCHUSISA-N | C43H70O15 | M827.479T626.774 |  |  |  |
| Kaempferol 3-O-sophoroside | 0.702107231 | LKZDFKLGDGSGEO-UJECXLDQSA-N | C27H30O16 | M611.161T247.552 |  |  |  |
| (+)-Corynoline | 0.701840615 | IQUGPRHKZNCHGC-TYPHKJRUSA-N | C21H21NO5 | M368.149T346.117 |  |  |  |
| (3aR,4R,6aR,8S,9aR,9bR)-4,8-Dihydroxy-3,6,9-tris(methylene)decahydroazuleno[4,5-b]furan-2(3H)-one | 0.701040231 | VPRPYNVJJXOFKZ-BSCCGWMCSA-N | C15H18O4 | M245.118T509.559 |  |  |  |
| Asiatic acid | 0.698261462 | JXSVIVRDWWRQRT-UYDOISQJSA-N | C30H48O5 | M511.339T674.398 |  |  |  |
| Picrotin | 0.697873615 | RYEFFICCPKWYML-QCGISDTRSA-N | C15H18O7 | M311.103T325.092 |  |  |  |
| Papaverine HCl | 0.695404462 | UOTMYNBWXDUBNX-UHFFFAOYSA-N | C20H21NO4.HCl | M340.155T664.329 |  |  |  |
| Gibberellin A4&A7 | 0.695287692 | SEEGHKWOBVVBTQ-UKJRIFTCSA-N | C19H22O5 | M369.108T360.330 |  |  |  |
| Ethylparaben | 0.694191538 | NUVBSKCKDOMJSU-UHFFFAOYSA-N | C9H10O3 | M167.070T439.575 |  |  |  |
| Methyl vanillate | 0.693227923 | BVWTXUYLKBHMOX-UHFFFAOYSA-N | C9H10O4 | M182.985T1625.130 |  |  |  |
| Histamine | 0.691658923 | NTYJJOPFIAHURM-UHFFFAOYSA-N | C5H9N3 | M112.087T46.065 |  |  |  |
| Dehydronuciferin | 0.691391077 | JBGSWIBJAGBGOP-UHFFFAOYSA-N | C19H19NO2 | M294.149T431.444 |  |  |  |
| Corydaline | 0.690514462 | VRSRXLJTYQVOHC-YEJXKQKISA-N | C22H27NO4 | M370.201T516.342 |  |  |  |
| Veratramine | 0.689544923 | MALFODICFSIXPO-KFKQDBFTSA-N | C27H39NO2 | M410.306T977.924 |  |  |  |
| 4-Hydroxycoumarin | 0.688523692 | VXIXUWQIVKSKSA-UHFFFAOYSA-N | C9H6O3 | M163.039T361.588 |  |  |  |
| 3-(4-HYDROXYPHENYL)PYRUVATE | 0.687495615 | KKADPXVIOXHVKN-UHFFFAOYSA-N | C9H8O4 | M181.049T211.840 |  |  |  |
| 3-(2-Hydroxy-3,4-dimethoxyphenyl)-7-chromanol | 0.678878923 | NQRBAPDEZYMKFL-UHFFFAOYSA-N | C17H18O5 | M320.149T337.892 |  |  |  |
| Ursolic acid | 0.675678462 | WCGUUGGRBIKTOS-GPOJBZKASA-N | C30H48O3 | M439.358T544.477 |  |  |  |
| Pyridoxine | 0.67517 | LXNHXLLTXMVWPM-UHFFFAOYSA-N | C8H11NO3 | M170.081T49.979 |  |  |  |
| Camptothecin | 0.674309308 | VSJKWCGYPAHWDS-FQEVSTJZSA-N | C20H16N2O4 | M349.118T402.319 |  |  |  |
| acanthoside B | 0.674182923 | WEKCEGQSIIQPAQ-IRBNZIFYSA-N | C28H36O13 | M603.205T388.192 |  |  |  |
| 3,4-dihydroxybenzoic acid | 0.670665923 | YQUVCSBJEUQKSH-UHFFFAOYSA-N | C7H6O4 | M155.034T194.048 |  |  |  |
| 2-Methoxy-4-{(2S,3R)-7-methoxy-3-methyl-5-[(1E)-1-propen-1-yl]-2,3-dihydro-1-benzofuran-2-yl}phenol | 0.667920615 | ITDOFWOJEDZPCF-SZJIHULOSA-N | C20H22O4 | M327.159T374.269 |  |  |  |
| Moluccanin | 0.665773615 | GBLZBLJGCQTQMB-PXNSSMCTSA-N | C20H18O8 | M387.106T540.540 |  |  |  |
| 2H-Cyclohepta[b]furan-2-one, 3,3a,4,7,8,8a-hexahydro-6-(3-hydroxybutyl)-7-methyl-3-methylene- | 0.665635154 | KQEADOUDJYBGFC-UHFFFAOYSA-N | C15H22O3 | M233.151T1593.620 |  |  |  |
| Spiro[7H-cyclohepta[b]furan-7,2'(5'H)-furan]-2,5'(3H)-dione, octahydro-8-hydroxy-6,8-dimethyl-3-methylene-, (3aS,6S,7R,8aR)- | 0.663321462 | IRPFOXRBPHCCTG-CZLFTNFUSA-N | C15H20O5 | M263.128T487.685 |  |  |  |
| Higenamine | 0.662630462 | WZRCQWQRFZITDX-UHFFFAOYSA-N | C16H17NO3 | M272.128T507.887 |  |  |  |
| rosmarinic acid | 0.660248769 | DOUMFZQKYFQNTF-WUTVXBCWSA-N | C18H16O8 | M361.092T310.624 |  |  |  |
| (2S,3S,4S,5R,6R)-6-[[(3S,4R,6aR,6bS,8aS,14bR)-4-(hydroxymethyl)-4,6a,6b,11,11,14b-hexamethyl-8a-[(2S,3R,4S,5S,6R)-3,4,5-trihydroxy-6-(hydroxymethyl)oxan-2-yl]oxycarbonyl-1,2,3,4a,5,6,7,8,9,10,12,12a,14,14a-tetradecahydropicen-3-yl]oxy]-3,4,5-trihydroxyoxane-2-carboxylic acid | 0.655646077 | RZQHWSDMLZHIRN-MCTPBCADSA-N | C42H66O15 | M849.408T601.665 |  |  |  |
| Salsolinol | 0.655228692 | IBRKLUSXDYATLG-UHFFFAOYSA-N | C10H13NO2 | M180.102T50.272 |  |  |  |
| Dehydrodiisoeugenol | 0.647367923 | ITDOFWOJEDZPCF-UHFFFAOYSA-N | C20H22O4 | M327.159T582.666 |  |  |  |
| Isomucronulatol 7-O-glucoside | 0.645203462 | SXHOGLPTLQBGDO-ZVSSUSCDSA-N | C23H28O10 | M465.175T465.905 |  |  |  |
| Phillygenin | 0.642573154 | CPJKKWDCUOOTEW-YJPXFSGGSA-N | C21H24O6 | M373.164T590.806 |  |  |  |
| Tricin | 0.642479923 | HRGUSFBJBOKSML-UHFFFAOYSA-N | C17H14O7 | M331.082T426.613 |  |  |  |
| Soyasapogenol E base + O-HexA-Hex-Hex | 0.642141385 | JTXVTHCLTOUSSL-UHFFFAOYSA-N | C48H76O19 | M957.503T613.834 |  |  |  |
| 8-(2-hydroxy-3-methylbut-3-enyl)-7-methoxychromen-2-one | 0.641670923 | SQSRYWNOKPJENY-UHFFFAOYSA-N | C15H16O4 | M243.101T599.479 |  |  |  |
| (1R,3R,4S,5R)-1,3,4-trihydroxy-5-[(E)-3-(4-hydroxyphenyl)prop-2-enoyl]oxycyclohexane-1-carboxylic acid | 0.638598 | BMRSEYFENKXDIS-QHAYPTCMSA-N | C16H18O8 | M339.107T417.224 |  |  |  |
| 4-Nitrophenol | 0.637759846 | BTJIUGUIPKRLHP-UHFFFAOYSA-N | C6H5NO3 | M140.034T50.272 |  |  |  |
| Noreugenin | 0.636661154 | NCUJRUDLFCGVOE-UHFFFAOYSA-N | C10H8O4 | M193.049T309.500 |  |  |  |
| rhodioloside | 0.634916 | ILRCGYURZSFMEG-RKQHYHRCSA-N | C14H20O7 | M323.111T326.289 |  |  |  |
| Lutl-6-C-Glc | 0.633482308 | PEFNSGRTCBGNAN-QNDFHXLGSA-N | C21H20O11 | M449.107T335.404 |  |  |  |
| AMINOADIPATE | 0.631771538 | OYIFNHCXNCRBQI-BYPYZUCNSA-N | C6H11NO4 | M162.076T45.136 |  |  |  |
| Berberrubine | 0.629894846 | GYFSYEVKFOOLFZ-UHFFFAOYSA-N | C19H15NO4 | M322.107T640.177 |  |  |  |
| Vittatine | 0.629707308 | RPAORVSEYNOMBR-RLCCDNCMSA-N | C16H17NO3 | M272.129T477.976 |  |  |  |
| Dihydrocapsaicin | 0.628682231 | XJQPQKLURWNAAH-UHFFFAOYSA-N | C18H29NO3 | M308.222T484.696 |  |  |  |
| D-PANTOTHENIC ACID | 0.628499923 | GHOKWGTUZJEAQD-ZETCQYMHSA-N | C9H17NO5 | M220.118T93.949 |  |  |  |
| 5'-(furan-3-yl)-4a-hydroxy-4,7-dimethylspiro[5,6,7,8a-tetrahydro-1H-naphthalene-8,3'-oxolane]-2,2'-dione | 0.627341154 | DLHZVTVUHATYAU-UHFFFAOYSA-N | C19H22O5 | M331.155T388.522 |  |  |  |
| Veratric acid | 0.625137231 | DAUAQNGYDSHRET-UHFFFAOYSA-N | C9H10O4 | M183.065T115.253 |  |  |  |
| POTASSIUM SORBATE | 0.624999308 | CHHHXKFHOYLYRE-STWYSWDKSA-M | C6H7KO2 | M113.060T141.611 |  |  |  |
| (2E)-5-(2,3-Dimethyltricyclo[2.2.1.0~2,6~]hept-3-yl)-2-methyl-2-pentenoic acid | 0.624163231 | NZSCHTYUGUVLHG-WEVVVXLNSA-N | C15H22O2 | M235.169T576.333 |  |  |  |
| Demethylcoclaurine hydrochloride | 0.623379692 | SWWQQSDRUYSMAR-UHFFFAOYSA-N | C16H17NO3.HCl | M272.128T261.039 |  |  |  |
| Hyperoside | 0.620234308 | OVSQVDMCBVZWGM-DTGCRPNFSA-N | C21H20O12 | M465.102T340.010 |  |  |  |
| Glabridin | 0.620185923 | LBQIJVLKGVZRIW-ZDUSSCGKSA-N | C20H20O4 | M325.144T599.479 |  |  |  |
| Beta-Caryophyllene Alcohol | 0.61755 | FUQAYSQLAOJBBC-UHFFFAOYSA-N | C15H26O | M240.232T1105.850 |  |  |  |
| Cearoin | 0.617533385 | NFJVELXCUBWAFL-UHFFFAOYSA-N | C14H12O4 | M245.081T371.629 |  |  |  |
| Pogostone | 0.615969154 | AJFJTORMMHWKFW-UHFFFAOYSA-N | C12H16O4 | M225.112T553.356 |  |  |  |
| 3,8-dihydroxy-3,8-dimethyl-5-propan-2-ylidene-1,2,3a,4,7,8a-hexahydroazulen-6-one | 0.613596846 | TXIKNNOOLCGADE-UHFFFAOYSA-N | C15H24O3 | M253.180T473.466 |  |  |  |
| Liquiritigenin | 0.611942538 | FURUXTVZLHCCNA-AWEZNQCLSA-N | C15H12O4 | M257.081T461.467 |  |  |  |
| Protosappanin B | 0.608918462 | QRTYTQTVJQUCEP-INIZCTEOSA-N | C16H16O6 | M322.128T55.176 |  |  |  |
| Astragaloside IV | 0.606711923 | QMNWISYXSJWHRY-CSXKERSZSA-N | C41H68O14 | M785.469T534.079 |  |  |  |
| (2R,3S,4S,5R,6R)-2-(hydroxymethyl)-6-[4-(4-hydroxy-2,6,6-trimethylcyclohexen-1-yl)butan-2-yloxy]oxane-3,4,5-triol | 0.606603154 | QOLJILMTPKQQQA-KWEBYEANSA-N | C19H34O7 | M397.222T479.106 |  |  |  |
| Flavokawain C | 0.605894769 | UXUFMIJZNYXWDX-VMPITWQZSA-N | C17H16O5 | M301.107T533.064 |  |  |  |
| Indigo | 0.605717231 | QQILFGKZUJYXGS-UHFFFAOYSA-N | C16H10O2N2 | M263.082T688.871 |  |  |  |
| Jatrorrhizine | 0.600289769 | MXTLAHSTUOXGQF-UHFFFAOYSA-O | C20H20NO4+ | M338.139T417.224 |  |  |  |
