# Supplementary material for: Experimental verification about treatment of Bu-Shen-Yi-Jing-Fang in Alzheimer’s disease by the analysis of the feasible signaling pathway of network pharmacology
Source: BMC Complement Med Ther. 2024 Jun 8;24:222. doi: 10.1186/s12906-024-04527-w (PMC11162075; doi:10.1186/s12906-024-04527-w)

Fig 5

P=AKT

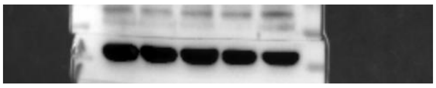

AKT

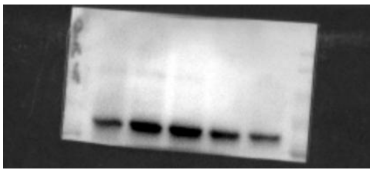

β **-actin**

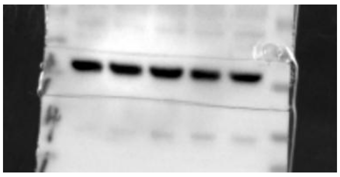

Fig 6

P-AKT

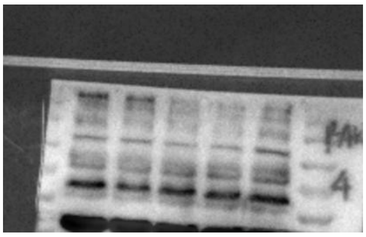

AKT

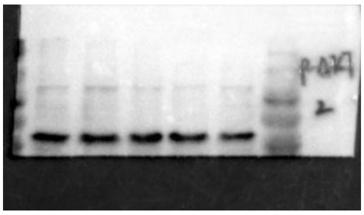

P-GSK3 β

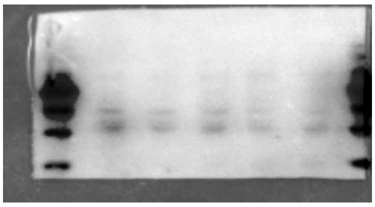

GSK3 β

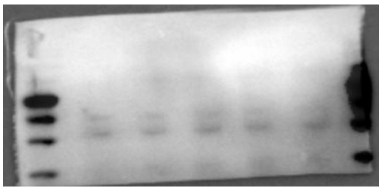

NQO1

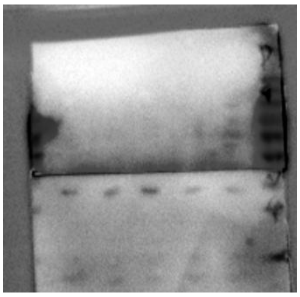

HO-1

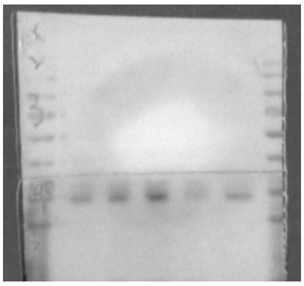

β -actin

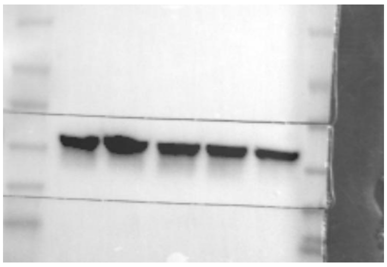

H3

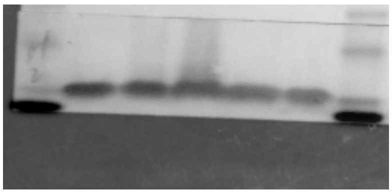

Nuclear-Nrf2

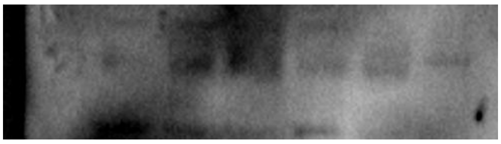

Cytoplasmic-Nrf2

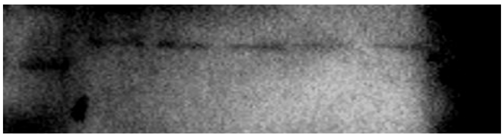

Fig 8

ACSL4

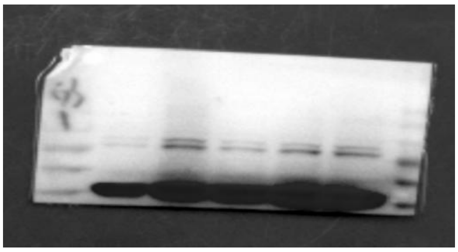

GPX4

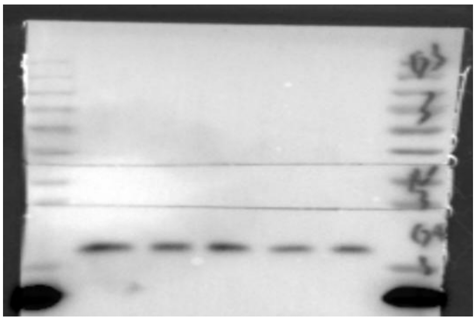

β -actin

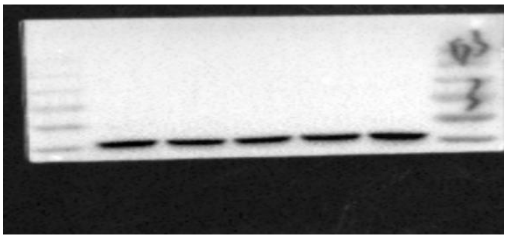

Fig 9

BCL-2

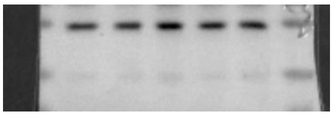

BAX

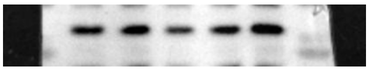

Cytochrome-C

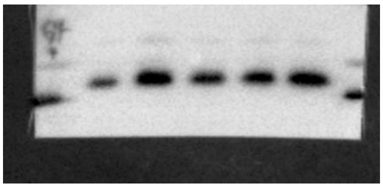

Cleaved caspase 9 /Caspase 9

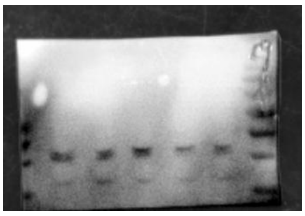

Cleaved Caspase 3 /Caspase 3

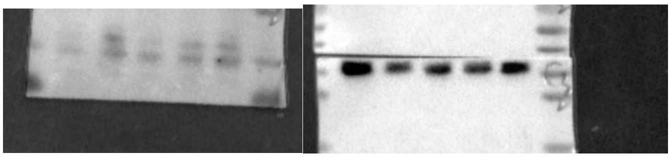

Supplement: Supplementary file 2 — Supplementary Material 2 [file 12906_2024_4527_MOESM2_ESM.pdf]
