# Supplementary material for: Experimental verification about treatment of Bu-Shen-Yi-Jing-Fang in Alzheimer’s disease by the analysis of the feasible signaling pathway of network pharmacology
Source: BMC Complement Med Ther. 2024 Jun 8;24:222. doi: 10.1186/s12906-024-04527-w (PMC11162075; doi:10.1186/s12906-024-04527-w)
Supplement: Supplementary file 3 — Supplementary Material 3 [file 12906_2024_4527_MOESM3_ESM.docx]

| Table1 | | | | | |
| --- | --- | --- | --- | --- | --- |
|  | | | | | |
| KEGG pathway analysis (the Top 20 of pathways) | | | | | |
| Term ID | Description | p value | Observed gene count | Background gene count | Symbols |
| hsa05200 | Pathways in cancer | 2.25113E-68 | 76 | 395 | ABL1,AGTR1,AKT1,XIAP,AR,CCND1,BCL2,BCL2L1,BRAF,CASP3,CASP8,CASP9,CDK4,CHUK,CREBBP,CSF1R,DAPK1,EDNRA,EDNRB,EGFR,EP300,ERBB2,FGF1,FGF2,FGFR1,FGFR2,FOXO1,FLT3,MTOR,GSK3B,HDAC1,HDAC2,HIF1A,HSP90AA1,IGF1R,IKBKB,IL6,CXCL8,ITGA2B,JAK1,JUN,KIT,MDM2,MET,MMP1,MMP2,MMP9,NFKB1,NOS2,NTRK1,PDGFRA,PDGFRB,PGF,PIK3CA,PIK3R1,PPARD,PPARG,PRKACA,PRKCA,MAPK1,MAPK3,MAPK8,MAPK9,MAPK10,MAP2K1,PTGS2,RAF1,RARA,RARB,RELA,RET,STAT3,TGFB1,TGFBR1,TP53,VEGFA |
| hsa04080 | Neuroactive ligand-receptor interaction | 5.12386E-57 | 60 | 277 | ADORA2A,ADORA2B,ADRA1D,ADRA1B,ADRA1A,ADRA2A,ADRA2B,ADRA2C,ADRB1,ADRB2,ADRB3,AGTR1,AGTR2,TSPO,CHRM1,CHRM2,CHRM3,CHRM4,CHRM5,CHRNA3,CHRNA4,CHRNA7,CHRNB2,CHRNB4,CNR1,CTSG,DRD1,DRD2,DRD3,DRD4,DRD5,EDNRA,EDNRB,F2,FPR2,GABRA5,GABRG2,GLRA1,GRIN1,GRIN2B,NR3C1,GRM1,GRM5,HRH1,HRH2,HTR1A,HTR1B,HTR1D,HTR2A,HTR2C,HTR4,HTR6,HTR7,MC4R,MTNR1A,OPRM1,P2RX7,PLG,PRSS1,PTGFR |
| hsa04151 | PI3K-Akt signaling pathway | 7.10679E-46 | 58 | 380 | AKT1,CCND1,BCL2,BCL2L1,CASP9,CDK4,CHRM1,CHRM2,CHUK,CSF1R,EGFR,ERBB2,ERBB4,FGF1,FGF2,FGFR1,FGFR2,FLT1,FLT3,MTOR,GSK3B,GYS1,HSP90AA1,IGF1R,IKBKB,IL2,IL6,INSR,ITGA2B,ITGB3,JAK1,JAK2,JAK3,KDR,KIT,MDM2,MET,NFKB1,NOS3,NTRK1,PDGFRA,PDGFRB,PGF,PIK3CA,PIK3CG,PIK3R1,PPP2CA,PRKCA,MAPK1,MAPK3,MAP2K1,RAF1,RELA,SYK,TEK,TLR4,TP53,VEGFA |
| hsa04020 | Calcium signaling pathway | 4.26914E-44 | 44 | 182 | ADORA2A,ADORA2B,ADRA1D,ADRA1B,ADRA1A,ADRB1,ADRB2,ADRB3,AGTR1,CALM1,CHRM1,CHRM2,CHRM3,CHRM5,CHRNA7,DRD1,DRD5,EDNRA,EDNRB,EGFR,ERBB2,ERBB4,GRIN1,GRM1,GRM5,HRH1,HRH2,HTR2A,HTR2C,HTR4,HTR6,HTR7,MYLK,NOS1,NOS2,NOS3,P2RX7,PDGFRA,PDGFRB,PHKG2,PRKACA,PRKCA,PTGFR,CACNA1G |
| hsa04933 | AGE-RAGE signaling pathway in diabetic complications | 4.6237E-42 | 36 | 107 | AGTR1,AKT1,CCND1,BCL2,CASP3,CDK4,MAPK14,F3,FOXO1,ICAM1,IL6,CXCL8,JAK2,JUN,MMP2,NFKB1,NOS3,SERPINE1,PIK3CA,PIK3R1,PRKCA,PRKCD,PRKCE,PRKCZ,MAPK1,MAPK3,MAPK8,MAPK9,MAPK10,RELA,SELE,STAT3,TGFB1,TGFBR1,TNF,VEGFA |
| hsa05161 | Hepatitis B | 9.66535E-42 | 42 | 177 | AKT1,CCND1,BCL2,BRAF,CASP3,CASP8,CASP9,CDK4,CHUK,CREBBP,MAPK14,EP300,IKBKB,IL6,CXCL8,IRAK1,JAK1,JAK2,JAK3,JUN,MMP9,NFKB1,PIK3CA,PIK3R1,PRKCA,MAPK1,MAPK3,MAPK8,MAPK9,MAPK10,MAP2K1,RAF1,RELA,SRC,STAT3,TGFB1,TGFBR1,TLR4,TNF,TP53,TBK1,IRAK4 |
| hsa04014 | Ras signaling pathway | 1.65463E-39 | 46 | 259 | ABL1,AKT1,BCL2L1,CALM1,CHUK,CSF1R,EGFR,FGF1,FGF2,FGFR1,FGFR2,FLT1,FLT3,GRIN1,GRIN2B,HTR7,IGF1R,IKBKB,IL6,INSR,KDR,KIT,MET,NFKB1,NTRK1,PDGFRA,PDGFRB,PGF,PIK3CA,PIK3R1,PLA2G2A,PLA2G4A,PRKACA,PRKCA,MAPK1,MAPK3,MAPK8,MAPK9,MAPK10,MAP2K1,PTPN11,RAF1,RELA,TEK,VEGFA,TBK1 |
| hsa04210 | Apoptosis | 1.65017E-37 | 36 | 138 | PARP1,AKT1,XIAP,BCL2,BCL2L1,CAPN1,CASP3,CASP6,CASP7,CASP8,CASP9,CHUK,CTSB,CTSD,CTSK,CTSL,IKBKB,JUN,MAP3K5,NFKB1,NTRK1,PIK3CA,PIK3R1,PRF1,MAPK1,MAPK3,MAPK8,MAPK9,MAPK10,MAP2K1,RAF1,RELA,TNF,TNFRSF1A,TP53,EIF2AK3 |
| hsa05215 | Prostate cancer | 2.9434E-37 | 31 | 87 | AKT1,AR,CCND1,BCL2,BRAF,CASP9,CHUK,CREBBP,EGFR,EP300,ERBB2,FGFR1,FGFR2,FOXO1,MTOR,GSK3B,HSP90AA1,IGF1R,IKBKB,MDM2,NFKB1,PDGFRA,PDGFRB,PIK3CA,PIK3R1,MAPK1,MAPK3,MAP2K1,RAF1,RELA,TP53 |
| hsa04024 | cAMP signaling pathway | 1.15834E-35 | 41 | 226 | ADORA2A,ADRB1,ADRB2,AKT1,ATP1A1,BRAF,CALM1,CHRM1,CHRM2,CREBBP,DRD1,DRD2,DRD5,EDNRA,EP300,GRIN1,GRIN2B,HTR1A,HTR1B,HTR1D,HTR4,HTR6,JUN,NFKB1,PDE4A,PDE4D,PIK3CA,PIK3R1,PPARA,PRKACA,PRKCA,MAPK1,MAPK3,MAPK8,MAPK9,MAPK10,MAP2K1,RAF1,RELA,SLC9A1,TNNI3 |
| hsa04015 | Rap1 signaling pathway | 1.68712E-35 | 41 | 228 | ADORA2A,ADORA2B,AKT1,BRAF,CALM1,CNR1,MAPK14,CSF1R,DRD2,EGFR,FGF1,FGF2,FGFR1,FGFR2,FLT1,GRIN1,GRIN2B,IGF1R,IL6,INSR,ITGA2B,ITGAL,ITGB2,ITGB3,KDR,KIT,MET,PDGFRA,PDGFRB,PGF,PIK3CA,PIK3R1,PRKCA,PRKCZ,MAPK1,MAPK3,MAP2K1,RAF1,SRC,TEK,VEGFA |
| hsa05205 | Proteoglycans in cancer | 5.15421E-35 | 40 | 218 | AKT1,CCND1,BRAF,CASP3,MAPK14,CTSL,EGFR,ERBB2,ERBB4,ESR1,FGF2,FGFR1,MTOR,HIF1A,IGF1R,IL6,ITGB3,KDR,MDM2,MET,MMP2,MMP9,PIK3CA,PIK3R1,PLAU,PRKACA,PRKCA,MAPK1,MAPK3,MAP2K1,PTPN11,RAF1,SLC9A1,SRC,STAT3,TGFB1,TLR4,TNF,TP53,VEGFA |
| hsa05212 | Pancreatic cancer | 5.22716E-35 | 27 | 64 | AKT1,CCND1,BCL2L1,BRAF,CASP9,CDK4,CHUK,EGFR,ERBB2,IKBKB,JAK1,NFKB1,PIK3CA,PIK3R1,MAPK1,MAPK3,MAPK8,MAPK9,MAPK10,MAP2K1,RAF1,RELA,STAT3,TGFB1,TGFBR1,TP53,VEGFA |
| hsa05418 | Fluid shear stress and atherosclerosis | 7.57366E-35 | 35 | 148 | AKT1,BCL2,CALM1,CHUK,MAPK14,CTSL,NQO1,HMOX1,HSP90AA1,ICAM1,IKBKB,ITGA2B,ITGB3,JUN,KDR,MAP3K5,MMP2,MMP9,NFE2L2,NFKB1,NOS3,PIK3CA,PIK3R1,PRKCZ,MAPK8,MAPK9,MAPK10,RELA,SELE,SRC,TNF,TNFRSF1A,TP53,VEGFA,GSTO1 |
| hsa01522 | Endocrine resistance | 4.4687E-34 | 30 | 96 | AKT1,CCND1,BCL2,BRAF,CDK4,MAPK14,CYP2D6,EGFR,ERBB2,ESR1,ESR2,MTOR,IGF1R,JUN,MDM2,MMP2,MMP9,PIK3CA,PIK3R1,PRKACA,MAPK1,MAPK3,MAPK8,MAPK9,MAPK10,MAP2K1,RAF1,SRC,TP53,ABCB11 |
| hsa05162 | Measles | 9.04981E-34 | 35 | 158 | AKT1,CCND1,BCL2,BCL2L1,CASP3,CASP8,CASP9,CDK4,CHUK,CSNK2A1,FYN,GSK3B,IKBKB,IL2,IL6,IRAK1,JAK1,JAK2,JAK3,JUN,NFKB1,PIK3CA,PIK3R1,PRKCQ,MAPK8,MAPK9,MAPK10,RELA,STAT3,TLR4,TP53,EIF2AK3,TBK1,IRAK4,TLR9 |
| hsa01521 | EGFR tyrosine kinase inhibitor resistance | 1.13633E-33 | 28 | 79 | AKT1,BCL2,BCL2L1,BRAF,EGFR,ERBB2,FGF2,FGFR2,MTOR,GSK3B,IGF1R,IL6,JAK1,JAK2,KDR,MET,PDGFRA,PDGFRB,PIK3CA,PIK3R1,PRKCA,MAPK1,MAPK3,MAP2K1,RAF1,SRC,STAT3,VEGFA |
| hsa04931 | insulin resistance | 3.50793E-33 | 31 | 113 | AKT1,FOXO1,MTOR,GSK3B,GYS1,IKBKB,IL6,INSR,NFKB1,NOS3,PIK3CA,PIK3R1,PPARA,PRKCD,PRKCE,PRKCQ,PRKCZ,MAPK8,MAPK9,MAPK10,PTPN1,PTPN11,PYGL,RELA,RPS6KA3,STAT3,TNF,TNFRSF1A,NR1H2,OGT,NR1H3 |
| hsa05142 | Chagas disease (American trypanosomiasis) | 1.83715E-32 | 30 | 107 | AKT1,CASP8,CHUK,MAPK14,ACE,IKBKB,IL2,IL6,CXCL8,IRAK1,JUN,NFKB1,NOS2,SERPINE1,PIK3CA,PIK3R1,PPP2CA,MAPK1,MAPK3,MAPK8,MAPK9,MAPK10,RELA,TGFB1,TGFBR1,TLR4,TNF,TNFRSF1A,IRAK4,TLR9 |
| hsa05164 | Influenza A | 1.75172E-31 | 35 | 182 | AKT1,CASP1,CASP9,CREBBP,MAPK14,EP300,GSK3B,ICAM1,IKBKB,IL6,CXCL8,JAK1,JAK2,JUN,NFKB1,PIK3CA,PIK3R1,PLG,PRKCA,MAPK1,MAPK3,MAPK8,MAPK9,MAPK10,MAP2K1,PRSS1,RAF1,RELA,TLR4,TNF,TNFRSF1A,EIF2AK3,TBK1,IRAK4,NLRP3 |
|  |  |  |  |  |  |
